# Tricyclic Cell-Penetrating Peptides for Efficient Delivery of Functional Antibodies into Cancer Cells

**DOI:** 10.1038/s41557-021-00866-0

**Published:** 2022-02-10

**Authors:** Ole Tietz, Fernando Cortezon-Tamarit, Rod Chalk, Sarah Able, Katherine Vallis

**Affiliations:** 1 CRUK/MRC Oxford Institute for Radiation Oncology, Department of Oncology, University of Oxford, Old Road Campus Research Building, Roosevelt Drive, Oxford, OX3 7DQ, United Kingdom; 2 Centre for Medicines Discovery, University of Oxford, Old Road Campus Research Building, Roosevelt Drive, Oxford, OX3 7DQ, United Kingdom

## Abstract

The intracellular environment hosts a large number of cancer and other disease relevant human proteins. Targeting these with internalised antibodies would allow therapeutic modulation of hitherto undruggable pathways, such as those mediated by protein-protein interactions (PPI). However, one of the major obstacles in intracellular targeting is the entrapment of biomacromolecules in the endosome. Here we report an approach to delivering antibodies and antibody fragments into the cytosol and nucleus of cells using trimeric cell-penetrating peptides (CPP). Four trimers, based on linear and cyclic sequences of the archetypal CPP Tat, are significantly more potent than monomers and can be tuned to function by direct interaction with the plasma membrane or escape from vesicle-like bodies. These studies identify a tricyclic Tat construct that enables intracellular delivery of functional IgG antibodies and Fab fragments that bind intracellular targets in the cytosol and nuclei of live cells at effective concentrations as low as 1 μM.

## Graphical abstract

**Figure:**
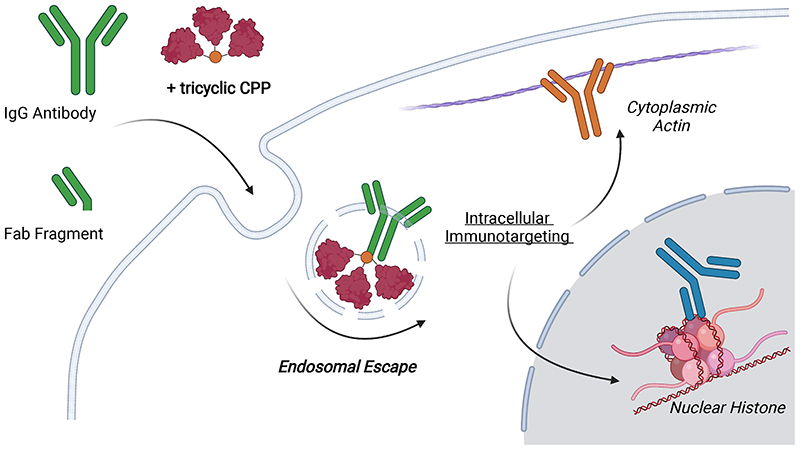

## Introduction

Agents that can deliver exogenous cargo into live cells are highly sought after and find wide application in cell biology; furthermore, effective intracellular targeting is crucial in the development of novel diagnostic and therapeutic agents.^1^ Intracellular delivery of functional monoclonal antibodies would allow the utilization of previously undruggable but therapeutically relevant targets, such as the large number of intracellular protein-protein interactions (PPI).^2^ Despite advances in the field, intracellular delivery remains a formidable challenge due to toxicity or limited efficacy of current vehicles. Cargo has been delivered into cells using viral and non-viral carriers, as well as physical techniques such as electroporation; each approach presents distinct strengths and weaknesses in the context of *in vitro*, *ex vivo* and *in vivo* applications.^3^ Polycationic non-viral carriers, such as polymers, lipid particles, and cell-penetrating peptides (CPPs), have been studied extensively over the past three decades as a means of transporting pharmacons into cells.^4-7^ The archetypal CPP Tat has been used extensively in the design of intracellularly targeted therapeutics; however, endocytic entrapment is recognized as a significant hindrance to intracellular delivery. ^8-13^ Low level leakage of Tat delivered cargos from endosomes is typically too inefficient to lend itself to most intracellular targeting applications.

A number of recent studies have attempted to address this problem.^14,15^ Cyclized Tat and oligoarginine conjugated to GFP (cTat-GFP) were found to access the cytosol and nucleus by direct translocation across the cell membrane.^16,17^ Linear oligoarginine was used as a reagent to deliver a range of cargos, including enzymes, nanobodies and antibodies, into cells by direct interaction with the plasma membrane.^18^ A CPP specifically designed to be endosomolytic (L17E) was shown to facilitate the escape of cargo from the endosome.^19^ A significant barrier to utilization of these approaches in translational *in vivo* applications is the relatively high concentration needed to achieve desirable results *in vitro* (>10 μM). Importantly, typical dosing of clinically used immunotherapeutics (10 mg/kg) results in peak antibody plasma concentrations of only 2 μM or less, emphasizing the need for CPP delivery technology with activity at ~ 1 μM. ^20^ The observation that Tat and other CPPs cluster prior to internalization,^21,22^ as well as recent insights into the relationship between charge density of polycationic agents and efficacy of delivery,^23-25^ led to the hypothesis addressed in the current report, that prearranging Tat peptides into multimeric clusters on a defined chemical scaffold would increase their efficacy. This hypothesis is lent credence by the observation that branched multimers of Tat, on peptide scaffolds or connected by disulphide bonds, results in increased intracellular delivery.^26-28^ Particular attention in this area of research has been given to bridged dimers of Tat and oligoarginine.^29-31^ Furthermore, cyclization of individual peptides has been shown to improve efficacy of uptake.^14^

Here we report the design and synthesis of four Tat trimers based on linear and cyclic sequences of Tat peptide, arranged on two different tetrakis core structures, which were evaluated *in vitro* using live cell confocal microscopy. The results indicate that the geometry of the synthetic core structure affects efficacy of delivery and that replacing linear Tat with a cyclized variant alters the mechanism of uptake. We identify a tricyclic lead structure and demonstrate delivery of functional antibodies and antibody fragments into the cytosol of HeLa cells which bind intracellular actin fibrils and fluorescent protein expressed in the cytosol and nuclei of live cells.

## Results

### Synthesis of Tat trimers

**Fig. 1:**
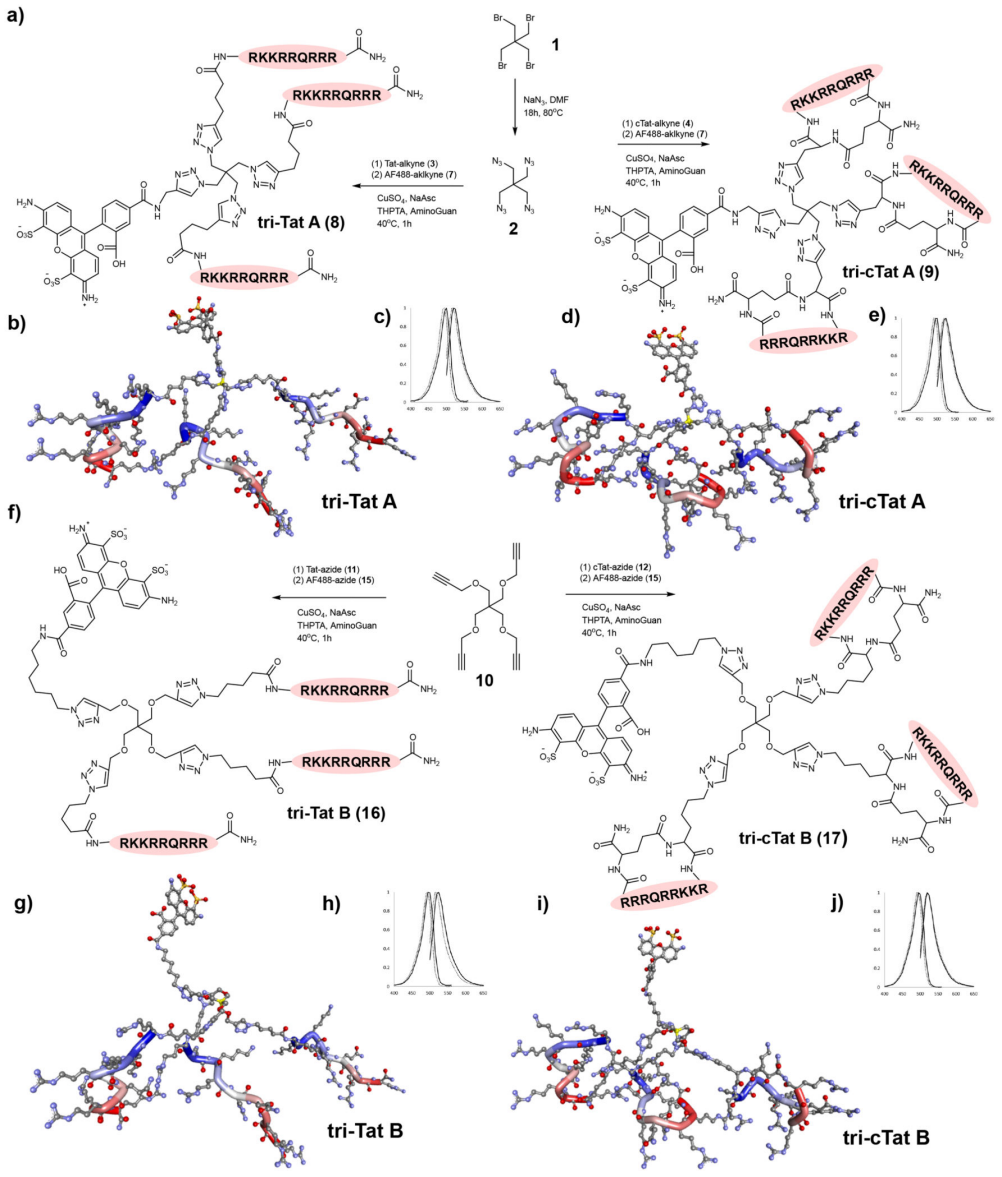
Synthesis of Trimer Tat constructs. Synthesis of tri-Tat A and tri-cTat A (**a**), tri-Tat B and tri-cTat B (**f**). *In silico* generated ball and stick models of linear Tat trimers suggest that they adopt coiled helices which arrange in a more compact conformation in tri-Tat A. Cyclic peptides adopt tighter helices, due to the C-terminus being forced towards the centre of the molecule. In all four trimers, peptides align in an off-parallel fashion, with arginine side chains pointing outwards. Hydrogens removed for clarity, Carbon – grey, Oxygen – red, Nitrogen – blue, Sulphur – orange; geometry of trimer conjugates optimized using a Dreiding-like forcefield; the peptide backbones highlighted from N-terminus (blue) to C-terminus (red); the central carbon of the tetrakis core highlighted in yellow. Fluorescence excitation and emission spectra of trimer (black line) compared to AF488 (dotted line); y axis - normalized fluorescence intensity (A.U.); x axis – wavelength (nm).

Four Tat trimers were designed using two different scaffolds furnished with either linear or cyclic Tat (cTat) peptide. These were chosen to determine (1) whether Tat trimers are more effective than monomers, (2) whether scaffold geometry affects the efficacy of Tat trimers, and (3) whether trimers based on cyclic sequences behave differently from trimers based on linear peptide. Tat-trimers were synthesized by copper catalysed 2+3 cyclo-addition between azide and alkyne functionalized components. The synthesis of trimers **tri-Tat A** and **tri-cTat A** is based on an azide functionalized scaffold A (**2,** Fig. 1a) and the synthesis of trimers **tri-Tat B** and **tri-cTat B** is based on an alkyne functionalized scaffold B (**10,** Fig. 1f). Linear Tat (49-57) peptide (RKKRRQRRR), modified with a hexynoyl (alkyne) (**3**) or an azido-pentanoyl (azide) (**11**) at the N-terminus was used to synthesize **tri-Tat A** (**8**) and **tri-Tat B** (**16**), as well as monomeric control **mono-Tat** (**18**, Supplementary Scheme 3). Cyclic Tat was custom designed to match the linear sequence; azide / alkyne functionality was added by including either an azide modified lysine (K(N3)) residue or propargyl glycine (Pra) at the N-terminus. Peptides were cyclized by addition of glutamic acid to the C-terminus for head to tail cyclisation to the N-terminus to generate cyclic Pra-RKKRRQRRRG (**4**) and K(N_3_)RKKRRQRRRG (**12**). These peptides were used to synthesize cyclic trimers **tri-cTat A** (**9**) and **tri-cTat B** (**17**), as well as monomeric cyclic control **mono-cTat** (**19**, Supplementary Scheme 3). Tat-trimers were labeled with AlexaFluor488 (AF488) for further study. Photophysical properties of peptide trimers were found to be similar to AF488 alone (Fig. 1; Supplementary Figs. 1 and 2). *In silico* generated three-dimensional representations of all four trimers are shown as ball and stick models in Figure 1.

### Uptake of trimers in HeLa and CHO cells

**Fig. 2:**
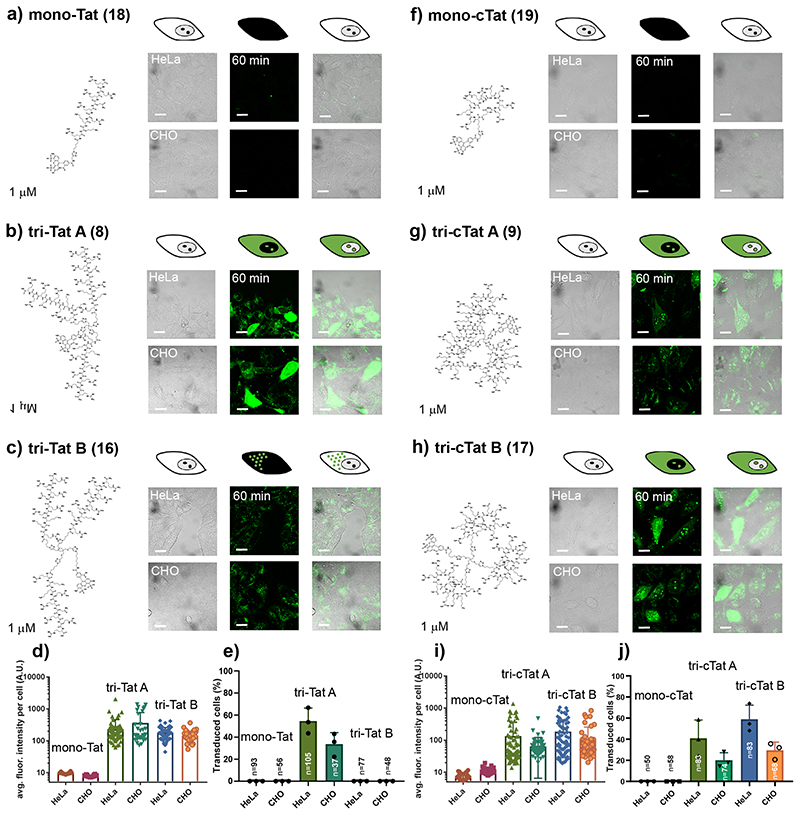
Live cell confocal microscopy of linear and cyclic Tat trimers in HeLa and CHO cells. (**a**, **f**) 1 μM mono-Tat (**a**) or mono-cTat (**f**) are not taken up into HeLa or CHO cells. (**b**, **g**, **h**) 1 μM linear tri-Tat A (**b**) cyclic tri-cTat A (**g**) and tri-cTat B (**h**) are taken up into the cytosol and nucleoli of HeLa and CHO cells. (**c**) 1 μM linear tri-Tat B is only taken up into endosomes of HeLa and CHO cells. (**d**, **i**) Quantification of average fluorescence intensity per cell in cells treated with linear (**d**) or cyclic (**i**) constructs. (**e**, **j**) Quantification of the percentage of transduced cells (scored as positive when showing homogenous cytoplasmic and nucleolar fluorescence) in cells treated with linear (**e**) or cyclic (**i**) constructs. Data presented as mean ± standard deviation; n-numbers identical for treatment groups analysed in graphs **d**/**e** and **i**/**j**. BF = Brightfield image. Scale bar: 20 μm.

The cellular uptake and intracellular distribution of Tat-trimers and monomeric controls was investigated by performing live cell confocal microscopy of HeLa (human, cervical cancer) and CHO (Chinese hamster, epithelial) cells incubated with Tat conjugates (1 μM) in serum-free DMEM (60 min). Following washing, images were acquired using a Zeiss 780 confocal microscope fitted with an incubation chamber (Fig. 2). In accordance with observations that free Tat in the cytosol readily enters the nucleus and strongly binds to nucleoli,^32^ homogenous staining of cytoplasm and nucleoli were considered as indicative of cells having accumulated free conjugate, not confined to endosomes, for quantification purposes. The results show that **mono-Tat** (1 μM) is not taken up by HeLa or CHO cells (Fig. 2a). In contrast **tri-Tat A**, shows homogenous cytosolic uptake and nucleolar staining at this concentration (Fig. 2b). This effect is not due to the increased total amount of Tat peptide, as experiments with 10 μM **mono-Tat** indicate no cytosolic uptake or nucleolar staining (Supplementary Fig. 3). The uptake and intracellular distribution of **tri-Tat A** is similar in live HeLa and CHO cells (Fig. 2b,d,e). Interestingly, experiments with **tri-Tat B** showed no evidence of cytosolic uptake or nucleolar staining in either HeLa or CHO cells (Fig. 2c,e). Experiments in HeLa cells transfected with the early endosomal marker rab5a tagged with RFP (rab5a-RFP) confirm that the intracellular punctuate fluorescence displayed by **tri-Tat B** is due to endosomal entrapment (Supplementary Fig. 5).

Similar to **mono-Tat**, and despite reports that cyclic Tat is more effective in comparison to linear Tat,^14,17^ **mono-cTat** is not taken up into HeLa or CHO cells (Fig. 2f). It is probable that differences between monomeric linear and cyclic Tat are only observed above certain threshold concentrations and thus not observed at 1 μM or 10 μM (Supplementary Fig. 3b). In contrast, **tri-cTat A** (1 μM) shows homogenous cytosolic and nucleolar staining (Fig. 2g), although the staining intensity is lower than that observed with linear **tri-Tat A**, and transduction efficiency is slightly reduced (Fig. 2i,j). Interestingly, **tri-cTat B** appears to be more effective at cellular transduction in comparison to **tri-cTat A** both by average fluorescence intensity per cell and percentage of transduced cells. As cell transduction and amount of trimer delivered into the cell differ for linear versus cyclic trimers, we propose that cyclic trimers enter the cytosol by a different mechanism from linear trimers. These initial experiments identified linear **tri-Tat A** and cyclic **tri-cTat B** as the most promising lead compounds.

### Time course of uptake in HeLa cells.

**Fig. 3:**
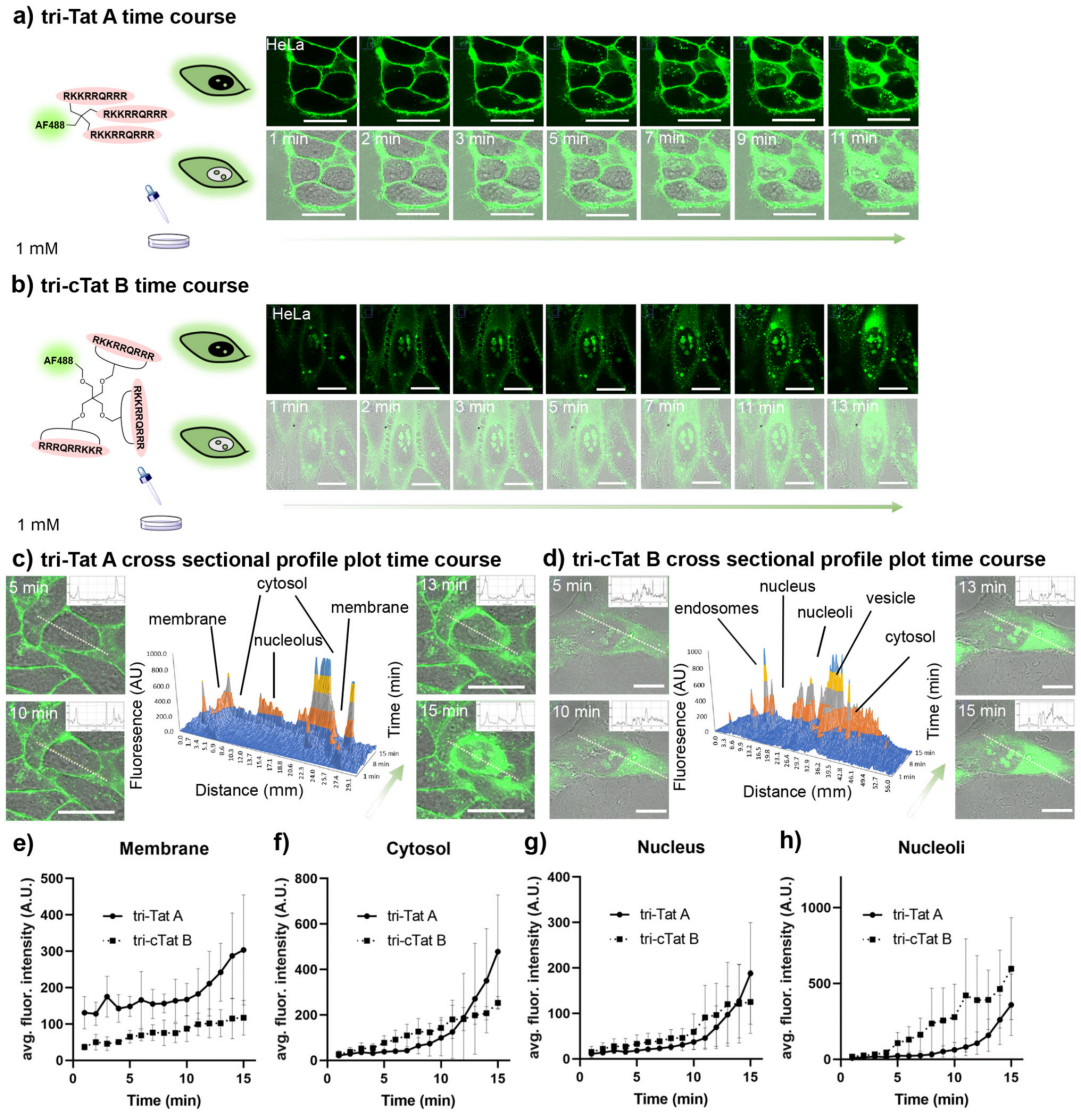
Continuous live cell confocal microscopy of trimers in HeLa cells. (**a**) Time course uptake of 1 μM tri-Tat A; and (**b**) 1 μM tri-cTat B. (**c**, **d**) Cross-sectional profile plot through a representative cell treated with 1 μM tri-Tat A (**c**) and 1 μM tri-cTat B (**d**); surface plot (middle) composed of cross-sectional profiles from 1 min to 15 min (y-axis: fluorescence intensity (AU); x-axis: distance along the cross section (mm); z-axis: time (min); colour added for clarity to denote y-axis values – dark blue 0-199 AU, orange – 200-399 AU, grey – 400-599 AU, yellow – 600-799 AU, light blue >800 AU). Tri-Tat A fluorescence moves from the plasma membrane inwards (**c**), while tri-cTat B fluorescence moves from a focal point in the cell outwards (**d**). (**e**-**h**) Time course analysis of cross-sectional profiles (1 – 15 min) through n=5 cells examined over 3 independent experiments treated with tri-Tat A (solid lines) and tri-cTat B (dotted lines), where regions of interest (ROIs) corresponding to the membrane (**e**), cytosol (**f**), nucleus (**g**), and nucleoli (**h**) are defined and average fluorescence intensity reported. Tri-cTat B shows significantly different membrane association and uptake kinetics into cellular compartments compared to tri-Tat A. Data presented as mean ± standard deviation. Scale bar: 20 μm.

To better understand the mechanism by which **tri-Tat A** and **tri-cTat B** enter the cytosol and the nucleus, we investigated the uptake kinetics of the conjugates during the first 15 min after their addition to HeLa cells using confocal live cell microscopy. **Tri-Tat A** or **tri-cTat B** (1 μM) were added to the cells and images acquired from 1 min to 15 min at 60 sec intervals (Fig. 3a,b). The two trimers show distinct uptake patterns in HeLa cells. To better understand the difference in uptake kinetics, we drew cross sectional profile plots through representative cells treated with **tri-Tat A** (Fig. 3c) and **tri-cTat B** (Fig. 3d). These plots reveal initial strong association of **tri-Tat A** with the membrane, indicated by two signals on either end of the plot, and no fluorescence in the cytosol; fluorescence signal then spreads from the membrane into the cytosol. In contrast, **Tri-cTat B** displays little association with plasma membrane and instead forms large vesicular bodies in the cytosol; fluorescence signal then spreads from these vesicles into the cytosol. To investigate whether findings in individual cells also apply to larger populations, we extended the plot profile analysis by designating regions of interest (ROI) corresponding to plasma membrane, cytosol (excluding endosomes), nucleus, and nucleoli along the cross section, using information from brightfield images.^33^ Average fluorescence intensities for each ROI, at each time point, in five cells per treatment group were calculated and plotted in Fig. 3, e–h. **Tri-Tat A** shows significant, approximately three-fold, stronger association with the plasma membrane (p<0.05 for t=1 to 15 min; Fig 3e). While the cytosolic signal of **tri-cTat B** gradually increases over time, cells treated with **tri-Tat A**, show little cytosolic accumulation (significantly lower than **tri-cTat B** from 4 to 7 min (p<0.05), until signal increases rapidly after ~ 12 min and exceeds **tri-cTat B** signal by a factor of 2 at 15 min (p=0.08, SI Table S2) (Fig. 3f). Similar patterns are observed for accumulation in the nucleus and nucleoli (Fig. 3g, h). These observations are consistent with different mechanisms of cell entry for the two trimers. **Tri-Tat A** translocates directly across the membrane, rather than or in addition to entry into the cytosol via endosomal escape, while the route of cell entry for **tri-cTat B** is the formation of vesicle-like bodies followed by vesicular escape.

To assess membrane integrity, 40 μM propidium iodide (PI; a cell impermeable dye), was added to HeLa cells 20 min after the addition of the peptide trimer. Cells with free **tri-Tat A** in the cytosol also contain PI (Extended Data Fig. 1a), indicating that transient pores are formed, once a critical peptide to membrane lipid ratio is reached,^21^ allowing the influx of PI into the cell (Supplementary Fig. 6). The amount of PI in cells, as measured by average fluorescence signal in the cytosol and nucleus, is significantly higher in cells treated with **tri-Tat A** than **tri-cTat B** (p<0.0001; Extended Data Fig. 1b, c), indicating that the cyclic peptide trimer does not form pores in the plasma membrane. Co-localization of **tri-cTat B** and rab5a confirms that the intracytoplasmic vesicle-like structures are endosomes; rab5a associates with endosomes before release of cargo into the cytosol (Supplementary Fig. 5).

Toxicity is a concern with potent intracellular delivery agents. We used the MTT assay to measure the metabolic activity of HeLa cells treated with 1 μM **tri-Tat A** or **tri-cTat B** for 60 min (Extended Data Fig. 1d). **Tri-cTat B** shows little acute toxicity (cells 94.3% viable, 4 h post-treatment), and no chronic toxicity (cells 96.5% viable, 3 days post-treatment). **Tri-Tat A** is slightly more toxic, which is consistent with the observation that the trimer forms membrane pores (cells 84.4% viable, 4 h post-treatment; 80.2% viable, 3 days post-treatment). The favourable uptake mechanism and low toxicity of **tri-cTat B** led us to investigate this agent for its potential to transport biomacromolecular cargos into cells.

### Co-delivery of antibodies and antibody fragments.

**Fig. 4:**
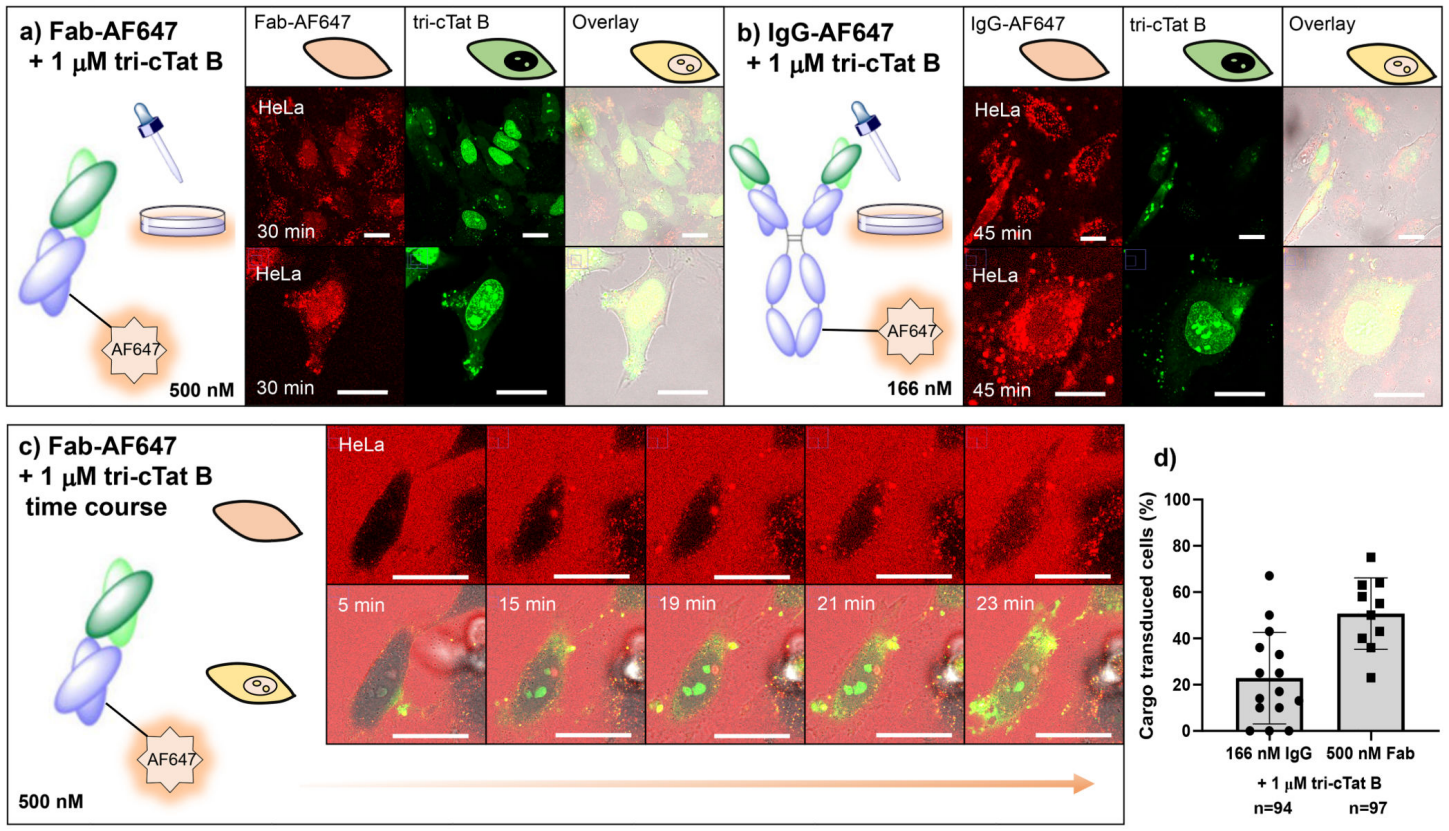
Co-delivery of antibodies and antibody fragments in live HeLa cells using tri-cTat B. (**a**, **b**) Post-wash live cell confocal microscopy images of HeLa cells treated with 500 nM mouse Fab fragment AF647 conjugate (Fab-AF647) and 1 μM tri-cTat B for 30 min (**a**) or 166 nM mouse IgG AF647 conjugate (IgG-AF647) and 1 μM tri-cTat B for 45 min (**b**) show homogenous distribution of Fab in cytosol and nucleus of cells with green nucleoli staining typical of tri-cTat B delivery; (**c**) Continuous live-cell confocal microscopy of trimers in HeLa cells treated with 500 nM Fab-AF647 (red) and 1 μM tri-cTat B (green); (**d**) quantification of the percentage of cells transduced with cargo (IgG-AF647 or Fab-AF647 - scored as positive when showing homogenous cytoplasmic and nucleolar fluorescence). Data presented as mean ± standard deviation. Scale bar: 20 μm.

Access to the cytosol by mechanisms that are independent of transmembrane pore formation constitutes a preferred method of co-delivery due to reduced cytotoxicity. Treatment of HeLa cells with 500 nM AF647-labelled non-specific mouse Fab fragment and 1 μM **tri-cTat B** for 30 min resulted in successful delivery of Fab into cytosol and nucleus of 50% of cells in a typical experiment (Fig. 4a, d). Successful delivery of Fab and its release from endosomes is indicated by homogenous staining of cytosol and nucleus by Fab-AF647; the persistence of some punctuate signal indicates that endosomal escape is incomplete. AF647 is only observed in cells that have been transduced with **tri-cTat B** showing that Fab internalisation depends on the action of the CPP (Supplementary Fig. 7). The time course of Fab uptake by **tri-cTat B** (Fig. 4c) follows a similar pattern as **tri-cTat B** itself (Fig. 3b, d). **Tri-cTat B** and Fab form large vesicle-like bodies that release their contents into the cytosol of the cell; unambiguous cytosolic staining by Fab is observed from 23 min. Interaction between peptide trimer and biomacromolecule, mediated by charge-charge interactions is likely to be important for the accumulation of both in endosomes and subsequent release and delivery of cargo into cells. Delivery of relatively β-sheet rich, neutrally charged recombinant RFP was unsuccessful (Supplementary Fig. 10) possibly due to a lack of association between **tri-cTat B** and cargo (Supplementary Fig. 11). On the other hand, interaction with the intended cargo diminishes the efficacy of the peptide trimer due to charge masking, emphasising the need to maintain an appropriate peptide to cargo ratio for a given macromolecule.

Experiments to explore the efficiency of **tri-cTat B** in the presence of serum-supplemented medium underline this principle. While **tri-cTat B** continues to function in the presence of serum, the number of transduced cells diminishes (61%, serum free; 42%, serum) but can be restored by increasing **tri-cTat B** concentration to 2 μM (79%); Supplementary Fig. 9. Whole IgG co-delivery by **tri-cTat B** is possible, but less efficient than the delivery of Fab, with 20% of cells showing homogenous AF647 staining in cytosol and nucleus (Fig. 4b, d). This is likely due to the larger size of antibodies and to the observation that antibodies have a greater tendency to aggregate in the presence of **tri-cTat B** compared to Fab fragments. There was no cellular uptake of Fab or IgG in the absence of **tri-cTat B** or in the presence of **mono-Tat** / **mono-cTat** (Supplementary Fig. 8).

We next investigated whether it is possible to deliver functional antibodies and antibody fragments into the cytosol and nuclei of cells. β-actin was chosen as a proof-of-principle target protein because of its abundance in the cell and formation of distinctive subcellular stress fibres (Supplementary Fig. 13). To clearly identify actin stress fibres, HeLa cells were transfected with red fluorescent protein fused to actin (actin-RFP) before being incubated with β-actin antibody (mouse IgG_2b_) or β-actin Fab fragment (mouse) conjugated to AF647 plus **tri-cTat B** for 30 min. After washing, cells were left for 60 min to allow IgG / Fab to bind to actin filaments. Figure 6a shows a representative cell transfected with actin-RFP (shown in orange, O), transduced with **tri-cTat B** and co-delivered β-actin Fab-AF647 (shown in red, R). Figure 6b shows the co-delivery of β-actin IgG-AF647 into a similarly treated cell. To enable objective quantification of co-localization between β-actin stress fibres and IgG / Fab fragment (white arrows), a co-localization threshold algorithm was used in selected ROI.^34^ Pearson’s and Manders’ coefficients were obtained under Costes’ automatic threshold^35^ and the co-localized pixels are shown as a mask of yellow pixels of constant intensity (co-localization panels). The significance of the co-localization parameters (Pearson’s and Manders’ coefficients) was evaluated using the confined displacement algorithm (CDA).^34,36^ This analysis indicates that 4 to 8% of RFP signal in ROIs co-localizes with IgG / Fab; while one third of IgG / Fab signal co-localizes with β-actin RFP (Supplementary Table 1), resulting in statistically significant correlation in the areas indicated (white arrows, yellow mask). These results indicate that β-actin Fab and IgG antibodies retain their ability to bind actin stress fibres in the cytoplasm of cells.

**Fig. 5:**
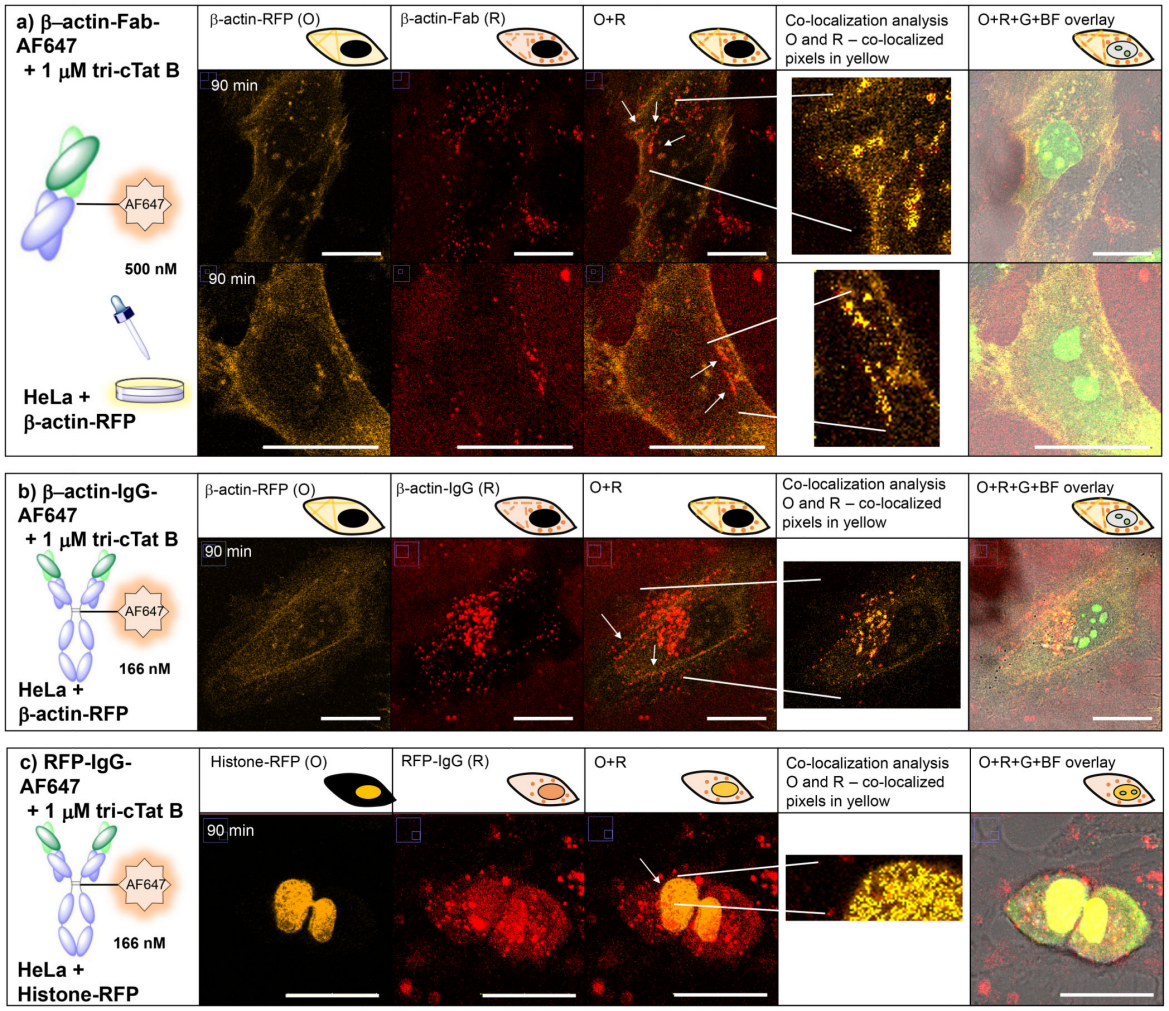
Co-delivery of functional antibodies and antibody fragments in live HeLa cells. HeLa cells were transfected with actin-RFP (**a**, **b**) or histone-RFP (**c**). 90 min post-wash live cell confocal microscopy images of cells treated with (**a**) 500 nM anti-β-actin mouse Fab-AF647 conjugate (β-actin-Fab-AF647) and 1 μM tri-cTat B for 30 min show co-localization of Fab (red) with actin stress fibres (orange); (**b**) 166 nM anti-β-actin mouse IgG_2b_ AF647 conjugate (β-actin-IgG-AF647) and 1 μM tri-cTat B for 30 min show co-localization of antibody with actin stress fibres; (**c**) 166 nM anti-RFP mouse IgG_1_ AF647 conjugate (RFP-IgG-AF647) and 1 μM tri-cTat B for 30 min show co-localization of antibody with RFP fused to histone in the nucleus. G = green channel (tri-cTat B); O = orange channel (RFP fusion protein); R = red channel (AF647); Co-localization panel: co-localized pixels are shown as a mask of yellow pixels of constant intensity and all results shown present a significant correlation and are co-localized; BF = brightfield; scale bar: 20 μm.

We went on to investigate whether functional antibodies can be delivered to the nucleus using **tri-cTat B**. HeLa cells were transfected with a histone-RFP fusion protein and then incubated with **tri-cTat B** and an anti-RFP IgG (mouse, IgG_1_; Fig. 5c). The antibody enters the nucleus and co-localizes with RFP (white arrows). Co-localization analysis of the ROI indicates that approximately half of the RFP signal co-localizes with IgG and >45% of IgG signal co-localizes with RFP, resulting in a significant correlation (Supplementary Table 1). It was also shown that anti-RFP IgG can bind actin-RFP stress fibres in the cytoplasm (Supplementary Figures 14 and 15), resulting in higher target occupancy (> 30%; Supplementary Table S1) compared to that achieved with β-actin-Fab-AF647. Some of the internalised antibody and Fab remains confined to vesicles that mainly localize in the perinuclear region. Trafficking of IgG and Fab to the nucleoli was observed, an off-target effect mediated by charge-charge association with **tri-cTat B** (Supplementary Fig. 17). Delivery of anti-RFP Fab fragment into the cytoplasm and nucleus of cells was accomplished, but co-localization could not be demonstrated, due to low affinity of the Fab fragment for RFP protein (Supplementary Fig. 12).

**Fig. 6:**
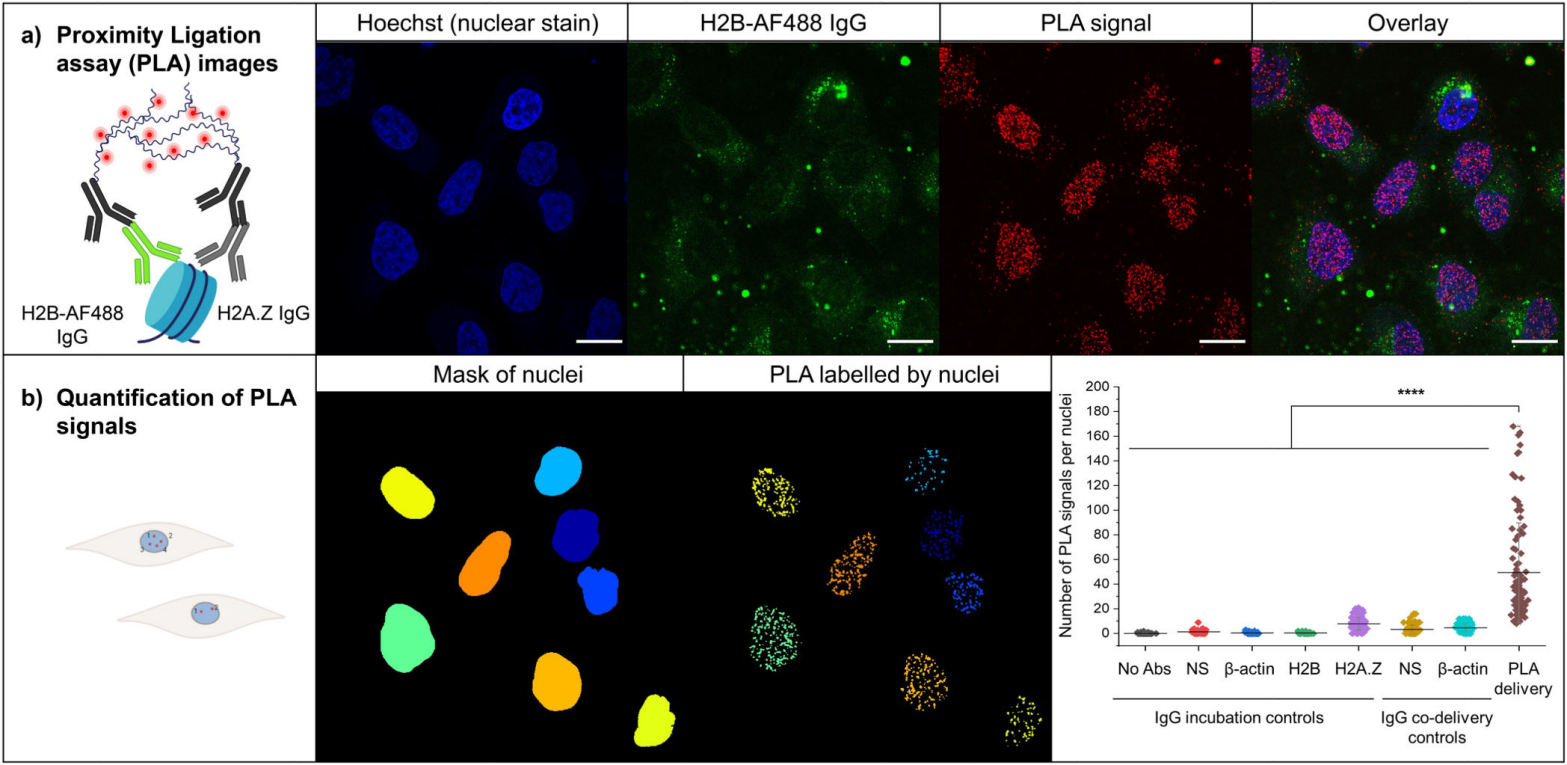
Proximity ligation assays (PLA). PLA were performed in HeLa cells using an H2B-AF488 antibody and a histone H2A.Z antibody. H2B-AF488 (166 nM) was co-delivered into cells using cTat_3_-alkyne (2 μM, 1h incubation at 37 °C, 5% CO_2_). Negative controls consisted in the co-delivery of a mouse non-specific (NS) antibody and an antibody against β-actin (166 nM IgG, 2 μM cTat_3_-alkyne, 1h incubation at 37 °C, 5% CO_2_) and in the incubation of non-specific, β-actin, H2B-AF488 and H2A.Z, antibodies after fixation and permeabilization of the cells and. a) Confocal microscopy images showing Hoechst 33342 stained nuclei (blue), PLA signals (red) and an overlay of the fluorescent channels. b) The number of PLA signals was quantified in the nuclei using CellProfiler and represented using Origin in at least 50 cells (IgG incubation controls: No abs, n=57; NS, n=55; β-actin, n= 76; H2B, n=55; H2A.Z, n=100. IgG codelivery controls: NS, n= 87; β-actin, n=90. PLA delivery, n=98) over three independent experiments. Data presented as mean ± SD. A. One way analysis of variance (ANOVA) with Tukey’s test correction were employed in statistical analysis. ****p<0.0001. Scale bars: 20 μm.

The proximity ligation assay (PLA; Duolink®) which allows optical detection of protein-protein interactions (PPI) was used to show that co-localization of CPP internalised antibodies and targeted RFP-tagged proteins results from specific interaction of the two. Samples are first incubated with antibodies against the two target proteins and then incubated with secondary antibodies with attached oligonucleotides that form a circular DNA structure only when the two primary antibodies are within 40 nm of each other. The circular DNA is amplified through addition of a complementary sequence that incorporates a fluorophore and thus reveals the presence of the PPI and its subcellular localization.^37^ PLA detects protein-protein interactions in their native form compared to other techniques such as co-immunoprecipitation, which requires disruption of the cell structure, or FRET, which requires extensive data processing. Here, the interaction between an anti-H2B-AF488, co-delivered to the cytosol and nuclei of HeLa cells using **tri-cTat B** lacking a fluorophore label (cTat_3_-alkyne, **14**), and an H2A.Z antibody, introduced in the cells after permeabilization, is observed. Proximity detection by secondary antibody mediated oligonucleotide ligation followed by rolling-circle amplification (RCA) reveals red spots in the nuclei of cells that also show nuclear accumulation of internalised H2B-AF488 IgG (Fig. 6a). The PLA signal in cells incubated with H2B-AF488 IgG plus **tri-cTat B** and then with H2A.Z IgG was significantly higher than in cells incubated with either H2B-AF488 or H2A.Z IgG alone (Fig. 6b; p<0.0001). Additional controls with a non-specific IgG and a β-actin IgG were performed by incubation of the cells as well as co-delivery of the antibodies using cTat_3_-alkyne. In all cases, it can be observed that the number of PLA signals in the nuclei remains significantly higher (Fig. 6b; p<0.0001) than in the incubated or co-delivered antibody controls. This provides evidence that anti-H2B IgG delivered into the nucleus by **tri-cTat B** is functional and binds the H2B/H2A.Z dimer that constitutes part of the nucleosome octamer. These results validate the findings of the co-localisation experiments and show that antibodies and antibody fragments delivered into the cytosol and nucleus using **tri-cTat B** retain their ability to bind target proteins.

## Discussion

The arrangement of Tat peptides into multimeric clusters on a defined chemical scaffold provides delivery agents that are significantly more effective than previously reported CPPs. The significant difference in transduction efficiency between the linear trimers **tri-Tat A** and **tri-Tat B** is surprising, but broadly consistent with reports suggesting that higher charge density leads to more effective uptake into cells.^23-25^ The interaction between trimer geometry and cell surface clusters might account for differences in uptake efficacy between different cell lines and raises the possibility of enhancing uptake into specific cell types by altering CPP cluster geometry. The effect of multimerization on binding efficacy to cell surface proteins has recently been demonstrated.^38^

Despite reports that cyclisation of CPPs can increase efficacy by up to two orders of magnitude,^39^ cyclic trimers **tri-cTat A** and **tri-cTat B** did not show significantly improved uptake into the cytoplasm and nuclei of cells compared to linear Tat trimers. Instead, cyclisation of Tat peptide on the trimer scaffold leads to a shift in mechanism from direct interaction with the plasma membrane to vesicular escape. A model, proposed by Pei and co-workers,^14^ argues that there is no mechanistic difference between linear and cyclic peptides of the same sequence and postulates an exclusively quantitative difference. The results of the current study, however, suggest that there is a qualitative difference in mechanism of uptake; trimerization of linear peptides and their cyclic equivalents adds a further element of complexity to the behaviour of CPPs *in vitro*. Two features of **tri-cTat B** uptake are broadly consistent with the proposals of Pei *et al*: (1) the formation of large vesicle-like bodies containing trimer and (2) the persistence of those vesicles while peptide spreads freely into the cytosol and nucleus.^14,39^

The data presented in this report indicate that, rather than gaining access to the cytosol by a single pathway, CPPs may be able to bind different cell surface components and are apt to switch between uptake mechanisms to find the most energetically favourable path into the cell. Our results show that it is possible to alter the mechanism of uptake by changing the geometry of conformation of Tat trimers, while leaving amino acid sequence and net charge unaltered.

It has been shown previously that endosomal escape is an efficient and nontoxic mechanism for intracellular cargo delivery.^19,29-31^ Successful co-delivery of antibodies and antibody fragments using **tri-cTat B**, is consistent with this. Our results further suggest that an appropriate peptide to cargo ratio is crucial for successful co-delivery. Charge-charge association between peptide and cargo is necessary for the formation of endosomes containing both agents followed by release, but also leads to charge masking and reduced efficacy of the CPP. These opposing effects need to be balanced for efficient delivery of macromolecules into cells. The relative structural homogeneity of antibodies and their fragments^40^ warrants the optimization of delivery protocols that are broadly applicable to these classes of cargo. Co-delivery of structurally diverse proteins is more challenging, as the results for RFP demonstrate. However, the absence of charge-charge interaction is likely to be a considerable advantage for covalently linked peptide-cargo conjugates, where high efficiency of delivery can be maintained, as previously demonstrated.^16^

The co-localization of protein targets and targeting agents in live cells provides insight into the efficacy of immunoglobulins in intracellular environments. While the fraction of cargo bound to target is high at 30% to 50%, the fraction of target occupied by cargo is dependent on the nature and abundance of the target. β-actin is a high abundance protein (>10 μM), with only a small fraction expressed as RFP fusion protein (in the experimental conditions shown in Figure 6). Intracellular cargo concentrations are at least 1 magnitude lower than target concentration; consequently, target occupancy is low (<10%). In contrast, histone-RFP expression is low, leading to relatively low abundance of RFP in the nucleus; as a result, higher target occupancy is observed (~ 45%). Indeed, using anti-RFP IgG, instead of anti-β-actin IgG, to target actin increases target occupancy to >30 %, due to the low abundance of RFP in stress fibres. These observations, combined with the sub nanomolar affinity of immunoglobulins for their targets, suggest that this approach might be most suited for the development of therapeutics against low abundance targets.

Proximity ligation assays detected binding of H2B-AF488 IgG, internalised in the presence of tri-cTat B, to the H2B/H2A.Z dimer that constitutes the histone octamer in the nucleus, demonstrating successful nuclear targeting and validating co-localization data. The mechanism by which antibodies enter the nucleus is not known; our data points to continuing interaction between tri-cTat B and IgG cargo in the cytosol of cells. It is possible that this association allows nuclear delivery of cargo using the nuclear localization sequence of the Tat peptide. Large cargos such as pathogens and pre-ribosomes (some up to 40 nm in diameter) are able to cross from the cytoplasm into the nucleus, suggesting that, based on size, whole IgGs which have a length of approximately 15 nm, could also do so.^41,42^ The ability of antibodies to enter the nucleus has been reported previously, particularly anti-DNA autoantibodies.^43,44^ Notably, a few fluorescent spots are located outside the nucleus in the proximity ligation assay (Fig. 6a). Histones, like other proteins, are synthesised in the cytoplasm, and are then transported into the nucleus as dimers (including H2A.Z-H2B).^45,46^ Therefore, a small amount of H2B and H2A.Z dimer does exist in the cytoplasm and was detectable in the proximity ligation assay following tri-cTat B-mediated internalisation of H2B-AF488 IgG.

**Tri-cTat B** is an efficient non-viral delivery agent allowing the rapid, non-toxic transport of biomacromolecular cargo into cells. This approach has some important advantages over viral delivery methods, which are limited to nucleic acid delivery and can trigger host immune responses, as well as mechanical delivery methods, which frequently lead to loss of cytoplasmic content, can excessively damage organic molecules, denature proteins, and are less amenable to *in vivo* translation.^3^ The successful targeting of actin stress fibres using β-actin antibodies and Fab fragments, as well as intranuclear RFP using an IgG, raises the possibility of using immunoagents against therapeutic targets inside the cytoplasm and nucleus to affect previously undruggable disease relevant pathways. In summary **tri-cTat B** can be used as an effective delivery agent to address the challenge of targeting intracellular therapeutic targets. Furthermore, the structure-activity insights reported here will aid the development of novel CPP multimers with tailored mechanistic properties.

## Methods

*Methods and additional data also available in* supporting information.

### Synthesis of Tat-conjugates

Peptide and fluorophores were conjugated to tetrakis scaffolds via copper-catalysed azide alkyne cycloaddition reactions (CuAAC), using azide / alkyne starting materials and 1 eq. CuSO_4_, 5 eq. tris((1-hydroxy-propyl-1H-1,2,3-triazol-4-yl)methyl)amine (THPTA), 2 eq. sodium L-ascorbate, 3 eq. aminoguanidine hydrochloride per cycloaddition, followed by HPLC purification and analysis by mass spectrometry. Azide functionalized scaffold A (1,3-diazido-2,2-bis(azidomethyl)propane, **2**) was used to synthesize **Tri-Tat A** and **tri-cTat A**; alkyne functionalized scaffold B (tetakis(2-propynyloxymethyl)methane, **10**) was used to synthesize **tri-Tat B** and **tri-cTat B**. Linear Tat (49-57) peptide (RKKRRQRRR), modified with a hexynoyl (alkyne) (**3**) or an azido-pentanoyl (azide) (**11**) functional group at the N-terminus was obtained from International Peptides Inc., Louisville, KY, USA. Cyclic Tat was custom designed to match the linear peptide and synthesized by Cambridge Peptides, Birmingham, UK. Azide / alkyne functionality was added by including either an azide modified lysine (K(N_3_)) residue or propargyl glycine (Pra) at the N-terminus. Peptides were cyclized by addition of glutamic acid to the C-terminus for head to tail cyclisation to the N-terminus to generate cyclic Pra-RKKRRQRRRG (**4**) and K(N3)RKKRRQRRRG (**12**).

### Live cell confocal microscopy

HeLa (obtained from ATCC; CCL-2) and CHO (obtained from institute repository) cells were cultured in DMEM supplemented with 10% FBS and penicillin / streptomycin. For post-wash experiments, Tat conjugate was added to the cells in serum-free medium, cells incubated at 37 °C in an atmosphere of 5% CO_2_, culture medium removed at the desired time point, cells washed and transferred to the microscope for imaging. For time course or pre-wash experiments, cells were transferred to the microscope (incubation chamber at 37 °C, 5% CO_2_) and Tat conjugate added directly to cell medium and cells imaged at desired time points. Co-delivery experiments were conducted by adding peptide trimer immediately followed by cargo into serum free medium. Samples were visualised using a Zeiss LSM 780 inverted confocal microscope fitted with a XLmultiS1 incubation chamber and Plan-Apochromat 63x / 1.40 Oil DIC M27 objective. Images were analysed using ImageJ software. Digital adjustments and image processing are consistent for all experiments, unless otherwise stated in the figure legend. Statistical analyses were carried out using GraphPad Prism 7 or Origin software and all data used for statistical analysis consists of measurements from distinct cells. Where applicable a parametric, unpaired, two-tailed t-test was used for statistical testing.

## Extended Data

**Extended Data Fig. 1:**
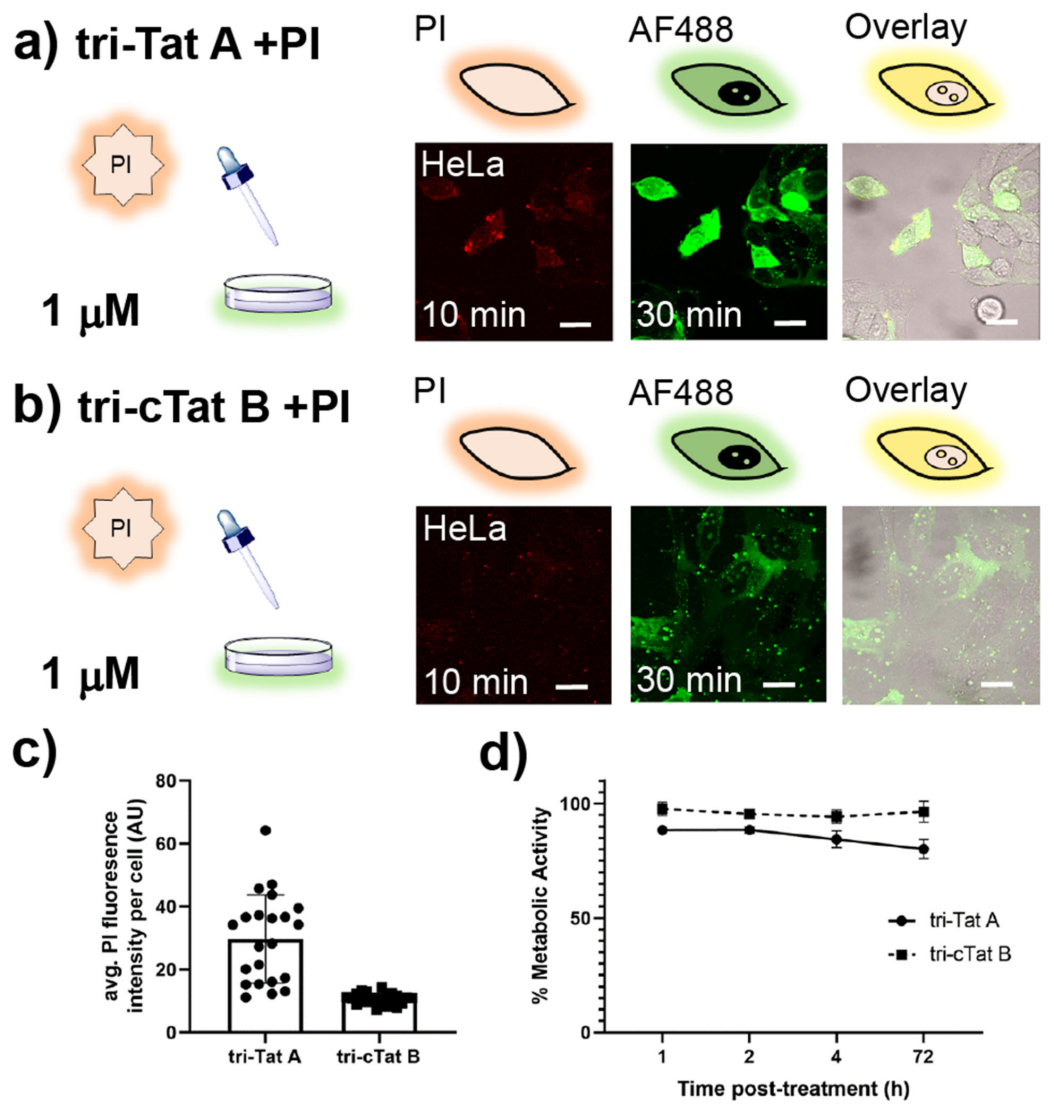
Membrane porosity following treatment with Tat-trimer. (**a**, **b**) Addition of 40 μM propidium iodide (PI) 20 min after addition of 1 μM trimer; image at 30 min after start of experiment. Cells treated with tri-Tat A (**a**) co-stain with PI; cells treated with tri-cTat B (**b**) are PI negative. (**c**) Average fluorescence intensity of PI per cell, 45 min after the start of the experiment (n=25). Cells treated with tri-Tat A show significantly higher PI uptake, indicative of pore formation. (**d**) Cells treated with tri-Tat A (solid line) or tri-cTat B (dotted line) for 60 min and metabolic activity as an indicator of cell viability assessed using MTT assay after 1 h, 2 h, 4 h, 3 days (n=3 biologically independent experiments). Data presented as mean ± standard deviation. Scale bar: 20 μm.

## Supplementary Material

Statistical Source Data Extended Figure 1

Statistical Source Data Figure 1

Statistical Source Data Figure 2

Statistical Source Data Figure 3

Statistical Source Data Figure 4

Statistical Source Data Figure 6

Supporting Information

## Data Availability

The authors declare that all the data supporting the findings of this study are available within the Article, the Supplementary Information, or the Source Data. Alternatively the data is also available from the corresponding authors upon reasonable request.
