# Supplementary material for: Tricyclic Cell-Penetrating Peptides for Efficient Delivery of Functional Antibodies into Cancer Cells
Source: Nat Chem. Author manuscript; Available in PMC 2024 Dec 7. (PMC7617065; doi:10.1038/s41557-021-00866-0)
Supplement: Supporting Information [file EMS139797-supplement-Supporting_Information.pdf]

Supporting Information

Tricyclic Cell-Penetrating Peptides for Efficient Delivery of Functional Antibodies into Cancer Cells

Ole Tietz[1], Fernando Cortezon-Tamarit[1], Rod Chalk[2], Sarah Able[1], Katherine Vallis[1]*

[1] CRUK/MRC Oxford Institute for Radiation Oncology, Department of Oncology, University of Oxford, Old Road Campus Research Building, Roosevelt Drive, Oxford, OX3 7DQ, United Kingdom.

[2] Centre for Medicines Discovery, University of Oxford, Old Road Campus Research Building, Roosevelt Drive, Oxford, OX3 7DQ, United Kingdom.

CONTACT: * telephone: +44 (0)1865 2 25850; e-mail: katherine.vallis@oncology.ox.ac.uk

Table of contents

S1. Experimental materials and instruments **5**

S2. Synthesis and characterization of Tat conjugates **6**

S2.1. General procedure for copper catalyzed 2+3 cycloaddition reactions **8**

S2.2. Compound **2** **9**

S2.3. Compound **5** **10**

S2.4. Compound **6** **12**

S2.5. Compound **8 (tri-Tat A)** **14**

S2.6. Compound **9** (**tri-cTat A**) **16**

S2.7. Compound **13** **18**

S2.8. Compound **14** **20**

S2.9. Compound **16** (**tri-Tat B**) **22**

S.2.10. Compound **17** (**tri-cTat B**) **24**

S.2.11. Compound **18** (**mono-Tat**) **26**

S.2.12. Compound **19** (**mono-cTat**) **28**

S3. Photophysical properties of Tat conjugates **30**

S3.1. UV absorbance spectra **30**

S3.2. Fluorescence excitation / emission spectra **31**

S4. Cell culture / preparation **32**

S5. Live cell confocal microscopy **32**

S6. Supplementary live cell confocal microscopy images **34**

S6.1. mono-Tat (18) / mono-cTat (19) (10 μM) in HeLa and CHO cells **34**

S6.2. Co-localization of tri-Tat A (8), tri-Tat B (16) and tri-cTat B (17) with rab5a-RFP as an early endosome marker **35**

S6.3. Co-staining of tri-Tat A and PI in HeLa cells **37**

S6.4. Release of tri-cTat B and Fab in HeLa cells **38**

S6.5. Control experiment: IgG / Fab uptake into HeLa cells without Tat-trimer treatment **40**

S6.6. tri-cTat B (1 μM / 2 μM) delivery in the presence of serum in HeLa cells **42**

S6.7. Co-delivery of Red Fluorescence Protein (RFP) using tri-cTat B in HeLa cells **43**

S7. Antibody conjugation **45**

S7.1. IgG antibody conjugation **45**

S7.2. Fab fragment conjugation **45**

S.7.3. Anti-β-actin IgG antibody **45**

S.7.4. Anti-RFP IgG antibody **45**

S7.5. Synthesis of Fab fragment **46**

S.7.6. Anti-β-actin Fab fragment **46**

S.7.7. Anti-RFP Fab fragment **46**

S7.8. Binding of RFP Fab fragment to recombinant RFP **46**

S8. Co-delivery of Cargo and co-localization analysis **47**

S8.1. RFP fusion protein transfection of HeLa cells **47**

S8.2. IgG / Fab delivery using tri-cTat B **48**

S8.3. Co-localization Analysis **49**

S8.4. Additional co-localization images **49**

S8.4.1. Actin stress Fibres **49**

S8.4.2. Co-localization of RFP-labelled actin stress fibres with anti-RFP IgG **51**

S8.4.3. Additional Images anti-β-actin IgG / actin stress fibre co-localization **52**

S8.4.4. Additional Images anti-RFP IgG / histone-RFP co-localization **52**

S8.4.5. Whole cell co-localization analysis **53**

S8.5. Pearson & Manders' coefficient Summary **55**

S9. Quantitative Analysis of mechanism of uptake **55**

S10. Proximity ligation assay protocol **62**

S11. References **63**

S1. Experimental materials and instruments

All reagents and solvents were obtained from Sigma-Aldrich, unless otherwise stated and used without further purification. Preparative chromatographic separations were performed on Aldrich Science silica gel 60 (35-75 µm) and reactions followed by TLC analysis using Sigma-Aldrich silica gel 60 plates (2-25 µm) with fluorescent indicator (254 nm) and visualized with UV. ^{1}H and ^{13}C nuclear magnetic resonance spectra were recorded in Fourier transform mode at the field strength specified on a Bruker Avance 300, 400 or 500 MHz spectrometer. Spectra were obtained from the specified deuterated solvents in 5 mm diameter tubes. Chemical shift in ppm is quoted relative to residual solvent signals calibrated as follows: **$CDCl_3$** δ_H ($CHCl_3$) = 7.26 ppm, δ_C = 77.2 ppm; **$(CD_3)_2SO$** δ_H (CD_3SOCHD_2) = 2.50 ppm, δ_C = 39.5 ppm; **MeOD-d_4** δ_H (CD_2HOD) = 3.31 ppm, δ_C = 49.0 ppm; **$(CD_3)_2CO$** δ_H (CD_2COCHD_2) = 2.09 ppm, δ_C ($(CD_3)_2CO$) = 30.6 ppm. Multiplicities in the ^{1}H NMR spectra are described as: s = singlet, d = doublet, t = triplet, q = quartet, quint. = quintet, m = multiplet, b = broad; coupling constants are reported in Hz. Mass spectra for small molecules were recorded on a Waters Micromass LCT instrument.

Accurate mass of Tat conjugates was determined as follows. Samples were diluted to a final concentration of 1 µM in 0.1 % formic acid. 50 µl was injected on to a 1290 UPLC coupled to a 6530 QTOF mass spectrometer fitted with a 2.1 mm x 12.5 mm Zorbax 5 µm 300SB-C3 guard column (Agilent Technologies, Santa Clara, USA). Solvent A was 0.1 % formic acid in HPLC grade water; solvent B was 0.1 % formic acid in LC-MS grade methanol (Fisher Scientific, Loughborough, UK). Initial conditions were 10% B at 1 mL/min. A methanol gradient was developed from 10% to 80% over 34 seconds, followed by isocratic at 95% B for 41 seconds, followed by 15 seconds re-equilibration at 10% B. The mass spectrometer was operated in positive ion, 2 GHz

detector mode. Source parameters were drying gas 350°C, flow 12 L/min, nebulizer 60 psi, capillary 4000 V. Fragmentor was 250 V, collision energy 0 V and data acquired from 100-3200 m/z. Data analysis was performed using Masshunter Qualitative Analysis B0.7 proprietary software and deconvolution performed using the Maximum entropy method (with the exception of **mono-Tat** (**18**) and **mono-cTat** (**19**), which were deconvoluted by resolved isotope method). HPLC runs were carried out on an Agilent 1260 Infinity II system fitted with quaternary pump modul, vialsampler, multicolumn thermostat, diode array detector and analyt fraction collector (Agilent Technologies, USA). HPLC columns were kept at 30°C, chromatograms were collected at 210, 230, 250, 254, 280 nm, as well as from 440 nm to 550 nm (at 5 nm intervals) using a deuterium lamp. In silico models of Tat-trimers 8, 9, 16 and 17 were generated using BIOVIA Discovery Studio Visualizer v17.2. (Dassault Systemes) and optimized using a Dreiding-like forcefield.

S2. Synthesis and characterization of Tat conjugates

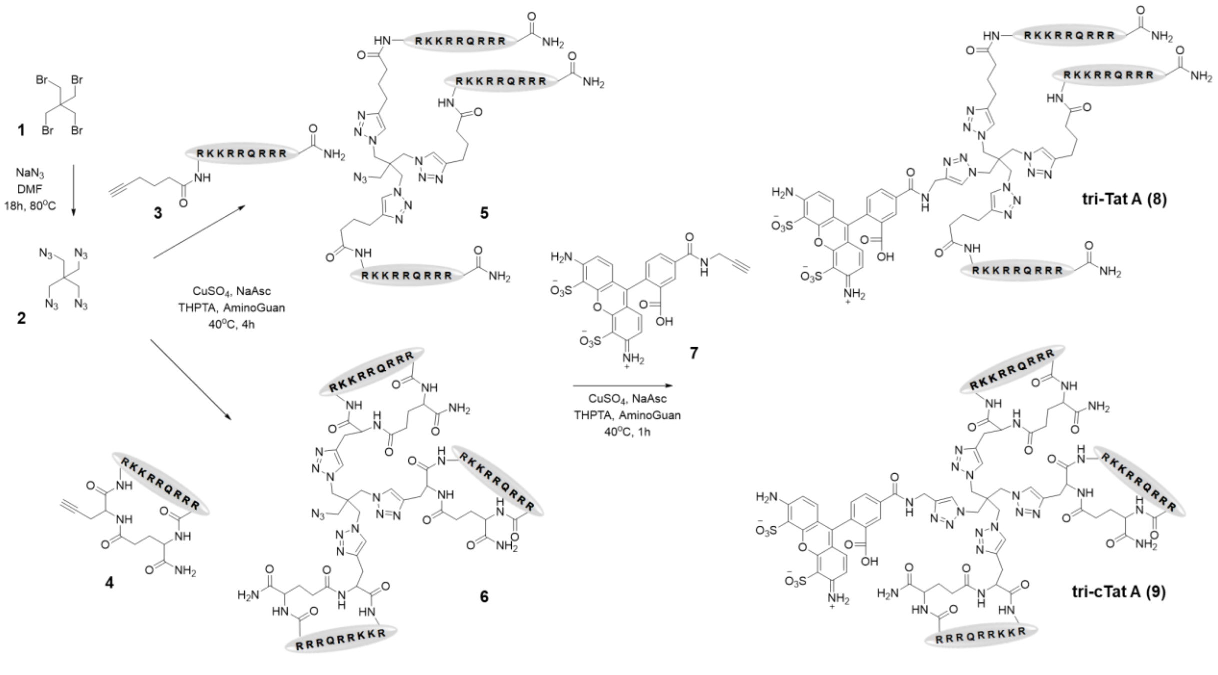

Scheme S1: Synthesis of **tri-Tat A** (**8**) and **tri-cTat A** (**9**)

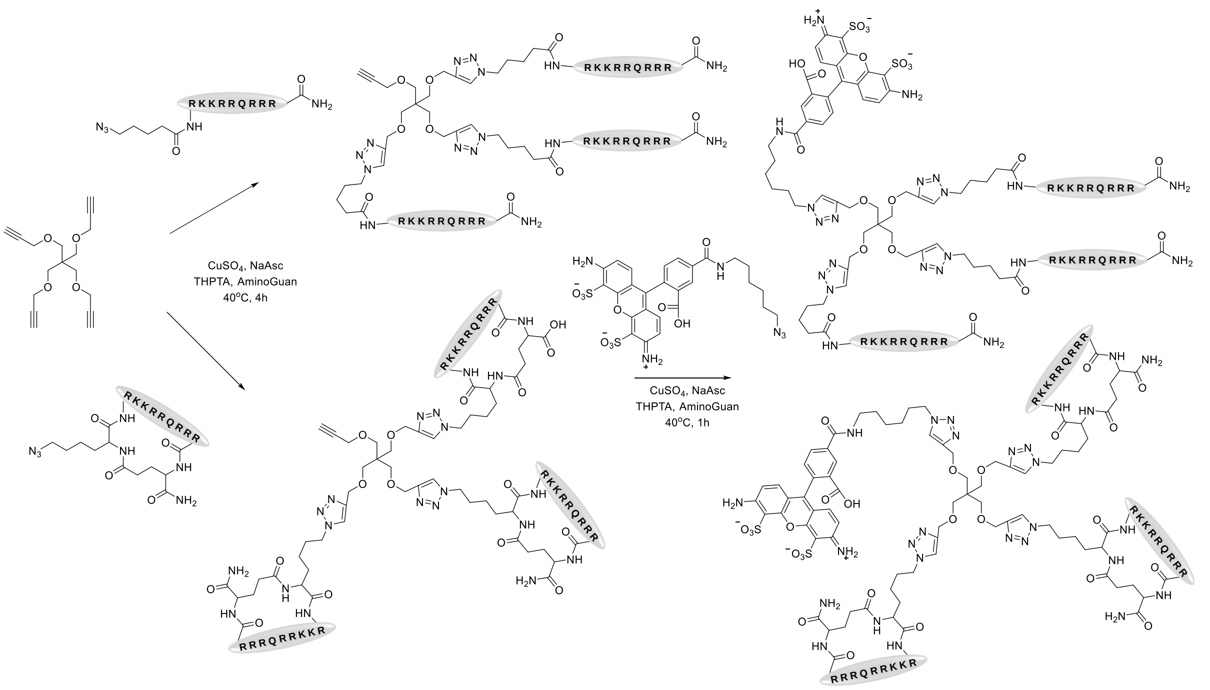

Scheme S2: Synthesis of **tri-Tat B** (**16**) and **tri-cTat B** (**17**)

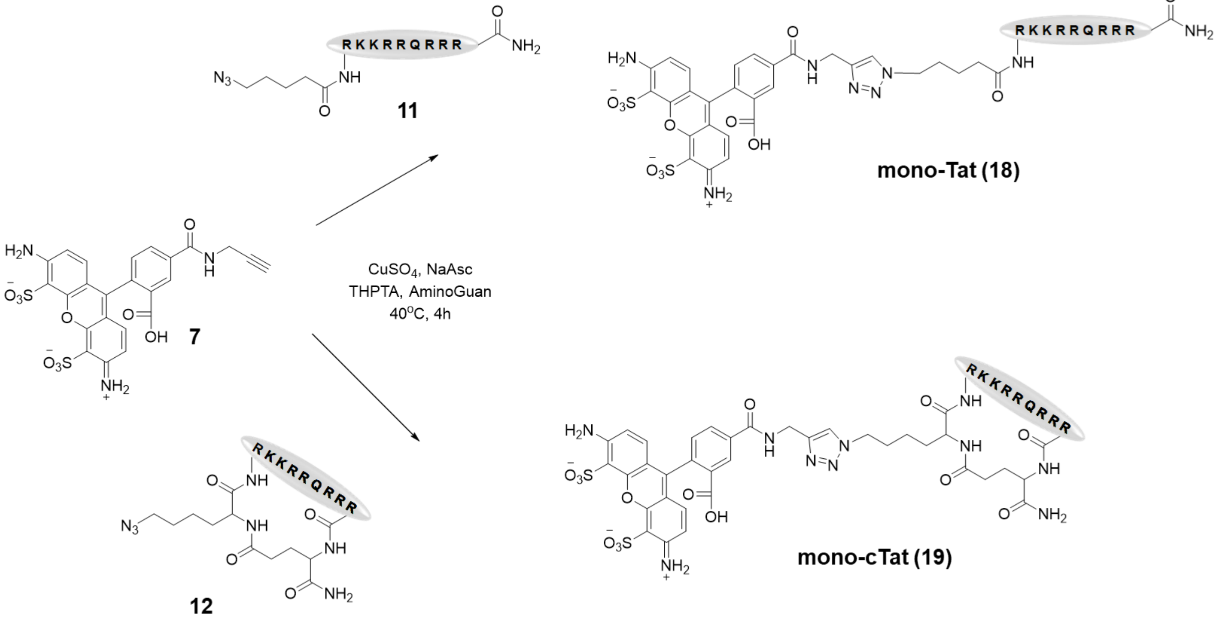

Scheme S3: Synthesis of **mono-Tat** (**18**) and **mono-cTat** (**19**)

S2.1. General procedure for copper catalyzed 2+3 cycloaddition reactions (Compounds 5, 6, 8, 9, 13, 14, 16-19).

Peptide and fluorophores were conjugated to tetrakis scaffolds using copper-catalyzed azide alkyne cycloaddition reactions (CuAAC). 5-hexanoyl-Tat-NH_2 (**3**, Tat-alkyne (49-57)) and 5-azido-pentanoyl-Tat-NH_2 (**11**, Tat-azide (49-57)) were obtained from Peptide International Inc., USA. Propargyl-glycine-cyclicTat-NH_2 (**4** - cTat-alkyne) and azidolysine-cyclicTat-NH_2 (**12**, cTat-azide (49-57)) were synthesized by Cambridge Peptides (UK), using Fmoc solid-phase peptide synthesis, followed by glutamic acid to N-terminus head to tail cyclisation and HPLC purification. Optimized CuAAC reaction conditions include shaking at 1,000 rpm and heating at 40^oC for 60 to 240 min using a thermoshaker. Molar ratios of reagents were normalized to the number of CuAAC per molecule and are quoted for each reaction specifically, in general 1 eq. $CuSO_4$, 5 eq. Tris((1-hydroxy-propyl-1H-1,2,3-triazol-4-yl)methyl)amine (THPTA), 2 eq. Sodium L-ascorbate, 3 eq. Aminoguanidine hydrochloride were used per CuAAC. $CuSO_4$, THPTA, Sodium L-ascorbate and Aminoguanidine HCl were dissolved in water immediately prior to use, AF488-azide and AF488-alkyne were dissolved in DMSO, peptides dissolved in water and used from frozen stocks. Reactions were carried out in Ca / Mg-free Dulbecco's phosphate-buffered saline (PBS) with 30% DMSO. Reactions were quenched using EDTA (final concentration 10 mM) and HPLC purified using a Waters XBridge C18 (3.5 μm, 4.6 x 150 mm) analytical column or an Agilent Polaris C18-A (3.0 μm, 4.6 x 150 mm) analytical column. HPLC purification and quality control samples were run using a 40 min method, flow rate = 1 mL/min, gradient rising from 1% CH_3CN (0.1% TFA) / H_2O (0.1% TFA) to 40% CH_3CN (0.1% TFA) / H_2O (0.1% TFA) over the course of 40 min. Fractions containing compound of interest were neutralized using PBS and solvent exchanged using Amicon Ultra 0.5

mL centrifugal filters (Ultracel – 3k) (Merck Millipore) by washing with PBS once followed by three washes with H_2O.

S2.2. Compound 2

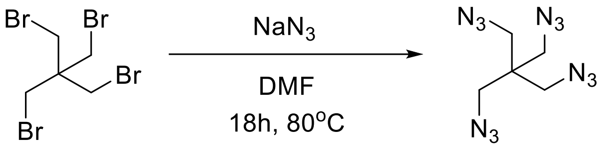

1
$C_5H_8Br_4$
(383.74)

2
$C_5H_8N_{12}$
(236.10)

1,3-diazido-2,2-bis(azidomethyl)propane (2).

1,3-dibromo-2,2-bis(bromomethyl)propane (**1**) (200 mg, 0.52 mmol) and NaN_3 (170 mg, 2.60 mmol, 5 eq) were briefly dried under high vacuum and dissolved in DMF (5 ml). The reaction was heated under a constant flow of Argon at 80°C for 18 h. The reaction mixture was separated between $CHCl_3$ and H_2O, the organic layer washed with H_2O (x3) and brine (x3) and dried over anhydrous Na_2SO_4. Solvent was removed in vacuo and the product purified by preparative TLC in 10 % EtOAc/hexane (rf ~ 0.5). (NB: An azide stain was used to interpret analytical TLC plates, consisting of 1:1 propargylalcohol / EtOH and Cu(I)I, followed by treatment with a heat gun; azide containing compounds are stained white in comparison to brown stain on the rest of the plate.) The product was obtained as a white solid (100.3 mg, 0.425 mmol, 81.7 % yield).

^{1}H-NMR (300 MHz, $CDCl_3$): 3.29 (s, 8H, 4 x CH_2); ^{13}C-NMR (75 MHz, $CDCl_3$): 44.1, 51.7; LCMS: 236.2 [ES+].

S2.3. Compound 5

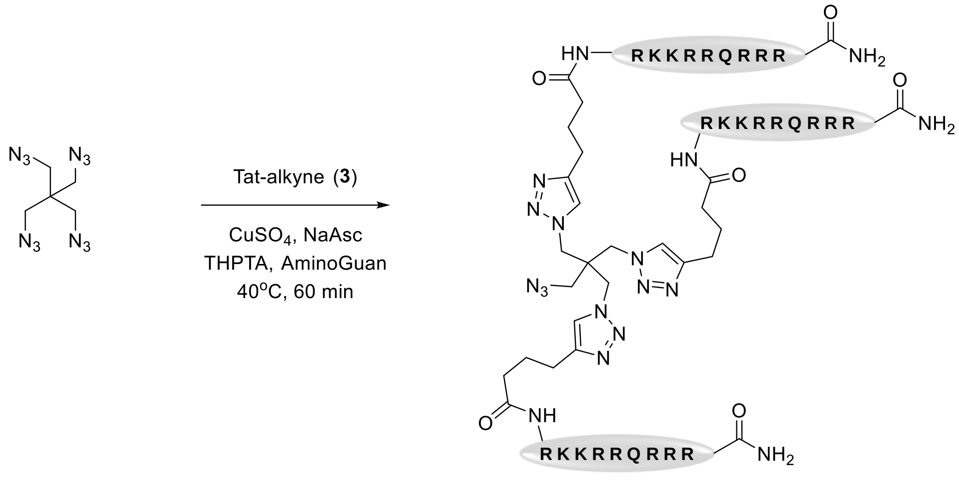

2
$C_5H_8N_{12}$
(236.20)

5
$C_{182}H_{347}N_{105}O_{33}$
(4534.48)

Tat$_3$-N$_3$ (5)

1,3-diazido-2,2-bis(azidomethyl)propane (**2**) (38.5 μg, 0.16 μmol, 1 eq.) and 5-hexanoyl-Tat-NH_2 (**3** - Tat-alkyne (49-57)) (Peptide International Inc., USA) (1000 □g, 0.70 □mol, 4.2 eq.) as well as CuAAC regents were dissolved in 30 % DMSO / PBS and heated at 40°C for 60 min. The product was purified by HPLC (294 μg, 0.06 μmol, 41 % yield).

HPLC retention time r_t = 17:40 min; MS QTOF [ES+]: 4534.85 (calculated: 4534.48 for $C_{182}H_{347}N_{105}O_{33}$).

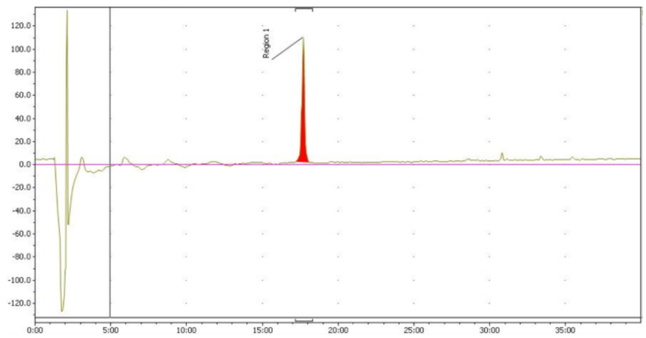

HPLC trace of Tat$_3$-N$_3$ (**5**) at 250 nm

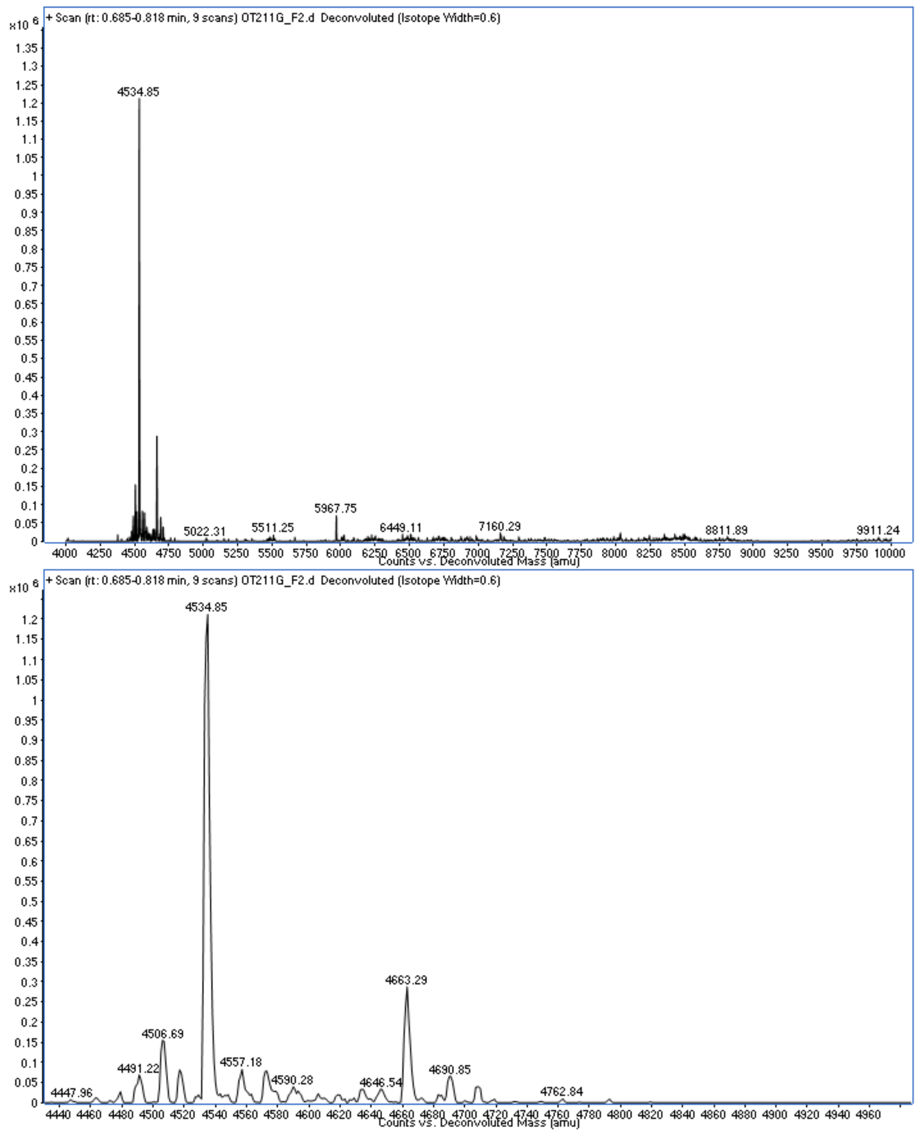

Maximum entropy deconvoluted MS QTOF mass spectra of Tat_3-N_3 (**5**).

S2.4. Compound 6

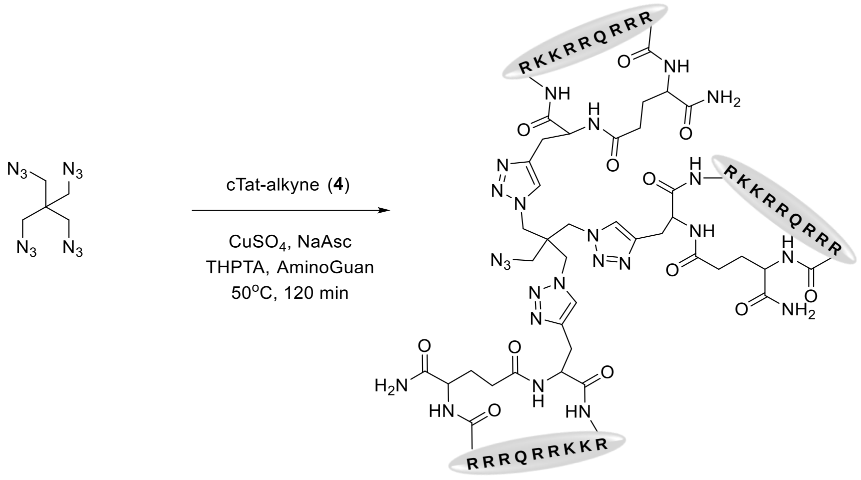

2 $C_5H_8N_{12}$ (236.20)

6 $C_{194}H_{359}N_{111}O_{39}$ (4870.74)

cTat$_3$-N$_3$ (6)

1,3-diazido-2,2-bis(azidomethyl)propane (**2**) (35.8 μg, 0.15 μmol, 1 eq.) and propargyl-glycine-cyclicTat-NH_2 (**4** - cTat-alkyne) (Cambridge Peptides, UK) (1100 μg, 0.71 μmol, 4.7 eq.), as well as CuAAC regents were dissolved in 30 % DMSO / PBS and heated at 50°C for 120 min. The product was purified by HPLC (230 μg, 0.05 μmol, 31 % yield).

HPLC retention time r_t = 19:16 min; MS QTOF [ES+]: 4871.88 (calculated: 4870.74 for $C_{194}H_{359}N_{111}O_{39}$).

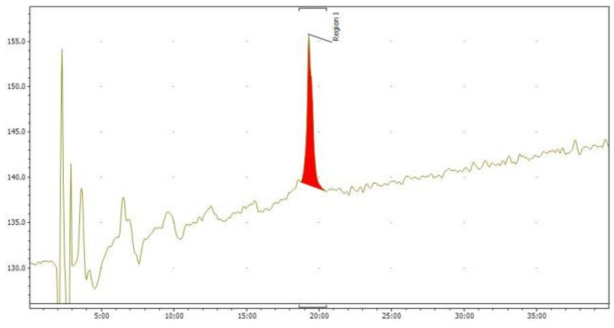

HPLC trace of cTat$_3$-N$_3$ (**6**) at 250 nm.

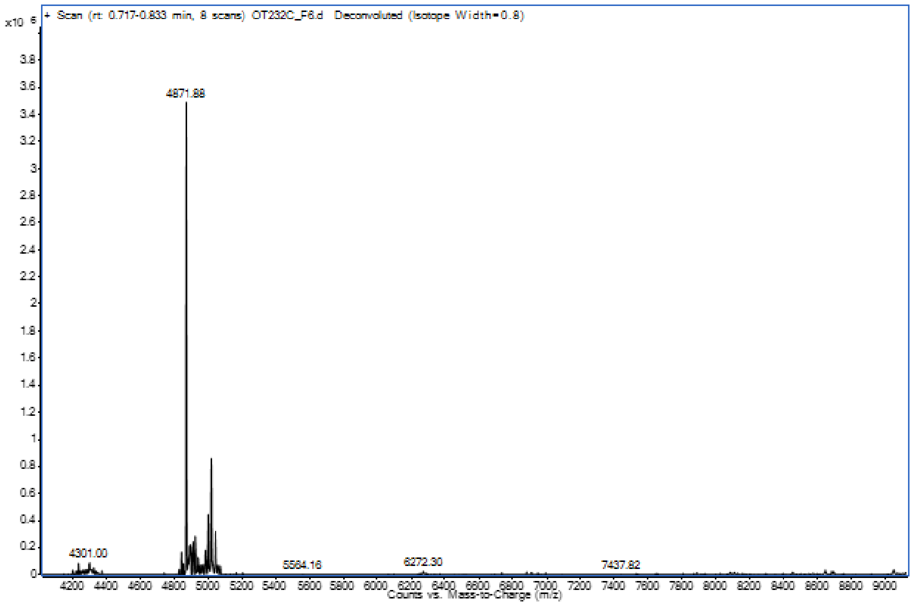

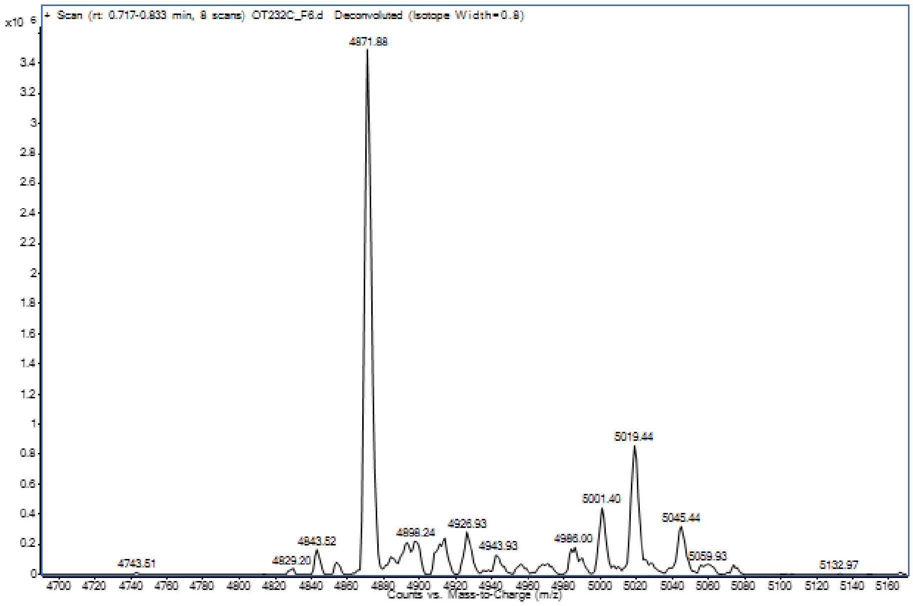

Maximum entropy deconvoluted MS QTOF mass spectra of **cTat$_3$-N$_3$ (6)**

S2.5. Compound 8

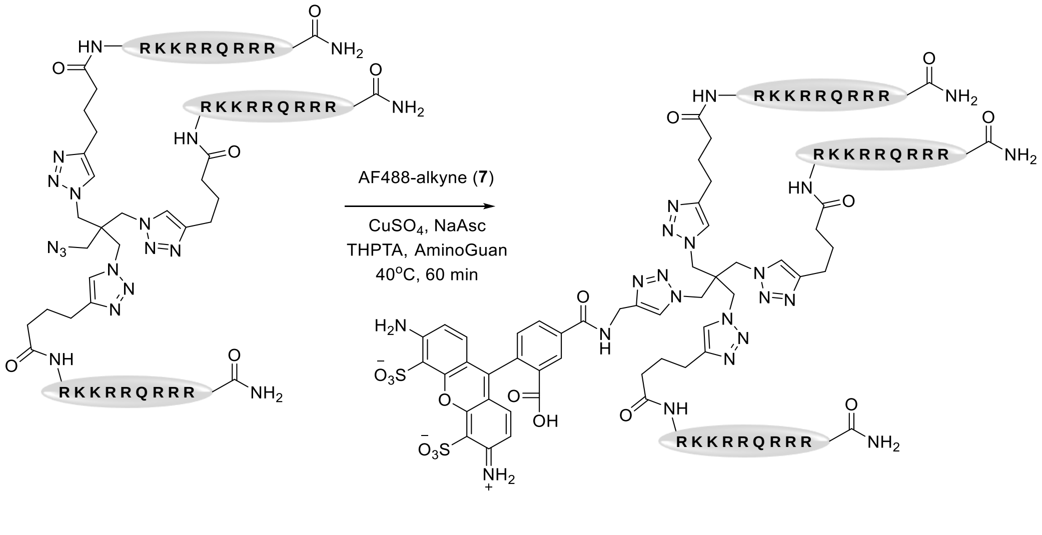

5
$C_{182}H_{347}N_{105}O_{33}$
(4534.48)

8
$C_{206}H_{364}N_{108}O_{43}S_2$
(5106.00)

tri-Tat A (8)

Tat_3-N_3 (**5**) (175 μg, 0.038 μmol, 1 eq.), AlexaFluor™488 5-carboxyamido(propargyl), bis(triethylammonium) salt, 5-isomer (**7** – AF488-alkyne) (Thermo Fisher Scientific, USA) (60 μg, 0.08 μmol, 2 eq.), as well as CuAAC regents were dissolved in 30 % DMSO / PBS and heated at 40°C for 60 min. The product was purified by HPLC, (33 μg, 0.007 μmol, 18 % yield).

HPLC retention time r_t = 18:04 min; MS QTOF [ES+]: 5106.37 (calculated: 5106.00 for $C_{206}H_{364}N_{108}O_{43}S_2$)

(a)

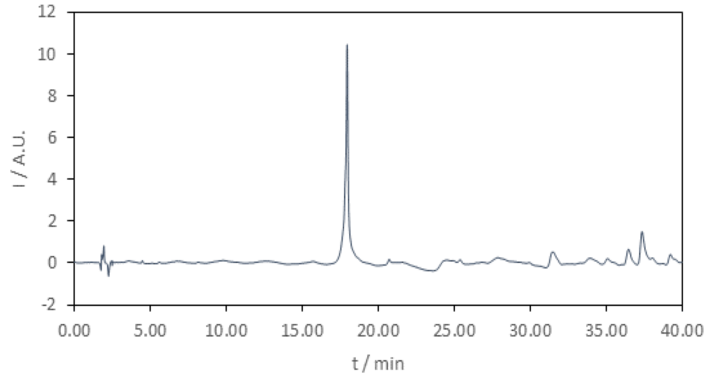

(b)

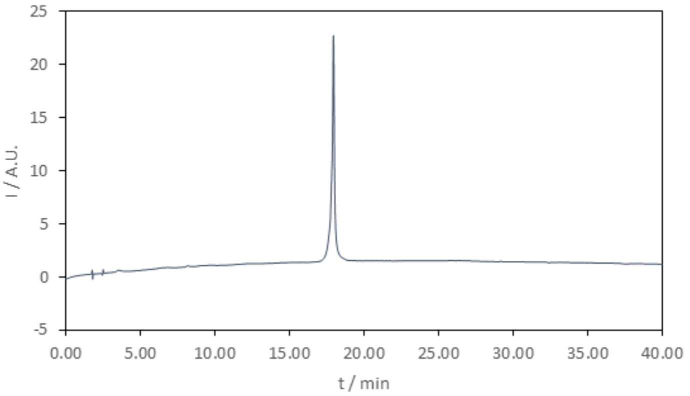

HPLC trace of **tri-Tat A** (**8**) at 250 nm (**a**) and 490 nm (**b**)

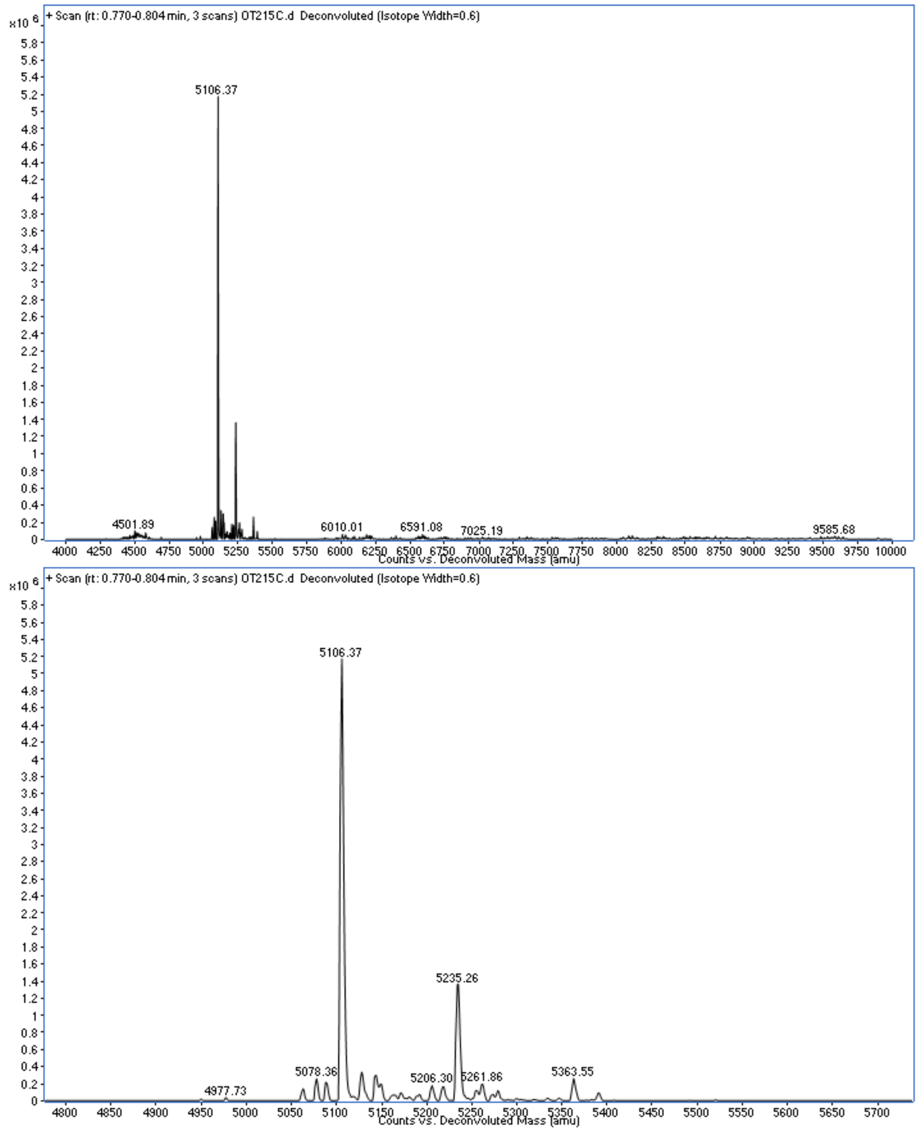

Deconvoluted MS QTOF mass spectra of **tri-Tat A** (**8**)

S2.6. Compound 9

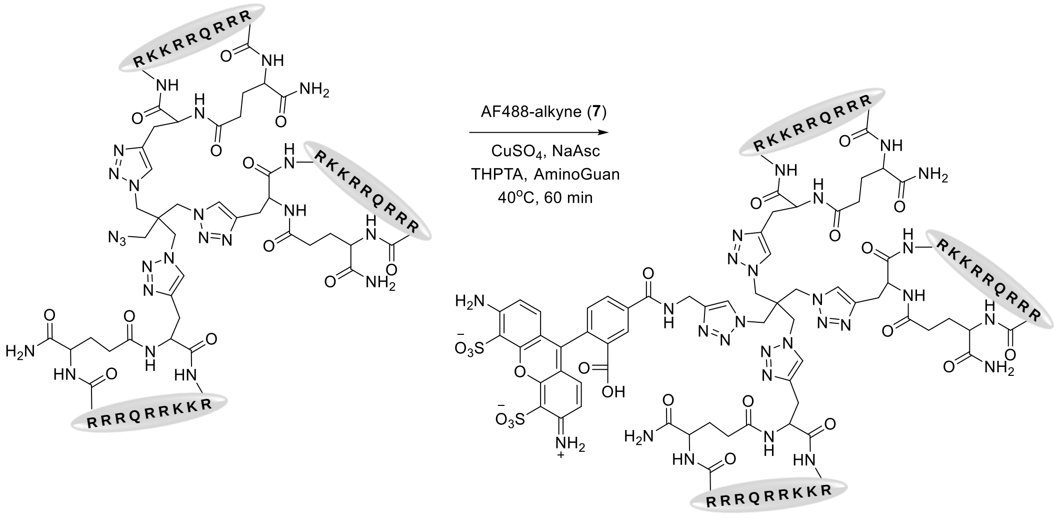

6
$C_{194}H_{359}N_{111}O_{39}$
(4870.74)

9
$C_{218}H_{376}N_{114}O_{49}S_2$
(5442.28)

tri-cTat A (9)

cTat3-N3 (**6**) (100 μg, 0.02 μmol, 1 eq.), AlexaFluor™488 5-carboxyamido(propargyl), bis(triethylammonium) salt, 5-isomer (**7** – AF488-alkyne) (Thermo Fisher Scientific, USA) (31 μg, 0.04 μmol, 2 eq.), as well as CuAAC regents were dissolved in 30 % DMSO / PBS and heated at 40°C for 60 min. The product was purified by HPLC, (35 μg, 0.006 μmol, 32 % yield). HPLC retention time r_t = 18:47 min; MS QTOF [ES+]: 5443.27 (calculated: 5442.28 for $C_{218}H_{376}N_{114}O_{49}S_2$).

(a)

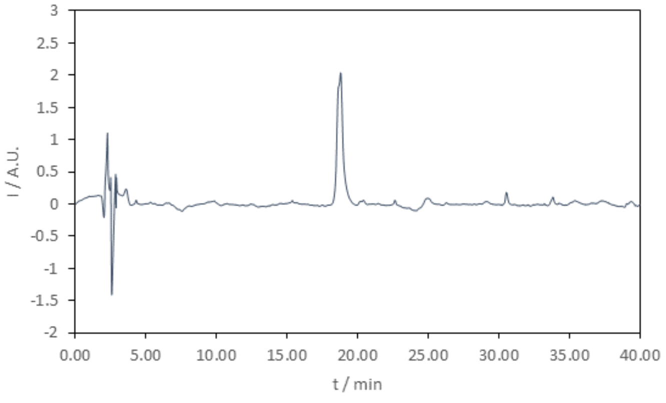

(b)

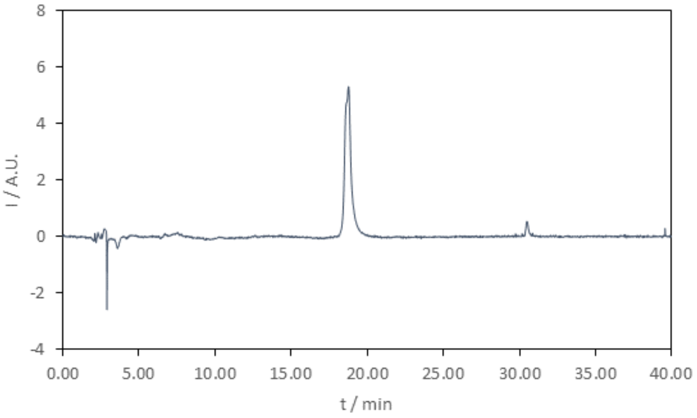

HPLC trace of **tri-cTat A** (**9**) at 250 nm (**a**) and 490 nm (**b**)

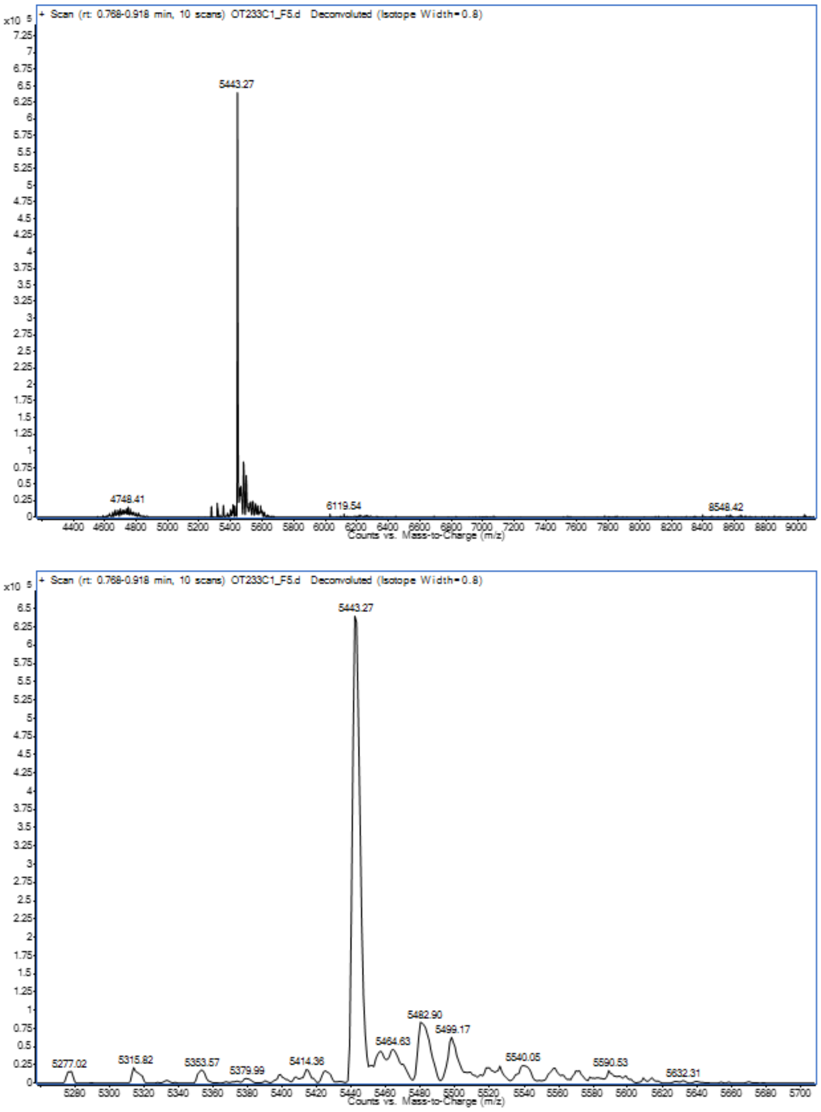

Maximum entropy deconvoluted MS QTOF mass spectra of **tri-cTat A (9)**

S2.7. Compound 13

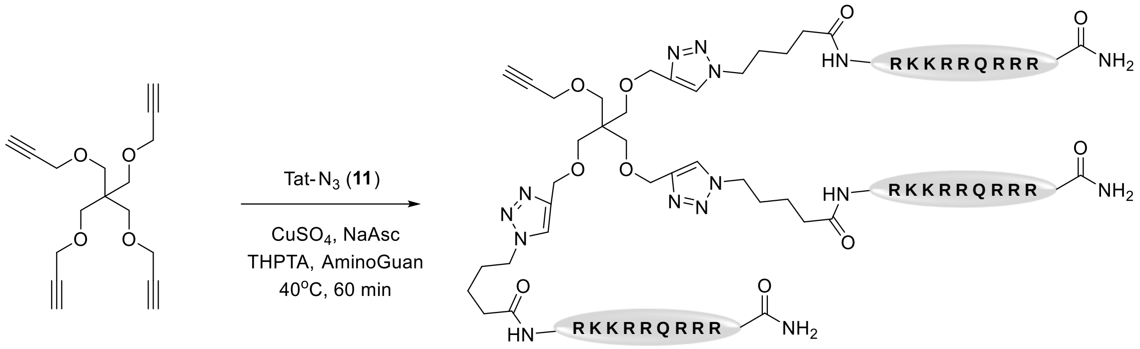

10
$C_{17}H_{20}O_4$
(288.34)

13
$C_{191}H_{365}N_{102}O_{37}$
(4682.67)

Tat3-alkyne (13)

Tetrakis(2-propynyloxymethyl)methane (**10**) (13.4 μg, 0.05 μmol, 1 eq.), and 5-azido-pentanoyl-Tat-NH_2 (**11** - Tat-azide (49-57)) (Peptide International Inc., USA) (410 μg, 0.28 μmol, 6 eq.) as well as CuAAC regents were dissolved in 30 % DMSO / PBS and heated at 40°C for 60 min. The product was purified by HPLC (135 μg, 0.029 μmol, 61 % yield).

HPLC retention time r_t = 20:13 min; MS QTOF [ES+]: 4683.71 (calculated: 4682.67 for $C_{191}H_{365}N_{102}O_{37}$)

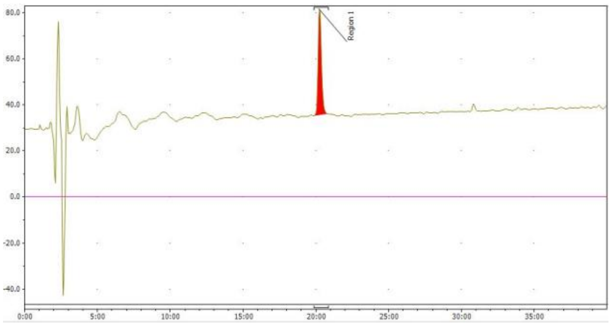

HPLC trace of **Tat3-alkyne** (**13**) at 250 nm

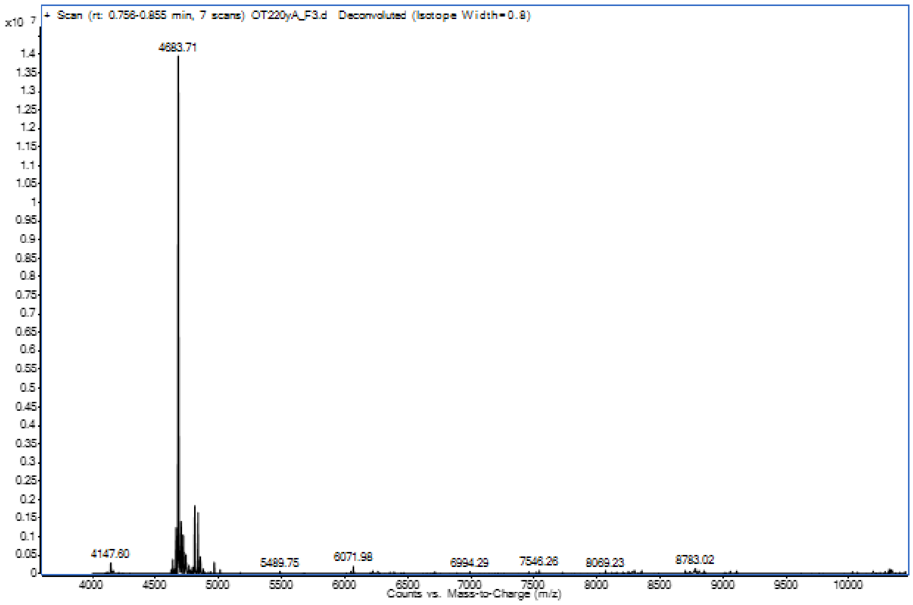

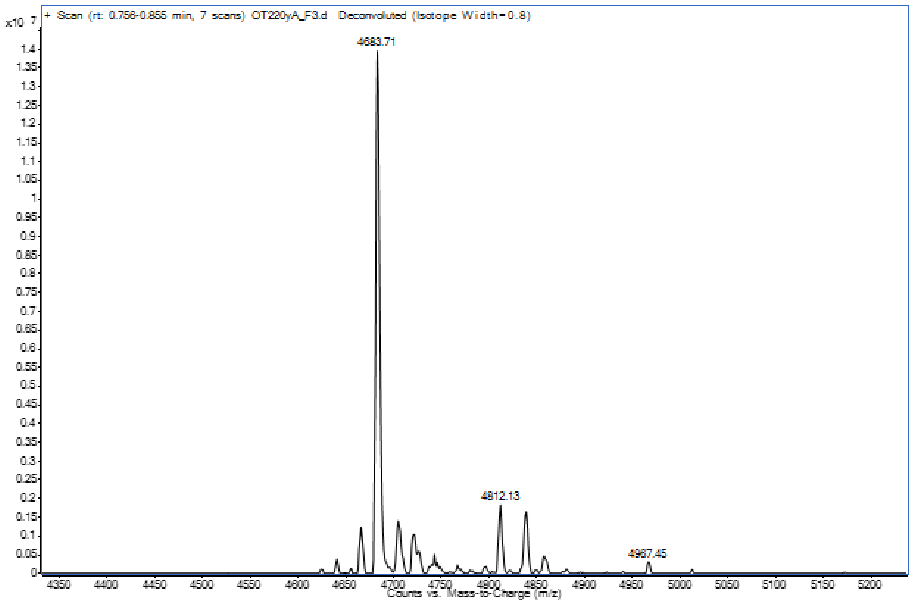

Maximum entropy deconvoluted MS QTOF mass spectra of **Tat$_3$-alkyne (13)**

S2.8. Compound 14

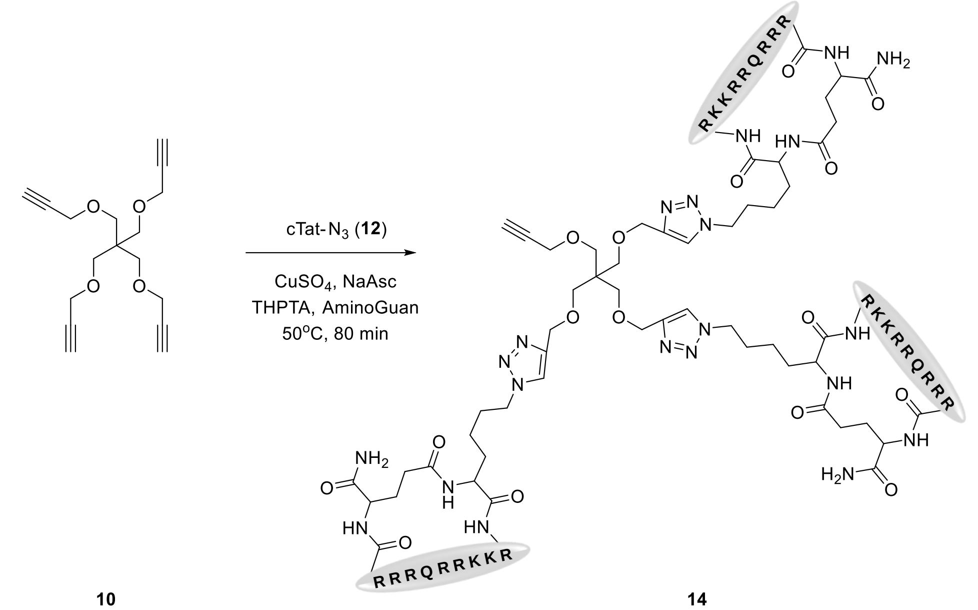

10
$C_{17}H_{20}O_4$
(288.34)

14
$C_{209}H_{386}N_{108}O_{43}$
(5100.10)

cTat$_3$-alkyne (14)

Tetrakis(2-propynyloxymethyl)methane (**10**) (28.3 μg, 0.10 μmol, 1 eq.), and azidolysine-cyclicTat-NH_2 (**12**, cTat-azide (49-57)) (Cambridge Peptide, UK) (675 μg, 0.42 μmol, 4.2 eq.) as well as CuAAC regents were dissolved in 30 % DMSO / PBS and heated at 50°C for 80 min. The product was purified by HPLC (255 μg, 0.05 μmol, 51 % yield).

HPLC retention time r_t = 20:12 min; MS QTOF [ES+]: 5100.32 (MW calculated: 5100.10 for $C_{209}H_{386}N_{108}O_{43}$)

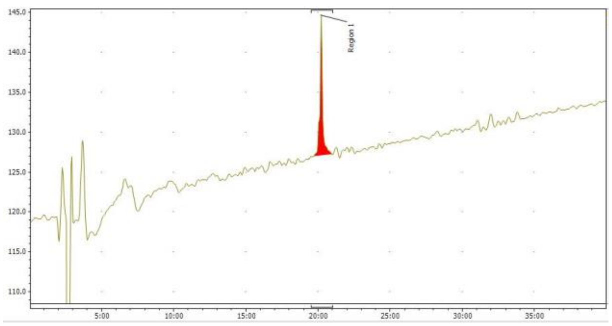

HPLC trace of **cTat$_3$-alkyne** (**14**) at 250 nm

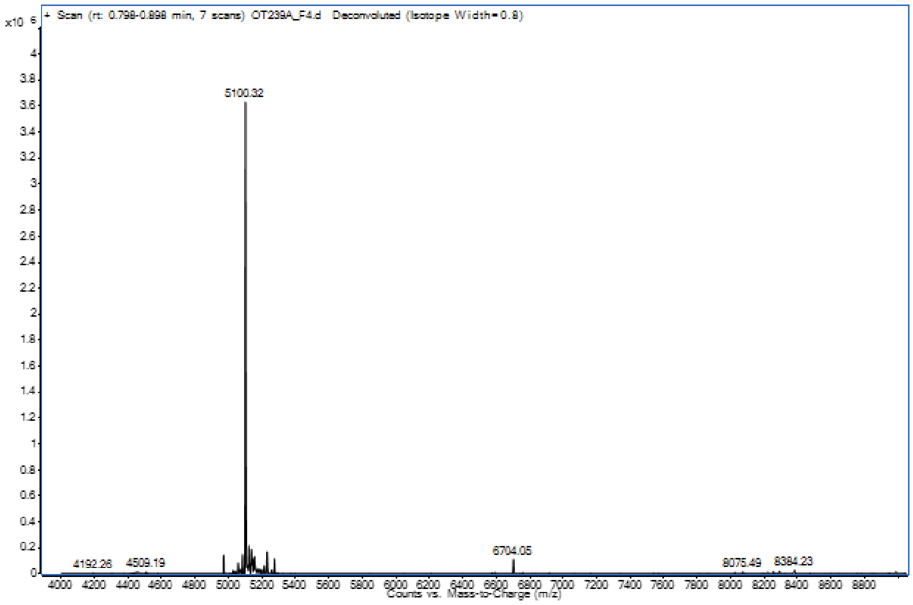

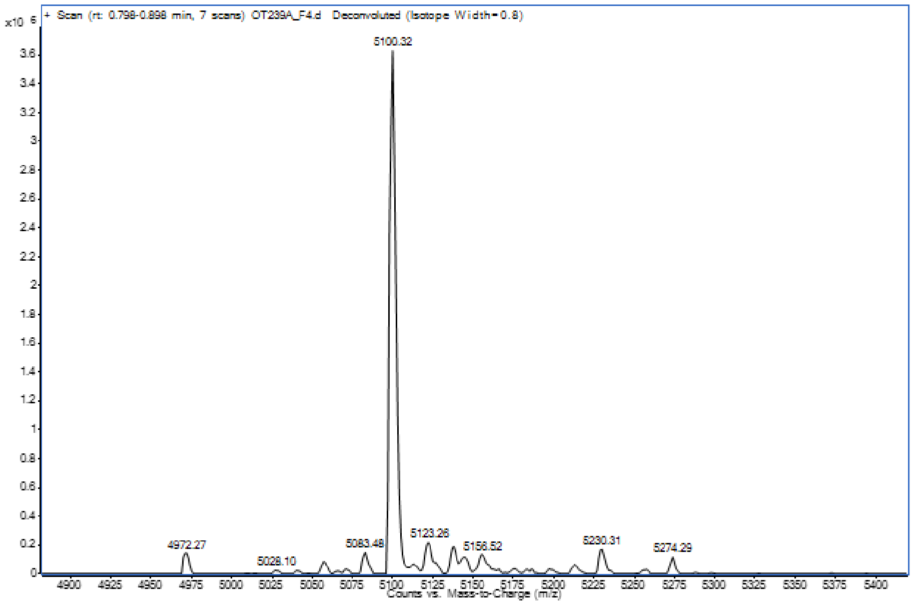

Maximum entropy deconvoluted MS QTOF mass spectra of **cTat$_3$-alkyne (14)**

S2.9. Compound 16

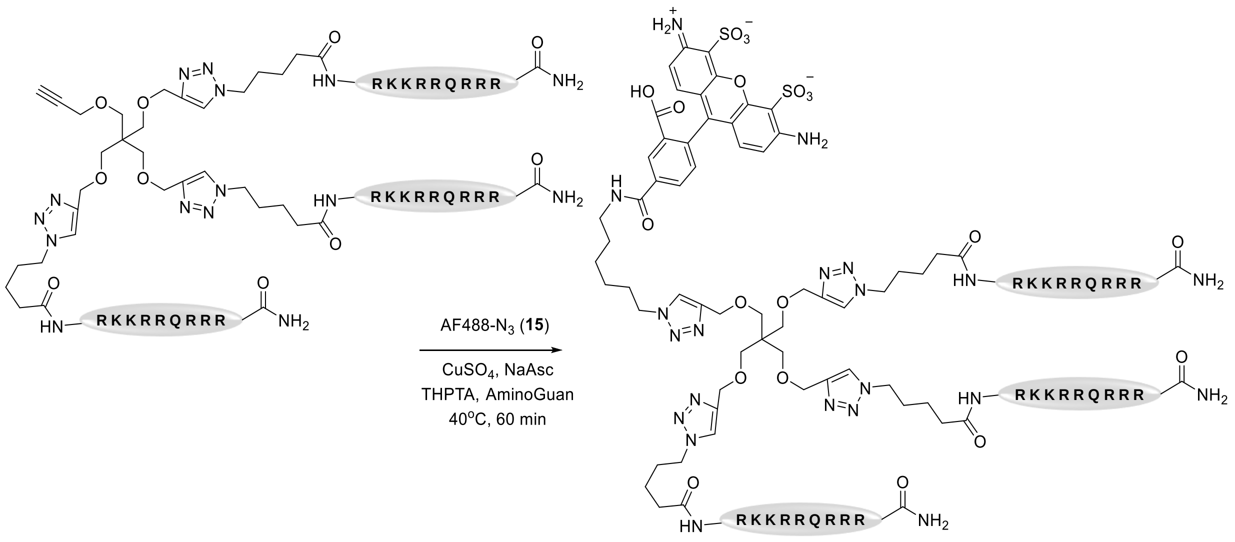

13
$C_{191}H_{362}N_{102}O_{37}$
(4679.67)

16
$C_{218}H_{391}N_{108}O_{47}S_2$
(5341.33)

tri-Tat B (16)

Tat3-alkyne (**13**) (135 μg, 0.029 μmol, 1 eq.), Alexa Fluor™ 488 5-Carboxamido-(6-Azidohexanyl), Bis(Triethylammonium Salt), 5-isomer (**15** – AF488-azide) (Thermo Fisher Scientific, USA) (45 μg, 0.06 μmol, 2 eq.), as well as CuAAC regents were dissolved in 30 % DMSO / PBS and heated at 40°C for 60 min. The product was purified by HPLC (55 μg, 0.01 μmol, 36 % yield).

HPLC retention time r_t = 22:43 min; MS QTOF [ES+]: 5342.18 (calculated: 5341.33 for $C_{218}H_{391}N_{108}O_{47}S_2$).

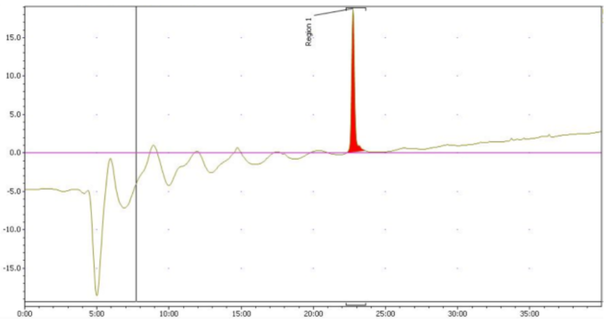

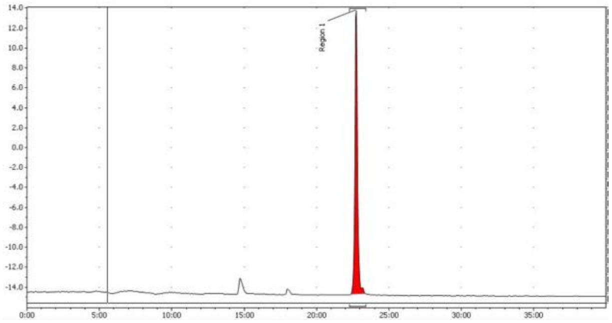

HPLC trace of **tri-Tat B** (**16**) at 250 nm (**a**) and 490 nm (**b**)

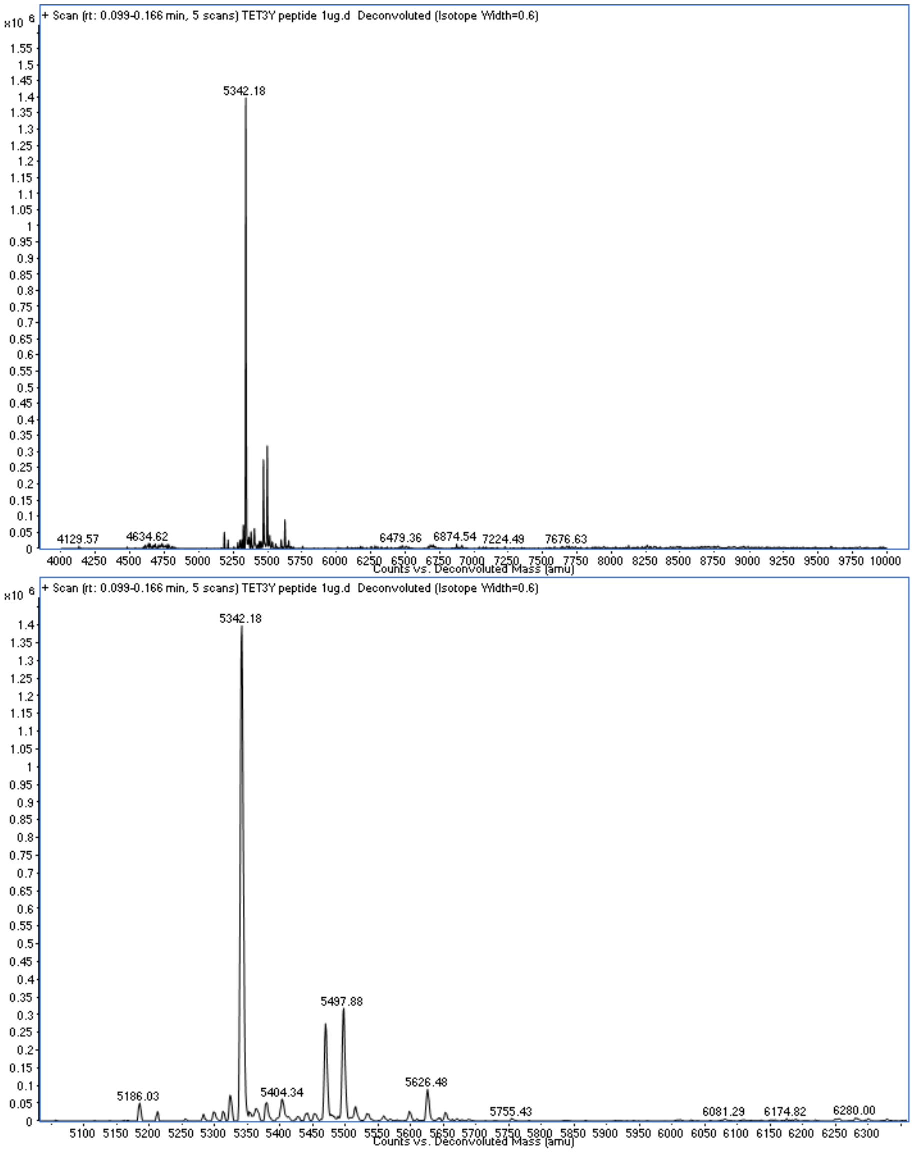

Maximum entropy deconvoluted MS QTOF mass spectra of **tri-Tat B** (**16**)

S2.10. Compound 17

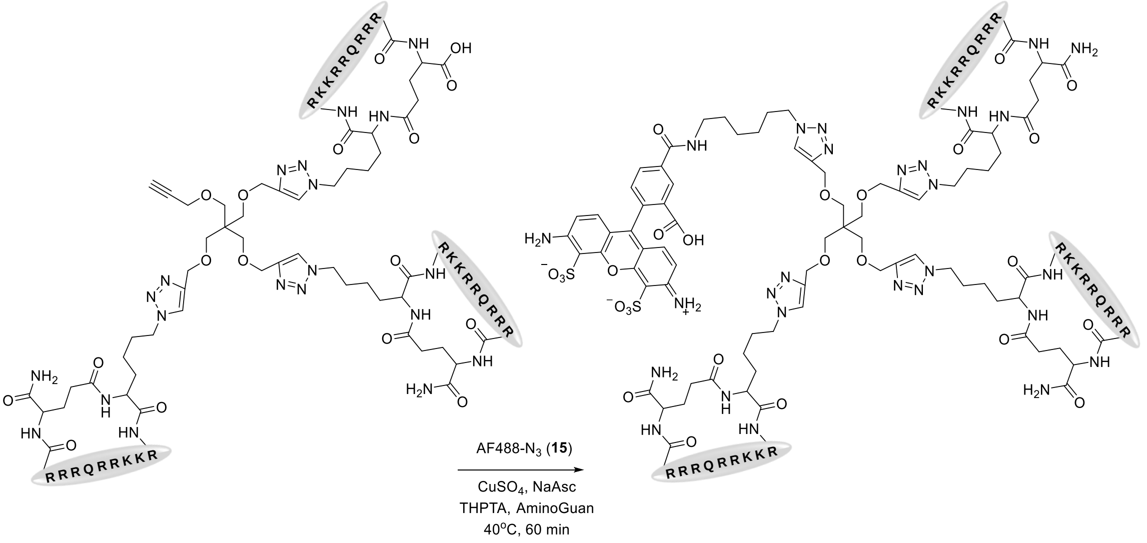

14
$C_{209}H_{386}N_{108}O_{43}$
(5100.10)

17
$C_{236}H_{412}N_{114}O_{53}S_2$
(5758.76)

tri-cTat B (17)

cTat3-alkyne (**14**) (255 μg, 0.05 μmol, 1 eq.), Alexa Fluor™ 488 5-Carboxamido-(6-Azidohexanyl), Bis(Triethylammonium Salt), 5-isomer (**15** – AF488-azide) (Thermo Fisher Scientific, USA) (75 μg, 0.10 μmol, 2 eq.), as well as CuAAC reagents were dissolved in 30 % DMSO / PBS and heated at 40°C for 60 min. The product was purified by HPLC (130 μg, 0.022 μmol, 45 % yield).

HPLC retention time r_t = 21:07 min; MS QTOF [ES+]: 5759.09 (MW calculated: 5758.76 for $C_{236}H_{412}N_{114}O_{53}S_2$).

(a)

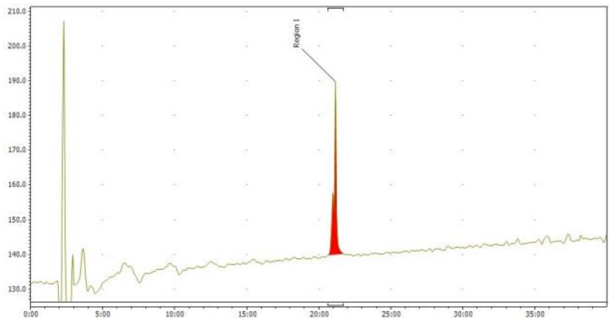

(b)

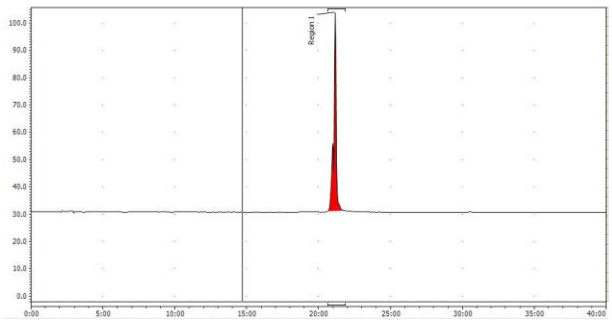

HPLC trace of **tri-cTat B** (**17**) at 250 nm (**a**) and 490 nm (**b**)

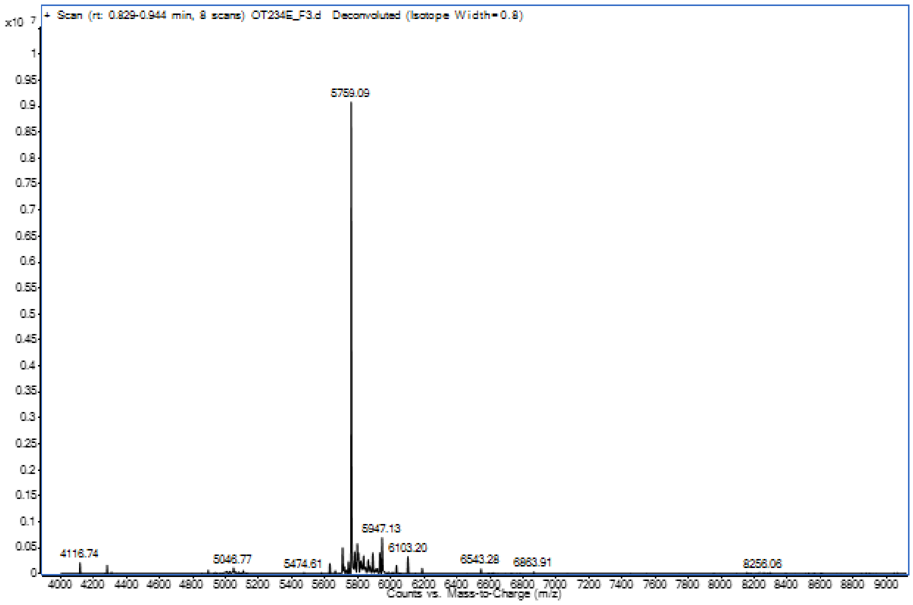

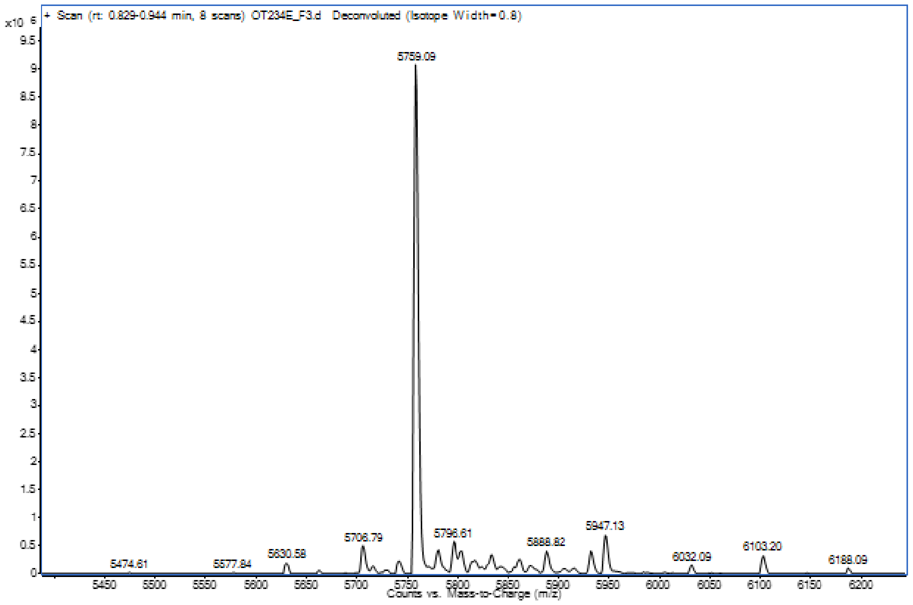

Maximum entropy deconvoluted MS QTOF mass spectra of **tri-cTat B (17)**

S2.11. Compound 18

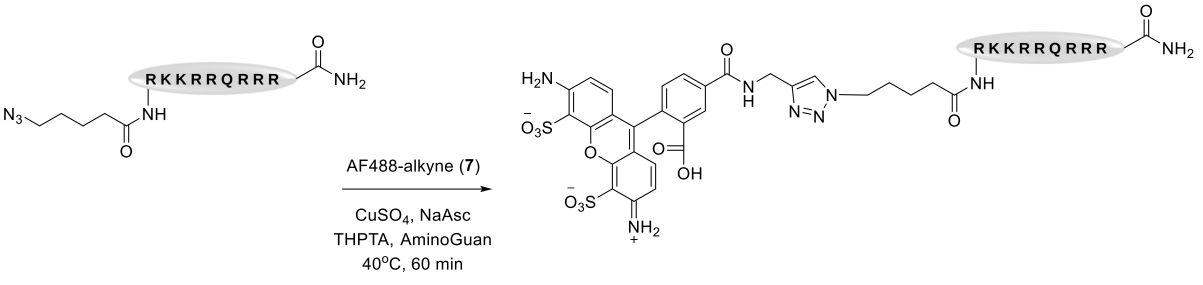

11
$C_{58}H_{114}N_{34}O_{11}$
(1463.78)

18
$C_{82}H_{133}N_{37}O_{21}S_2$
(2035.98)

mono-Tat (18)

5-azido-pentanoyl-Tat-NH_2 (**3** - Tat-azide (49-57)) (Peptide International Inc., USA) (205 μg, 0.14 μmol, 1 eq.), AlexaFluor^{TM}488 5-carboxyamido(propargyl), bis(triethylammonium) salt, 5-isomer (**5** – AF488-alkyne) (Thermo Fisher Scientific, USA) (162 μg, 0.21 μmol, 1.5 eq.), as well as CuAAC regents were dissolved in 30 % DMSO / PBS and heated at 40°C for 60 min. The product was purified by HPLC, (26 μg, 0.014 μmol, 10 % yield).

HPLC retention time r_t = 18:01 min; MS QTOF: 2035.94 (calculated: 2035.98 for $C_{82}H_{133}N_{37}O_{21}S_2$).

(a)

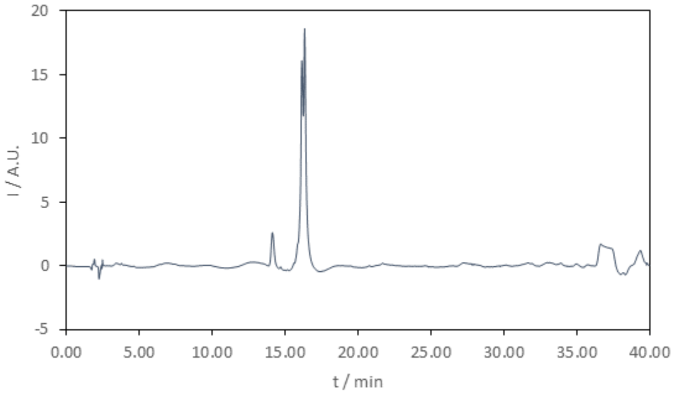

(b)

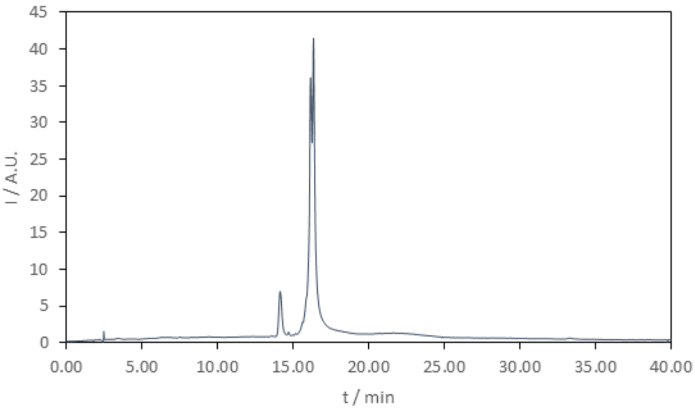

HPLC trace of **mono-Tat** (**18**) at 250 nm (**a**) and 490 nm (**b**)

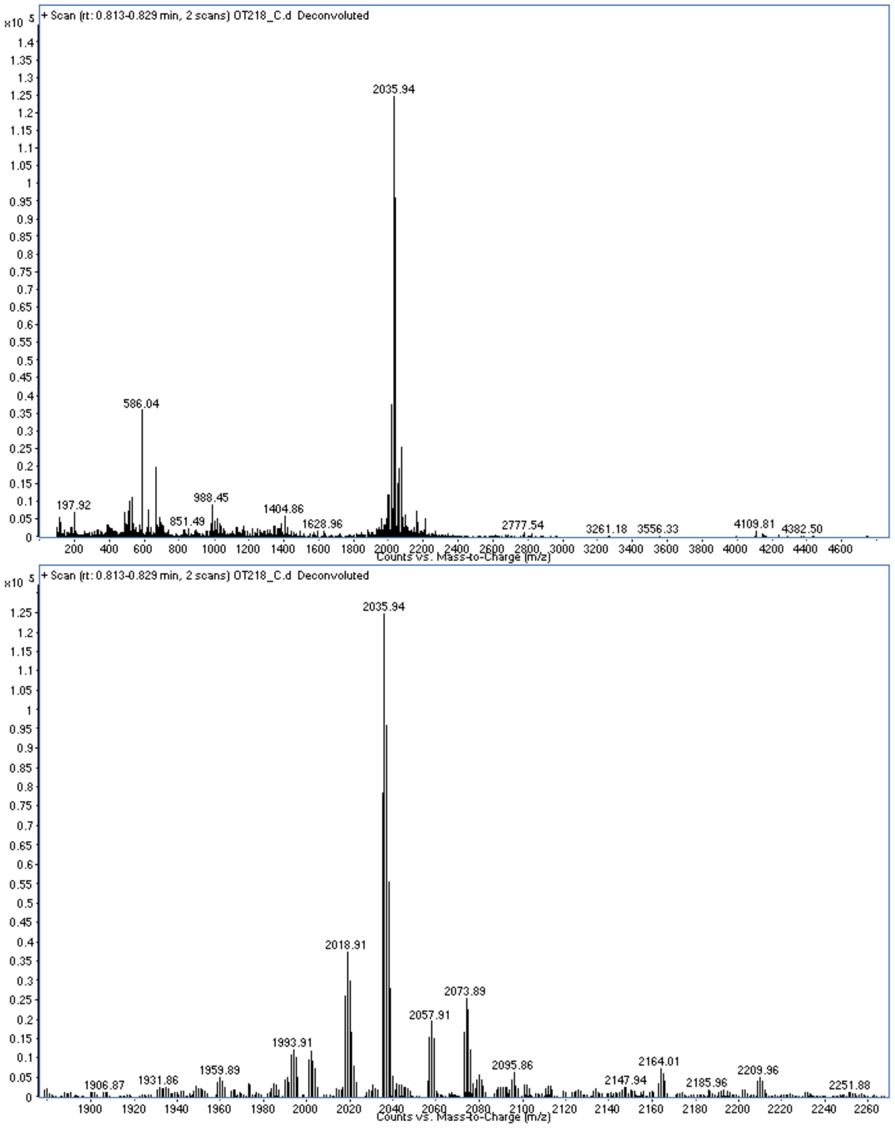

Resolved isotope deconvoluted MS QTOF mass spectra of **mono-Tat** (**18**)

S2.12. Compound 19

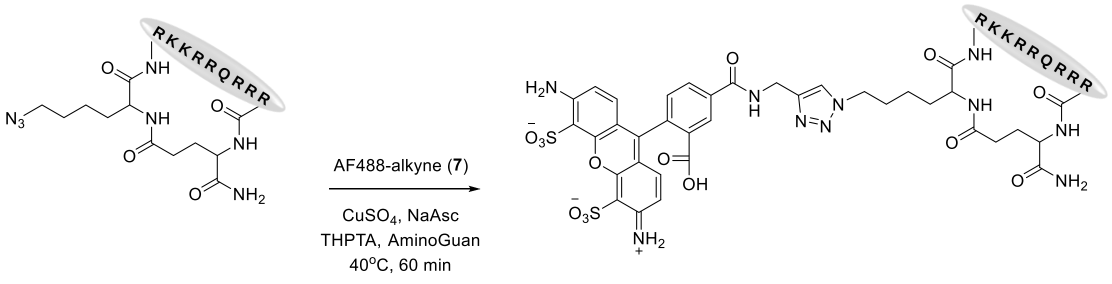

12
$C_{64}H_{122}N_{36}O_{13}$
(1603.91)

19
$C_{88}H_{139}N_{39}O_{23}S_2$
(2175.45)

mono-cTat (19)

Azidolysine-cyclicTat-NH2 (**12 -** cTat-azide (49-57)) (Cambridge Peptide, UK) (225 μg, 0.14 μmol, 1 eq.), AlexaFluor^{TM}488 5-carboxyamido(propargyl), bis(triethylammonium) salt, 5-isomer (**5** – AF488-alkyne) (Thermo Fisher Scientific, USA) (162 μg, 0.21 μmol, 1.5 eq.), as well as CuAAC regents were dissolved in 30 % DMSO / PBS and heated at 40°C for 60 min. The product was purified by HPLC (75 μg, 0.034 μmol, 24 % yield).

HPLC retention time r_t = 14:44 min; MS QTOF: 2075.03 (calculated: 2075.45 for $C_{88}H_{139}N_{39}O_{23}S_2$)

(a)

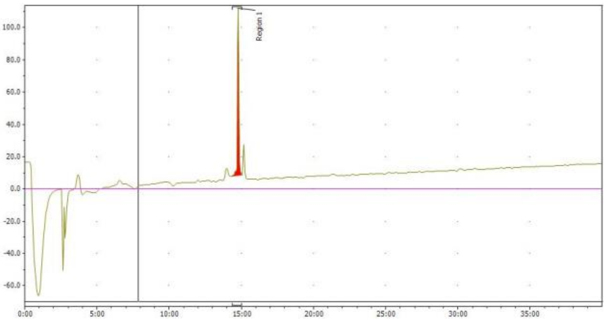

(b)

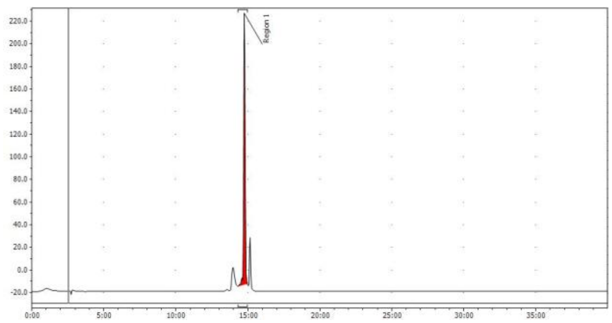

HPLC trace of **mono-cTat** (**19**) at 250 nm (**a**) and 490 nm (**b**)

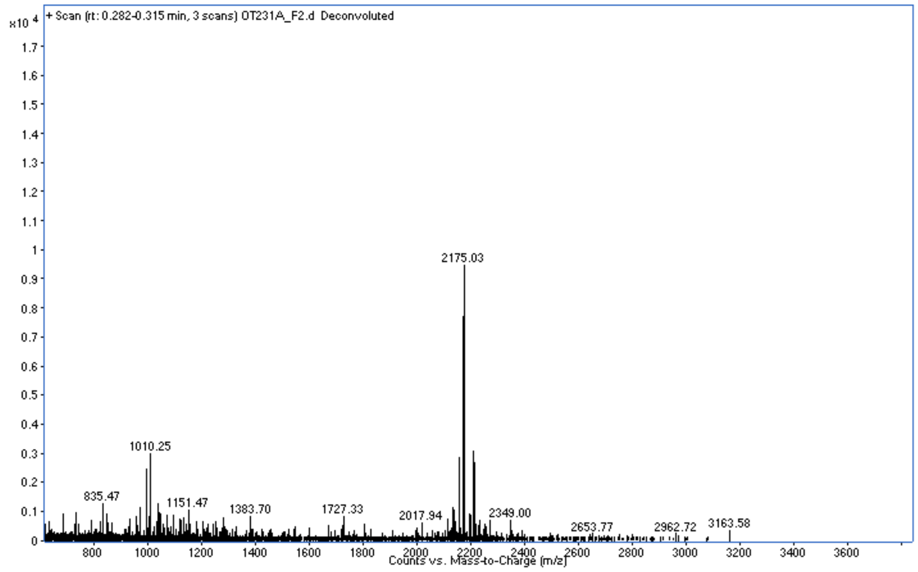

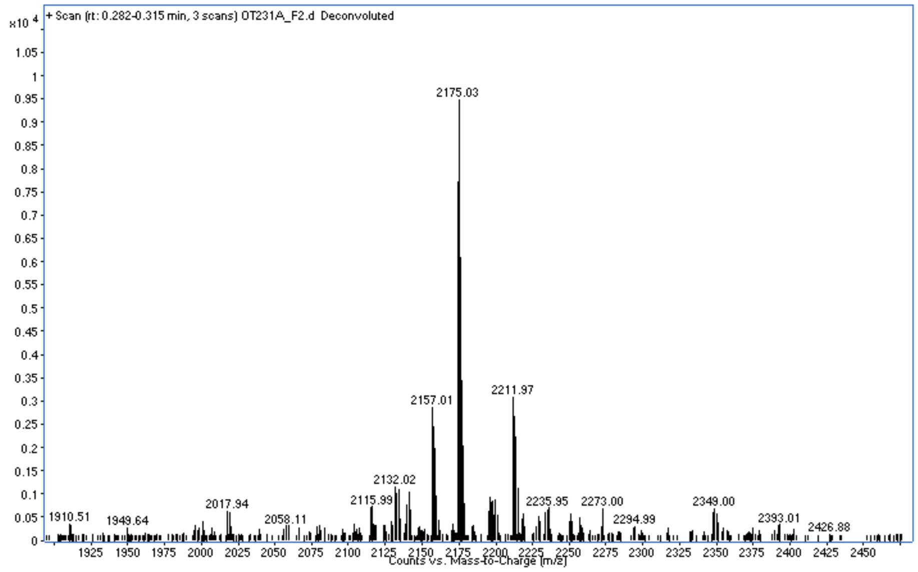

Resolved isotope deconvoluted MS QTOF mass spectra of **mono-cTat** (**19**)

S3. Photophysical properties of Tat conjugates

Tat-conjugates were diluted in PBS (pH 7.4) and UV absorbance, as well as fluorescence excitation / emission spectra acquired by plate reader.

S3.1. UV absorbance spectra

UV absorbance spectra were acquired from 400 nm to 600 nm, at 5 nm intervals.

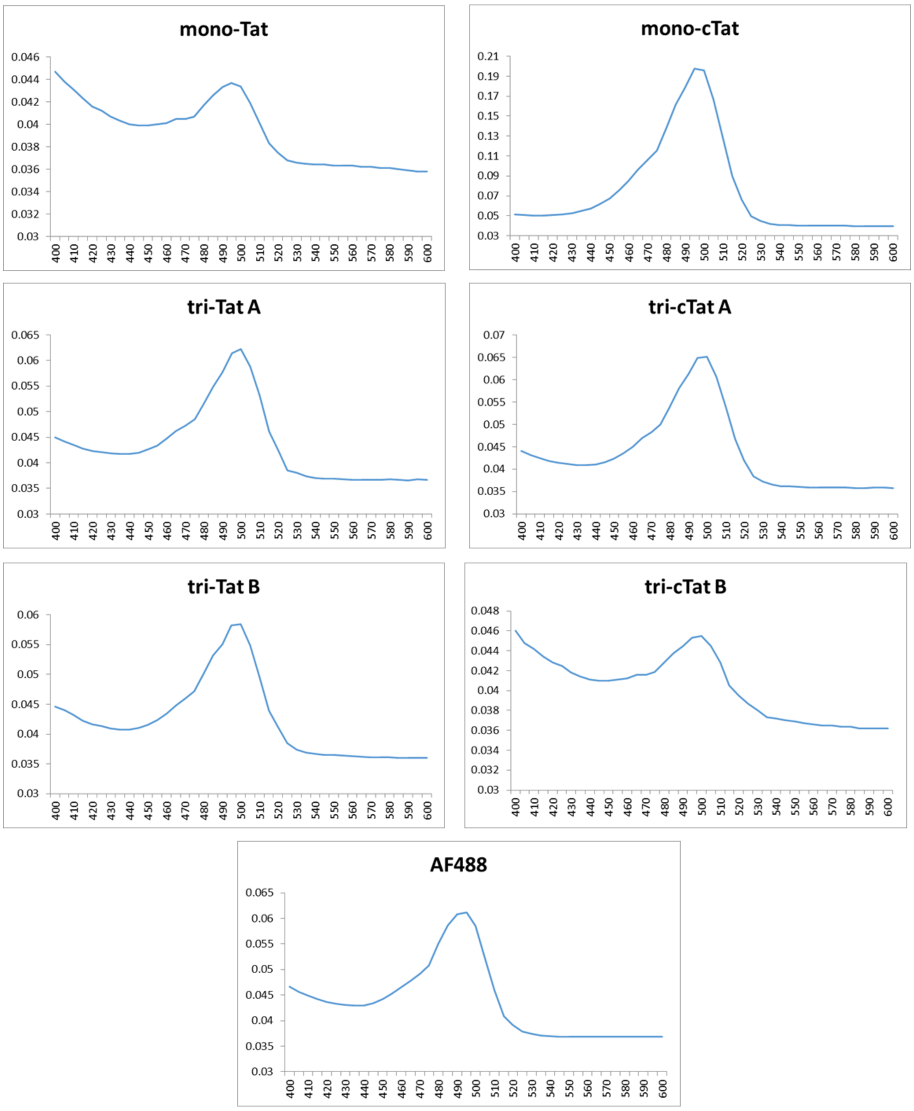

Figure 1: UV absorbance spectra of monomers, trimers and AF488 alone

S3.2. Fluorescence excitation / emission spectra

Excitation spectra were acquired by exciting sample between 400 nm and 560 nm at 5 nm intervals and measuring emission at 600 nm. Emission spectra were acquired by exciting sample at 460 nm and measuring emission between 500 nm and 650 nm at 5 nm intervals.

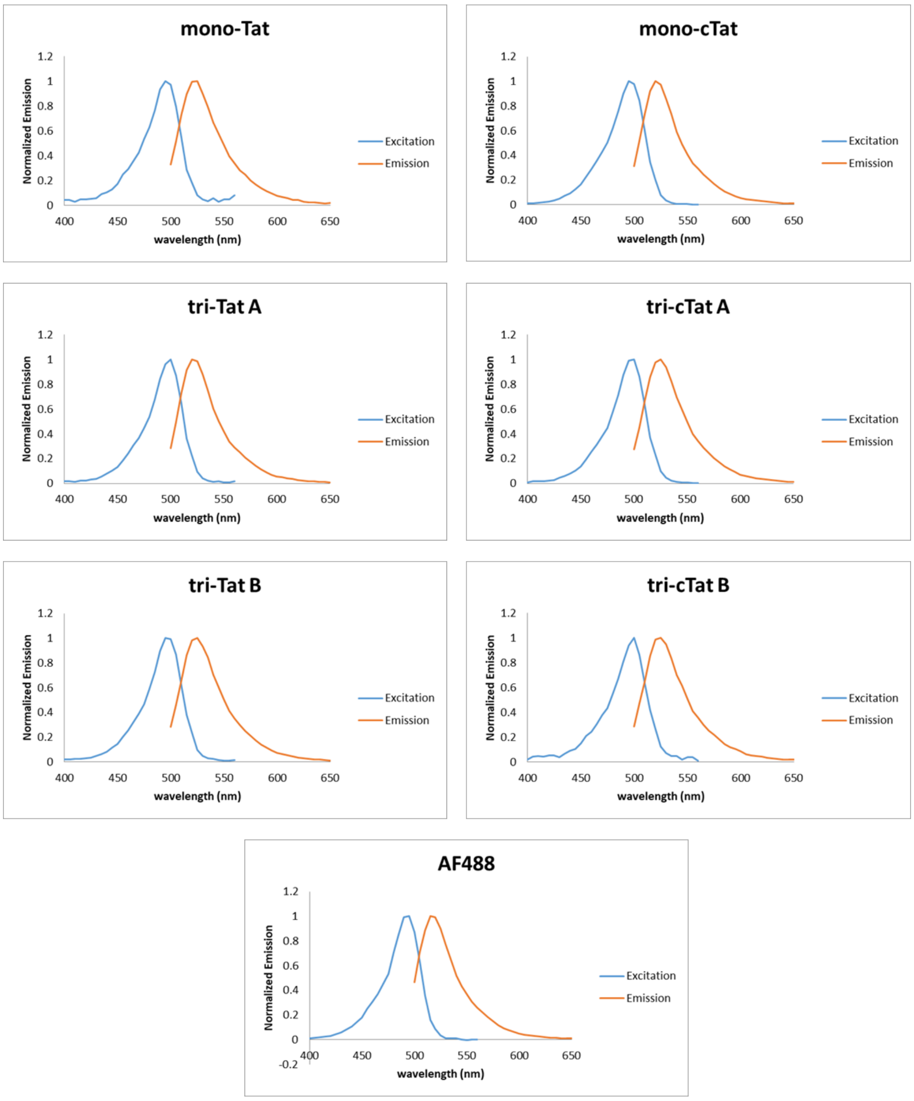

Figure 2: Fluorescence excitation / emission spectra of monomers, trimers, and AF488 alone

S4. Cell culture / preparation

HeLa and CHO cells were cultured in DMEM supplemented with 10% FBS and penicillin / streptomycin. Cell lines were maintained at 37 °C in an atmosphere of 5% CO_2 and sub-cultured using Trypsin. Cell lines were used at passage numbers 20 or lower and checked to be mycoplasma-free on a monthly basis. Stock solutions of Tat peptides were prepared in PBS before dilution in cell media.

S5. Live cell confocal microscopy

Cells were seeded on greiner 75/25 mm glass bottom cellview cell culture slides (Greiner Bio-One), allowed to adhere for 24 h and washed with serum free medium (x3) in preparation for the experiment. For post-wash experiments, Tat conjugate was added to the cells in serum free medium, cells incubated at 37 °C in an atmosphere of 5% CO_2, culture medium removed at the desired time point, cells washed with serum supplemented medium (x3) and cells transferred to the microscope for imaging (microscope incubation chamber at 37 °C, 5% CO_2). For time course or pre-wash experiments, cells were transferred to the microscope (incubation chamber at 37 °C, 5% CO_2) and left to acclimatize for 15 min; thereafter Tat conjugate was added directly to cell medium and cells imaged at desired time points. Samples were visualized using a Zeiss LSM 780 inverted confocal microscope fitted with a XLmultiS1 incubation chamber and Plan-Apochromat 63x / 1.40 Oil DIC M27 objective. AlexFluor488 (λex = 488 nm) and propidium iodide / red fluorescence protein (RFP) (λex = 543 nm) or AlexaFluor647 (λex = 633 nm) were collected at 493-551 nm and 548-669 nm or 493-551 nm and 650 – 748 nm respectively. Images were collected sequentially to avoid spectral overlap between dyes and acquired at laser strength of 1% with a gain of 600 (green channel) and 800 (red channels). Bright field images were collected using a transmission detector and the 458 laser lines. Four channel images (Figure 5) were

acquired sequentially using the 458 nm (Brightfield), 488 nm (AlexaFluor488), 543 nm (RFP), and 633 (AlexaFluor647) laser lines with 493 – 551 nm (AF488), 568 – 604 nm (RFP), and 650 – 748 nm (AF647) filter settings (Laser Strength and Gain as stated above). Images were analyzed using ImageJ software. Digital adjustments and image processing are consistent throughout the manuscript, unless otherwise stated in the figure legend. Statistical analysis was carried out using GraphPad Prism 7 software and all data used for statistical analysis consists of measurements from distinct cells. Where applicable a non-parametric, unpaired, two-tailed (Mann-Whitney) test was used for statistical testing. The transduction efficiency data presented in Figures 2e, 2j, 5d and Supplementary Figure 15 were generated by randomly selecting multiple images from at least two separate experiments and counting the total number of cells in the image as well as the number of transduced cells. Transduced cells were scored as positive when they showed homogenous cytoplasmic and nucleolar fluorescence due to the presence of trimer (Figures 2e, 2j, and Supplementary Figure 9) or cargo (Fig. 5d). The ratio of transduced cells:total number of cells was calculated for each image with mean and standard deviation of ratios shown in graphs. As such, the standard deviation represents the variation in transduction efficiency between different groups of cells

S6. Supplementary live cell confocal microscopy images

S6.1. mono-Tat (18) / mono-cTat (19) (10 μM) in HeLa and CHO cells

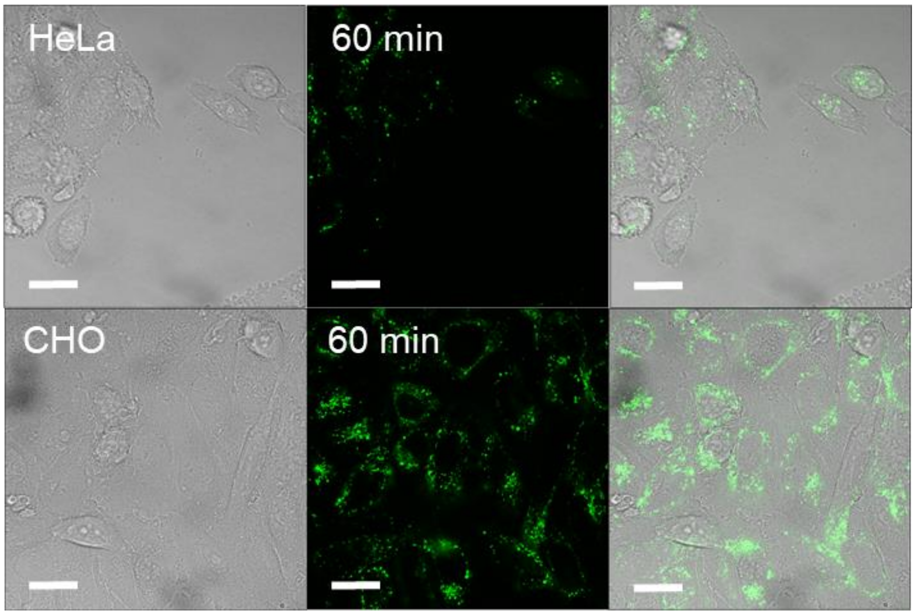

Figure 3a: **Live-cell confocal microscopy of live HeLa and CHO cells treated with 10 μM mono-Tat** (**18**) for 60 min incubation; imaging post-wash. 10 μM **mono-Tat** is taken up only into endosomes of HeLa and CHO cells. Scale bar: 20 μm

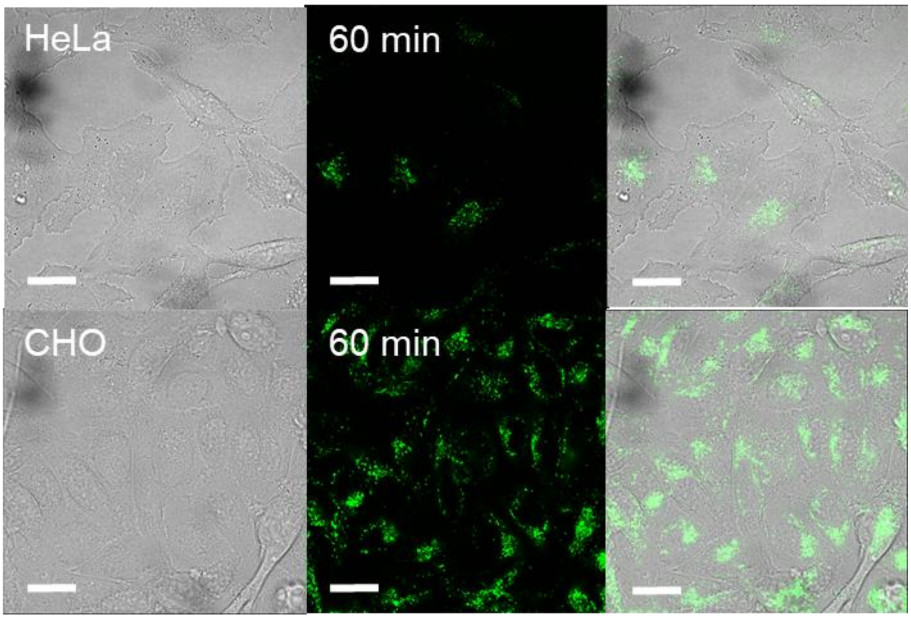

Figure 3b: Live-cell confocal microscopy of live HeLa and CHO cells treated with 10 μM mono-cTat (**19**) for 60 min incubation; imaging post-wash. 10 μM **mono-cTat** is taken up only into endosomes of HeLa and CHO cells. Scale bar: 20 μm.

S6.2. Co-localization of tri-Tat A (8), tri-Tat B (16) and tri-cTat B (17) with rab5a-RFP as an early endosome marker

HeLa cells were transfected with rab5a-RFP, a marker of early endosomes linked to red fluorescence protein for detection, using a CellLight^{TM} early endosomes-RFP, BacMam 2.0 kit (Thermo Fisher Scientific, USA) according to manufacturer's instructions. Briefly, cells were seeded on greiner 75/25 mm glass bottom cellview cell culture slides (Greiner Bio-One), allowed to adhere for 1 h, treated with 2 μL transfection reagent and left to adhere for a further 24 h. Cells were washed with serum-free medium (x3) in preparation for the experiment. Details of co-localization analysis can be found under section S8.3.

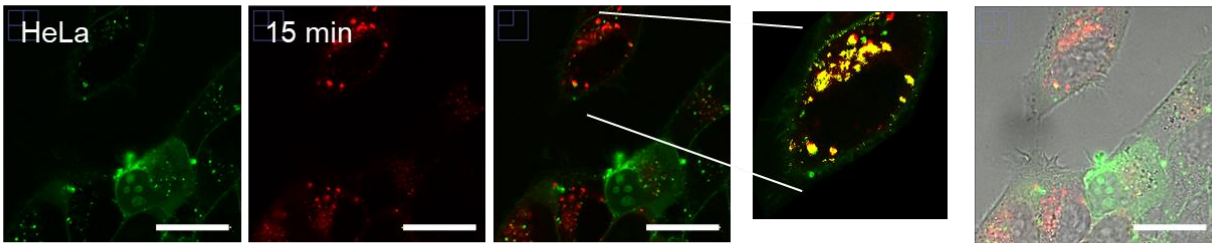

Figure 4a: **Live-cell confocal microscopy of live HeLa cells transfected with rab5a-RFP and treated with 1 μM tri-Tat A** (**8**) tagged with AF488 for 15 min; imaging pre-wash. Images from left to right represent – green channel, red channel, composite green / red channels, co-localization analysis (co-localized pixels in yellow), composite green / red / bright field channels. In cells transfected with rab5a-RFP, RFP and focal AF488 signal co-localize, indicating that the vesicles in which **tri-Tat A** is confined are early endosomes. Results of co-localization analysis: R = 0.264; M1 = 0.1878; M2 = 0.4714. Scale bar: 20 μm.

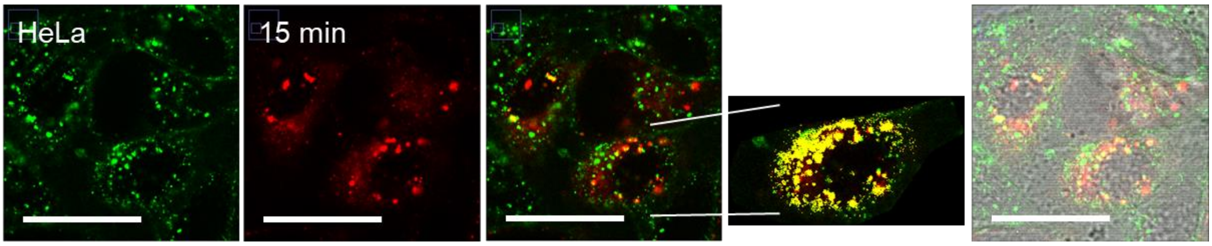

Figure 4b: **Live-cell confocal microscopy of live HeLa cells transfected with rab5a-RFP and treated with 1 μM tri-Tat B** (**16**) tagged with AF488 for 15 min; imaging pre-wash. Images from left to right represent – green channel, red channel, composite green / red channels, co-localization analysis (co-localized pixels in yellow), composite green / red / bright field channels. In cells transfected with rab5a-RFP, RFP and focal AF488 signal co-localizes, indicating that the vesicles in which **tri-Tat B** is confined are early endosomes. Results of co-localization analysis: R = 0.242; M1 = 0.5105; M2 = 0.5191. Scale bar: 20 μm.

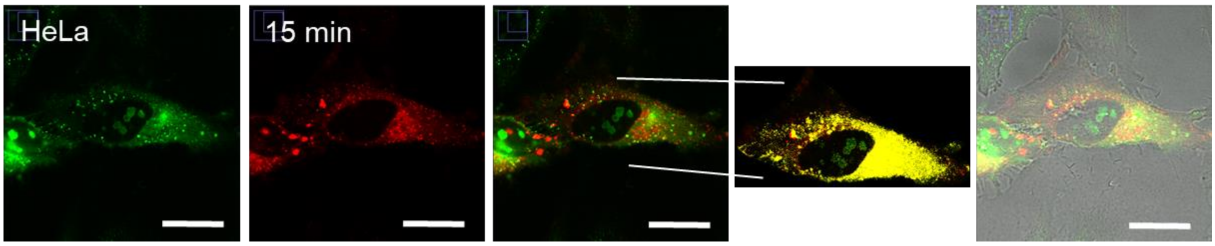

Figure 4c: Live-cell confocal microscopy of live HeLa cells transfected with rab5a-RFP and treated with 1 μM tri-cTat B (**17**) tagged with AF488 for 15 min; imaging pre-wash. Images from left to right represent – green channel, red channel, composite green / red channels, co-localization analysis (co-localized pixels in yellow), composite green / red / bright field channels. In cells transfected with rab5a-RFP, RFP and focal AF488 signal co-localizes, indicating that the vesicles in which **tri-cTat B** is confined are early endosomes. Results of co-localization analysis: R = 0.474; M1 = 0.7127; M2 = 0.6757. Scale bar: 20 μm.

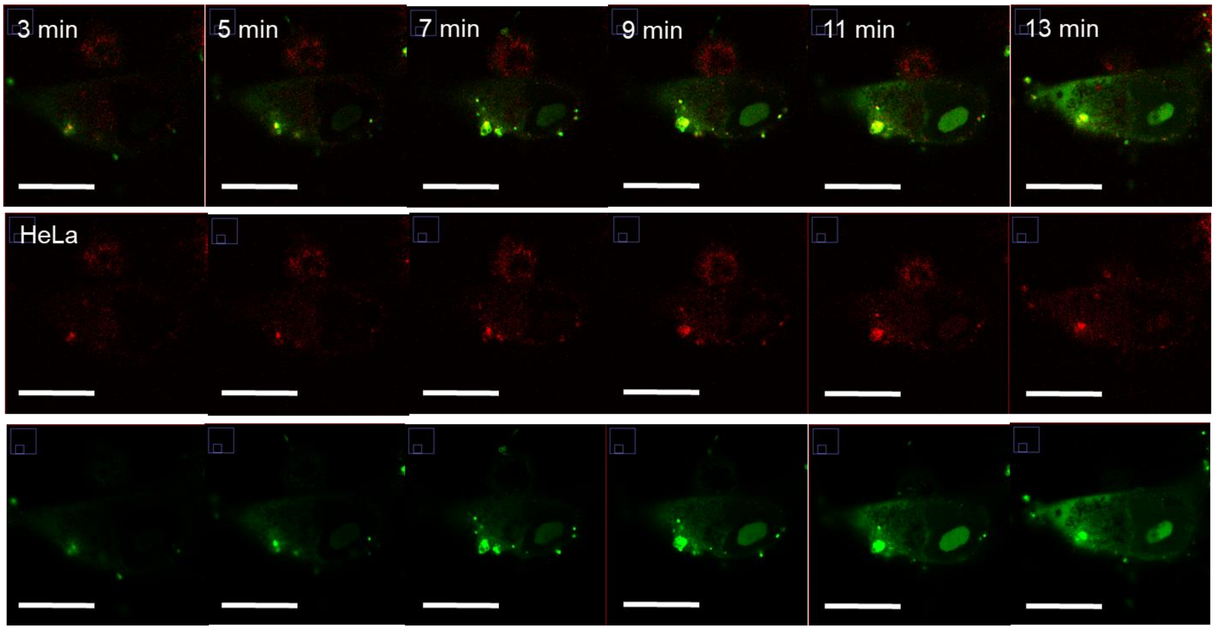

Figure 5: Time course of rab5a association with tri-cTat B vesicles. Live-cell confocal microscopy of live HeLa cells transfected with rab5a-RFP and treated with 1 μM **tri-cTat B** (**17**) tagged with AF488; imaging pre-wash. Images from left to right: 3 min, 5 min, 7 min, 9 min, 11 min, 13 min; top row - composite green / red channels; middle row – red channel; bottom row – green channel. rab5a-RFP associates with **tri-cTat B** vesicles before release of the trimer into the cytosol, suggesting that **tri-cTat B** escapes from the early endosome. Scale bar: 20 μm.

S6.3. Co-staining of tri-Tat A and PI in HeLa cells

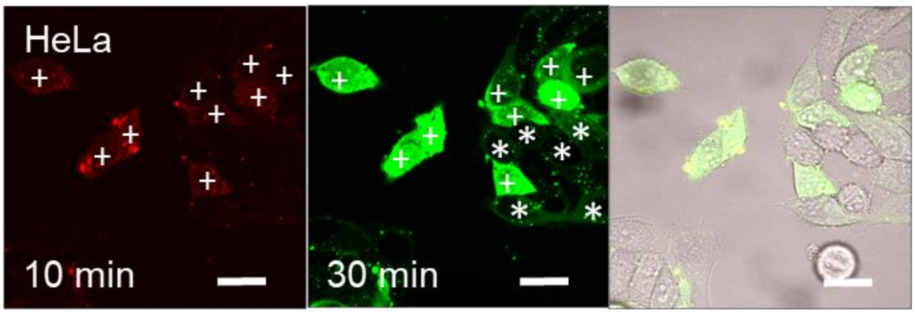

Figure 6a: Live-cell confocal microscopy of 1 μM **tri-Tat A** tagged with AF488 in live HeLa cells, addition of 0.5 μg (40 μM) propidium iodide (PI) 20 min after addition of **tri-Tat A**; image acquired at 30 min after start of experiment. Red channel (left, PI), green channel (middle), and composite red / green / bright field channels (right). Cells that show uptake of both **tri-Tat A** and PI are marked with a cross (+); cells that show uptake of **tri-Tat A** only are marked with an asterisk (*). Only cells with a high level of **tri-Tat A** in the cytosol show uptake of PI. Scale bar: 20 μm.

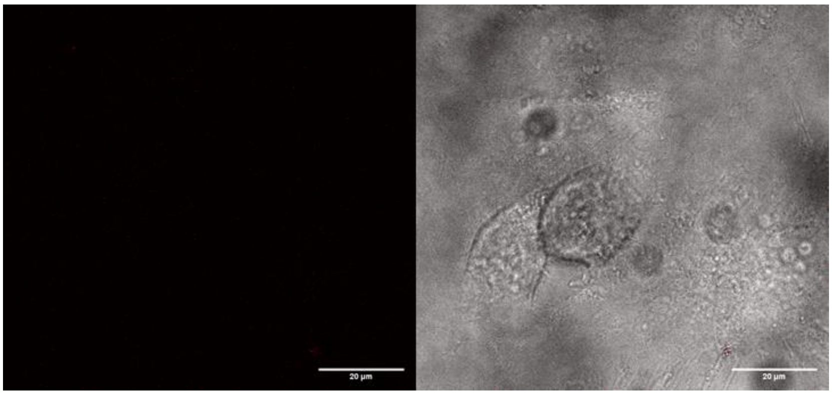

Figure 6b: Live-cell confocal microscopy of 0.5 μg (40 μM) propidium iodide (PI) after 20 min incubation; image acquired at 30 min after start of experiment. Red channel (left, PI) and composite red/bright field channels (right). Cells do not show uptake of PI in the absence of tri-Tat A. Scale bar: 20 μm.

S6.4. Release of tri-cTat B and Fab in HeLa cells

Homogenous distribution of Fab is only observed in cells which have been transduced with **tri-cTat B**, although cells that have been transduced with **tri-cTat B** do not necessarily show homogenous distribution of Fab, which is consistent with a mechanism of co-delivery whereby the peptide trimer forms a vesicle-like body that might but does not always include a detectable amount of Fab, followed by release of either peptide only or both molecules into the cell.

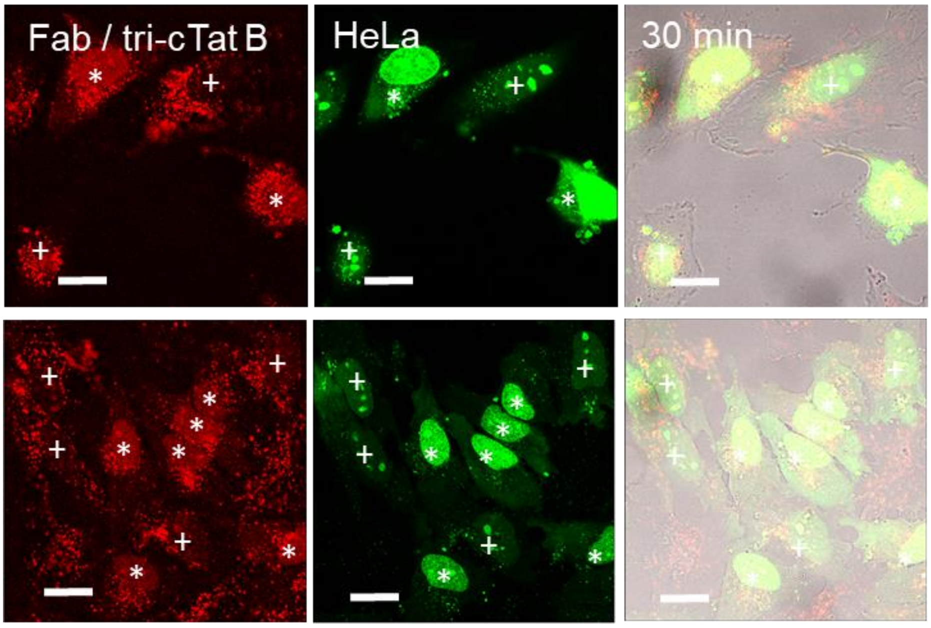

Figure 7: Live-cell confocal microscopy of Fab-AF647 and 1 μM tri-cTat B in live HeLa; images acquired at 30 min after start of experiment. Red channel (left), green channel (middle), and composite red / green / bright field channels (right). Cells that show cytosolic and nuclear accumulation of both Fab-AF647 and **tri-cTat B** are marked with an asterisk (*); cells that show uptake of **tri-cTat B** only are marked with a cross (+). Accumulation of **tri-cTat B** and Fab are always concurrent, but some cells show accumulation of **tri-cTat B** without release of Fab. Scale bar: 20 μm.

S6.5. Control experiment: IgG / Fab uptake into HeLa cells without Tat-trimer treatment

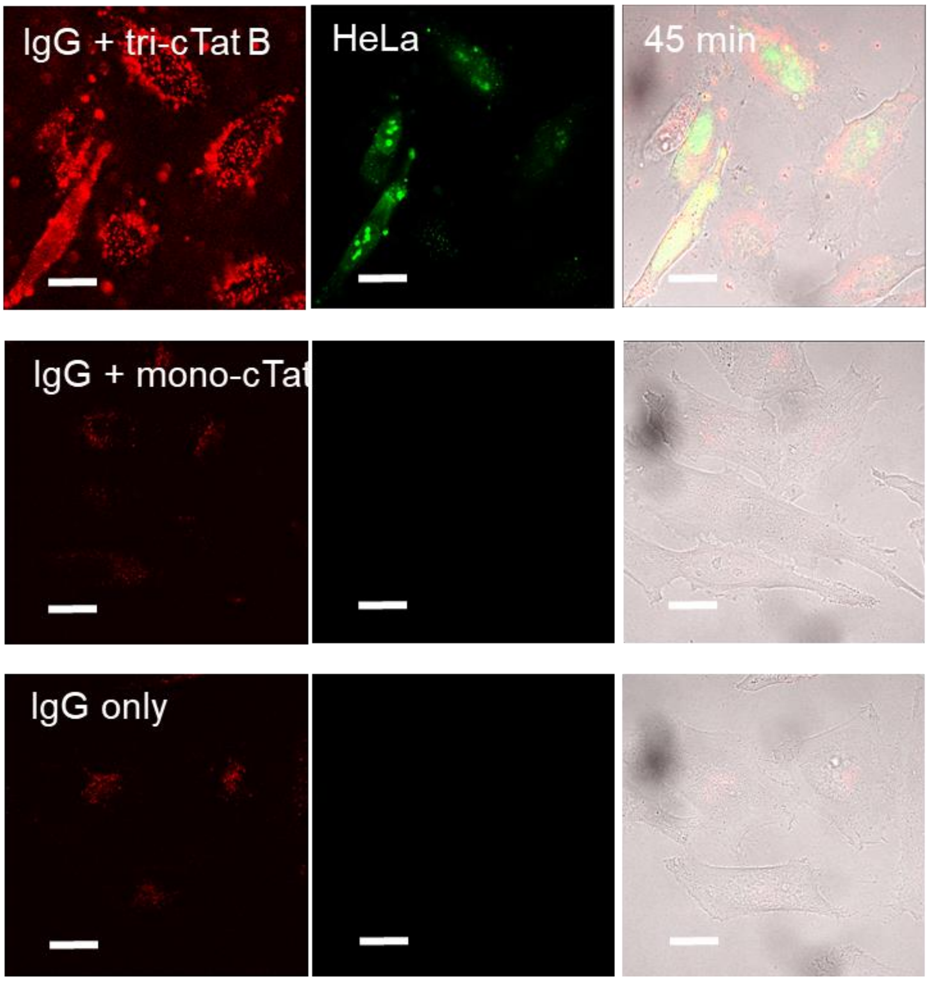

Figure 8a: Live-cell confocal microscopy of IgG-AF647 in live HeLa cells; top row: addition of 1 μM **tri-cTat B** tagged with AF488; middle row: addition of 1 μM **mono-cTat** tagged with AF488; bottom row: antibody only. Images acquired after 45 min of incubation, post-wash. Red channel (left), green channel (middle), and composite red / green / bright field channels (right). Cells treated with antibody only or with antibody and mono-Tat show no uptake or cytosolic accumulation of antibody; **tri-cTat B** is necessary for the delivery of antibody into the cytosol. Scale bar: 20 μm.

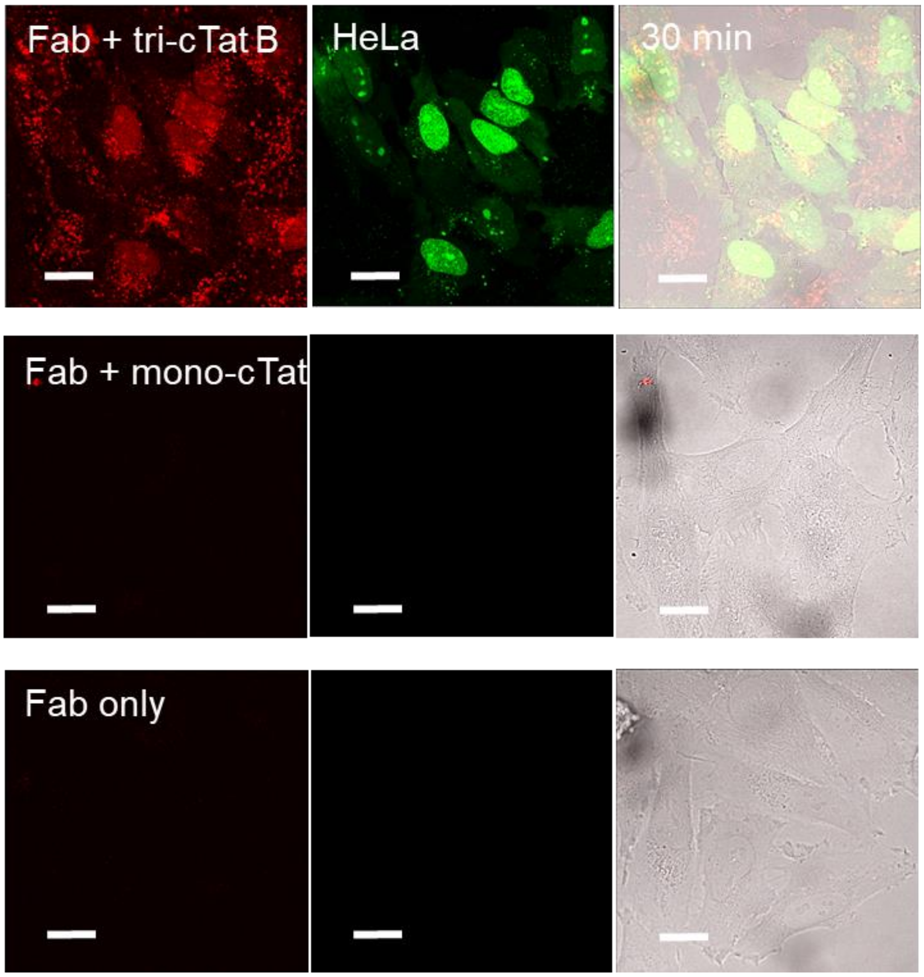

Figure 8b: Live-cell confocal microscopy of Fab-AF647 in live HeLa cells; top row: addition of 1 μM **tri-cTat B** tagged with AF488; middle row: addition of 1 μM **mono-cTat** tagged with AF488; bottom row: antibody fragment only. Images acquired after 30 min of incubation, post-wash. Red channel (left), green channel (middle), and composite red / green / bright field channels (right). Cells treated with antibody fragment only or with fragment and mono-Tat show no uptake or cytosolic accumulation of antibody fragment; **tri-cTat B** is necessary for the delivery of antibody fragment into the cytosol. Scale bar: 20 μm.

S6.6. tri-cTat B (1 μM / 2 μM) delivery in the presence of serum in HeLa cells

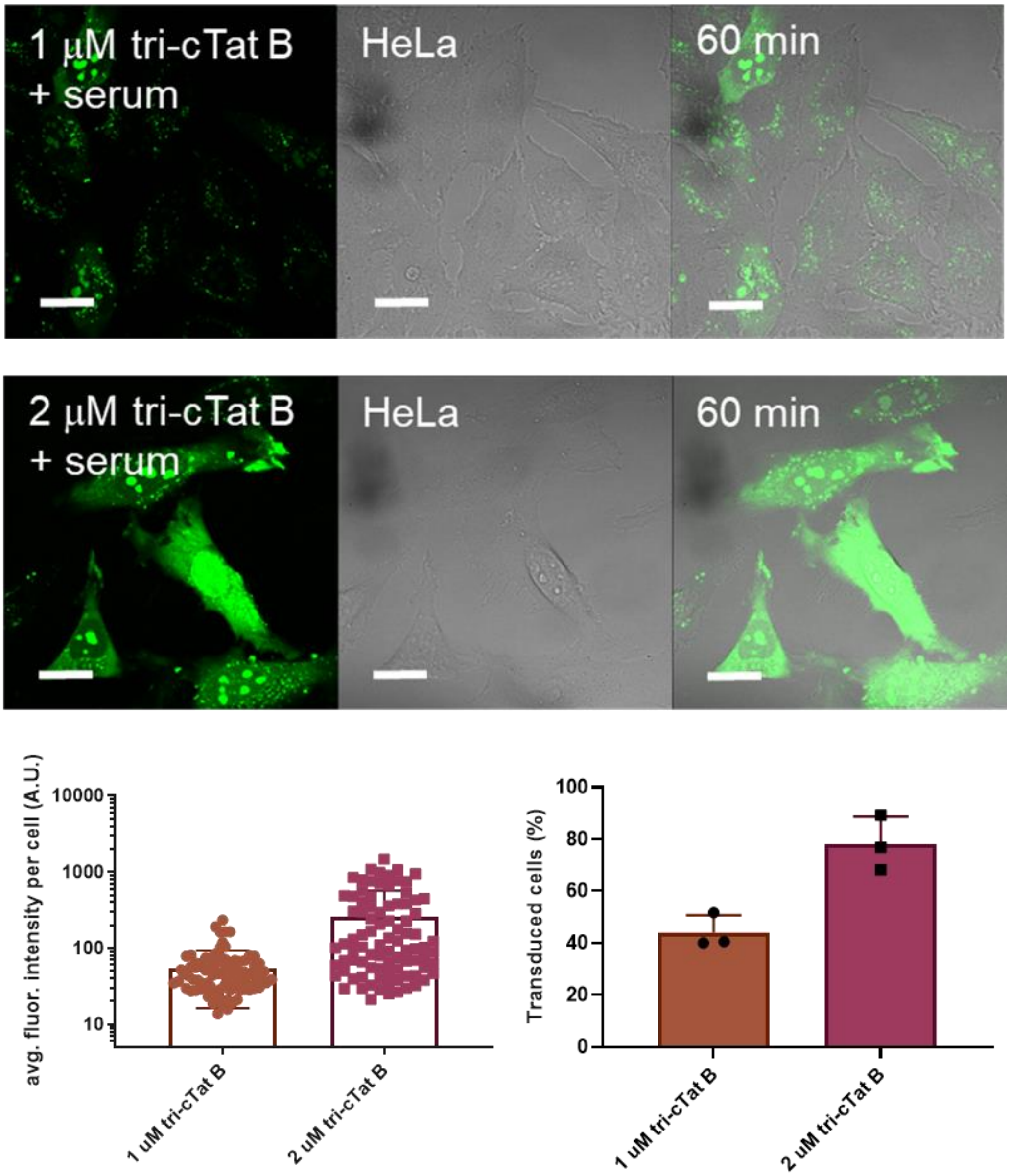

Figure 9: Live-cell confocal microscopy of live HeLa treated with 1 μM (top row) and 2 μM (middle row) tri-cTat B (**17**) tagged with AF488 in serum-supplemented medium for 60 min; imaging post-wash. Quantification of average fluorescence intensity/cell following treatment with 1 μM or 2 μM **tri-cTat B** (**17**) in the presence of serum (bottom left). Quantification of the percentage of transduced cells (scored as positive when showing homogenous cytoplasmic and nucleolar fluorescence) in cells treated with 1 μM (n = 91) and 2 μM (n = 89) **tri-cTat B** (**17**) in the presence of serum (bottom right). Left image = green channel; middle image = brightfield; right image = overlay. Data presented as mean ± standard deviation. Scale bar: 20 μm.

S6.7. Co-delivery of Red Fluorescence Protein (RFP) using tri-cTat B in HeLa cells

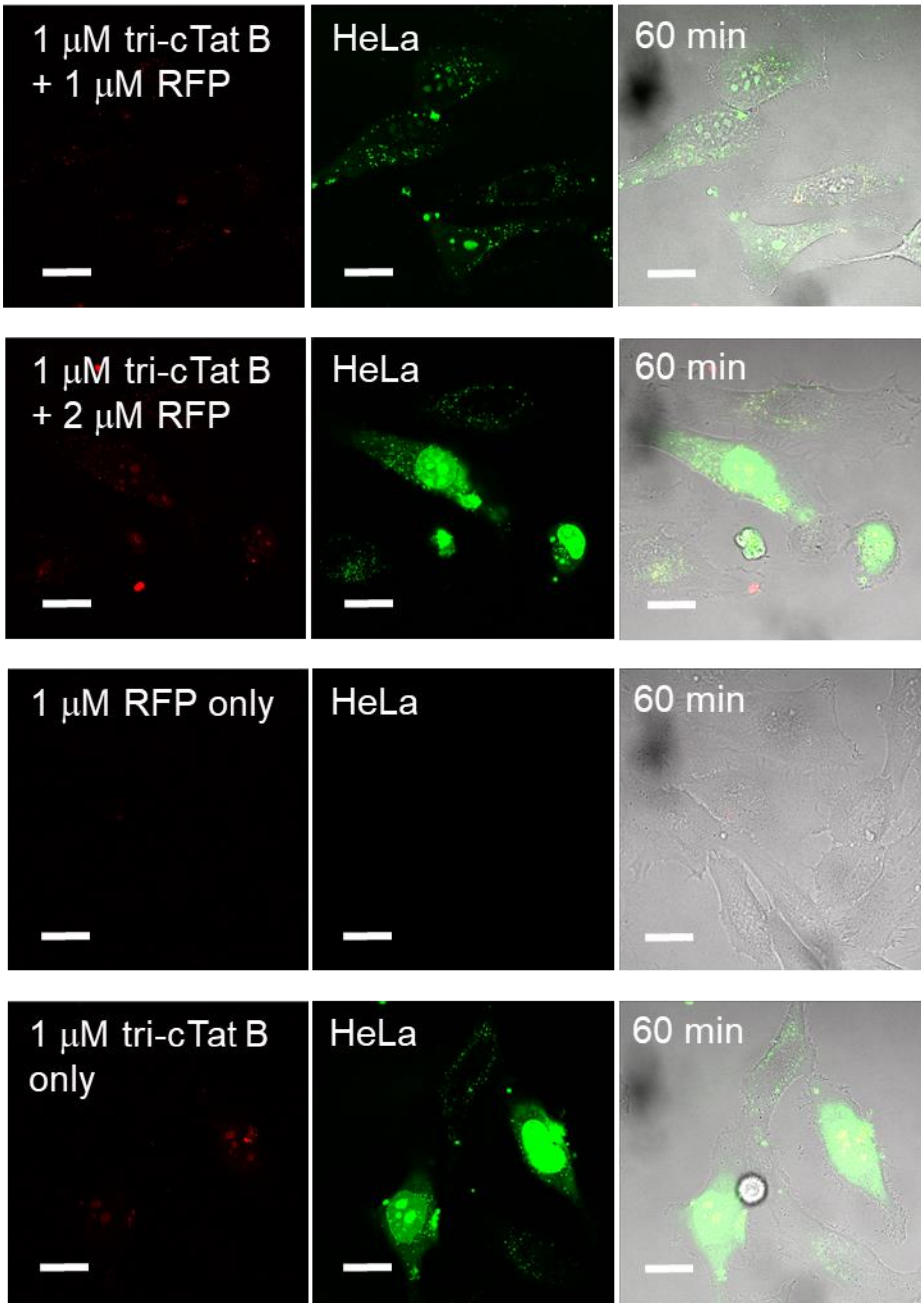

Figure 10: Live-cell confocal microscopy of HeLa cells treated with 1 μM tri-cTat B (17) and 1 μM recombinant red fluorescence protein (RFP) (top row) / 2 μM recombinant RFP (second row) for 30 min in serum free media and imaged 30 min thereafter. Treatment with 1 μM recombinant RFP only (third row) and 1 μM **tri-cTat B** only (bottom row). Left image: red (RFP) channel; middle image: green (**tri-cTat B**) channel; right image: overlay of brightfield, red and green channels. Scale bar: 20 μm.

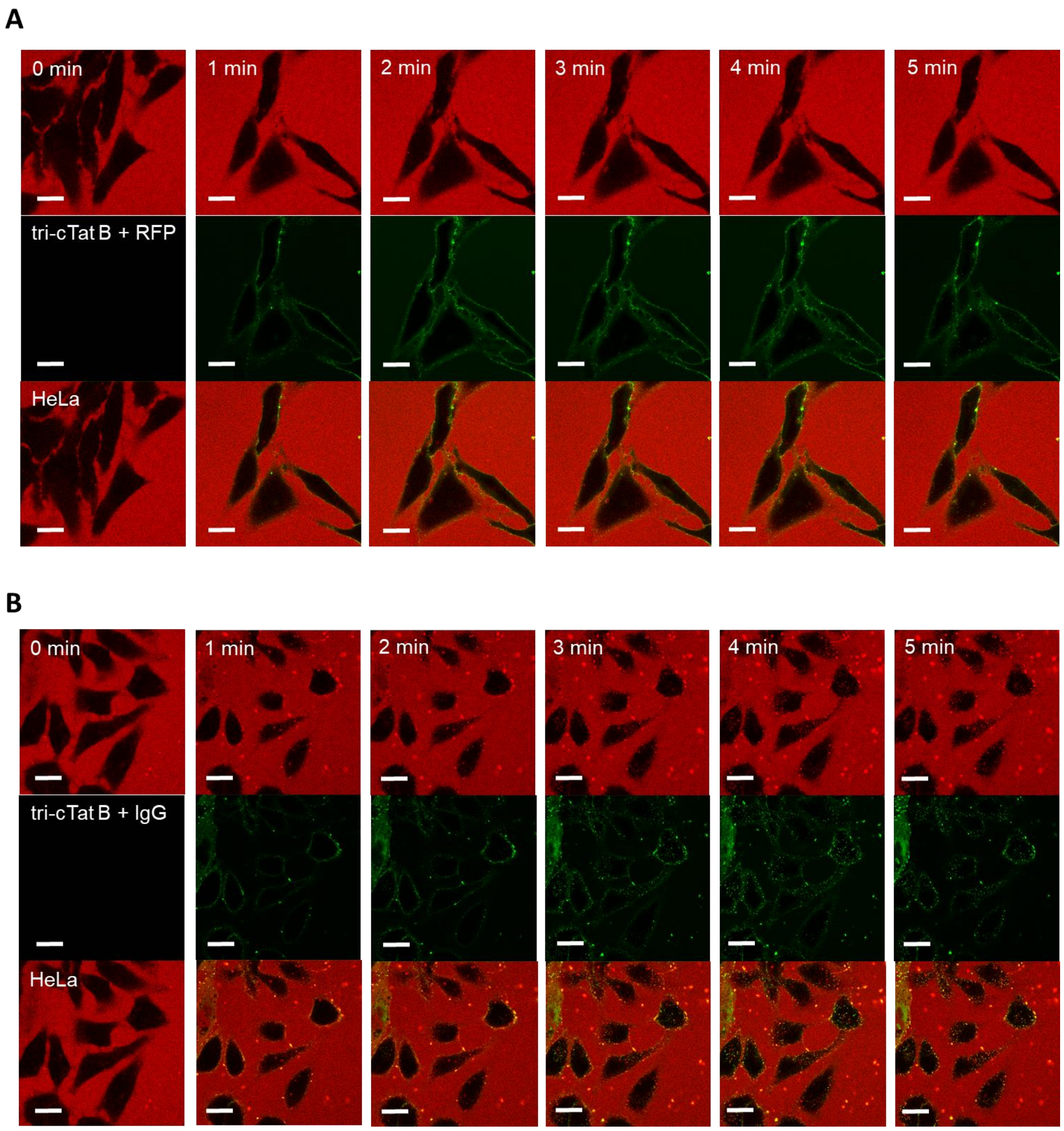

Figure 11: Time course of cargo association with tri-cTat B (17) by live-cell confocal microscopy of live HeLa treated with: (A) 1 μM **tri-cTat B** and 1 μM recombinant red fluorescence protein (RFP); and (B) 1 μM **tri-cTat B** and 166 nM IgG antibody-AF647. Top row of each set of images: red channel (RFP / IgG-AF647); middle row: green channel (tri-cTat B); bottom row: red / green overlay. Leftmost (0 min) images are taken with cargo on the cells, but prior to the addition of **tri-cTat B**; 1, 2, 3, 4, 5 min images are taken at denoted time points after the addition of **tri-cTat B** (without washing). RFP cargo does not associate with **tri-cTat B** and remains entirely homogenous in extra cellular distribution, no membrane accumulation or co-localization into endosomes is observed. IgG-AF647 (while displaying homogenous extracellular distribution prior to the addition of trimer) immediately associates with **tri-cTat B** at the plasma membrane of cells and subsequently co-localizes into endosomes. Some aggregation of IgG in the extracellular media is also observed. Scale bar: 20 μm.

S7. Antibody conjugation

S7.1. IgG antibody conjugation

Mouse IgG from serum (600 μg) was dissolved in 200 μL bicarbonate buffer (pH 8.3) and 100 μg AF647 NHS-ester (Thermo Fisher Scientific) (0.08 μmol; 20eq) in 100 μL H_2O added. The solution was left at 4°C overnight. The reaction mixture was reduced in volume using an Amicon Ultra 0.5 ml centrifugal filter (Ultracel – 30k) (Merck Millipore) spinning at 12000xg for 4 min, followed by purification using a G50 Sephadex column in PBS. Antibody fractions were collected, pooled, volume reduced using Amicon Ultra 0.5 mL centrifugal filter (Ultracel – 30k) and concentration determined using a NanoDrop One (Thermo Fisher Scientific) against BSA standard curve. Yield = 63% (380 μg).

S7.2. Fab fragment conjugation

Mouse IgG Fab fragment (Rockland Inc., US) (400 mg) was dissolved in 200 μl bicarbonate buffer (pH 8.3) and 100 μg AF647 NHS-ester (Thermo Fisher Scientific) (0.08 μmol; 10eq) in 100 μl H_2O added. Purification as described under S7.1. except using Amicon Ultra 0.5 mL centrifugal filter (Ultracel – 10k) (Merck Millipore) and a G25 Sephadex column. Yield = 90% (360 μg).

S.7.3. Anti-β-actin IgG antibody

Monoclonal mouse IgG_{2b} β-actin antibody BA3R (Thermo Fisher Scientific) was conjugated to AF647 was described under S.7.1.

S.7.4. Anti-RFP IgG antibody

Monoclonal mouse IgG_1 RFP antibody RF5R (Thermo Fisher Scientific) was conjugated to AF647 as described under S.7.1.

S7.5. Synthesis of Fab fragment

Whole IgG antibody was Papain digested to cleave Fab fragments using Pierce™ Fab Preparation Kit (Thermo Fisher Scientific) according to manufacturer's instruction. In a typical experiment, 100 μg of mouse IgG was digested for 3 hours, followed by Protein A purification to remove Fc fragments and undigested IgG. Eluted Fab fragments were concentrated using Amicon Ultra 0.5 mL centrifugal filter (Ultracel – 10k) (Merck Millipore) and buffer exchanged into 0.1M sodium bicarbonate buffer, PH 8.3. Optical density at 280 nm was measured using a Nanodrop (Thermo Fisher) and molarity calculated. Successful cleavage of Fab fragment was confirmed by SDS page. The resulting Fab fragment was conjugated to AF647 as described under S.7.2.

S.7.6. Anti-β-actin Fab fragment

100 μg monoclonal mouse IgG_{2b} β-actin antibody BA3R (Thermo Fisher Scientific) was Papain digested and conjugated to AF647 as described under S.7.5. Yield = 42% (28 μg).

S.7.7. Anti-RFP Fab fragment

300 μg monoclonal mouse IgG_1 RFP antibody RF5R (Thermo Fisher Scientific was Papain digested and conjugated to AF647 as described under S.7.5. Yield = 38% (76 μg).

S7.8. Binding of RFP Fab fragment to recombinant RFP

The ability of AF647-conjugated anti-RFP Fab fragment to bind to immobilized recombinant RFP protein was assessed and compared to AF647-conjugated anti-RFP IgG. Duplicate reactions containing 2 μg recombinant RFP (Rockland Inc., US) mixed with LDS sample buffer and reducing agent (ThermoFisher Scientific) were heated to 70 °C for 10 min to denature the protein to increase the proportion of the monomeric

form before loading onto a 4-12% Bis-Tris gel. Molecular weight marker (SeeBlue Plus 2, ThermoFisher) was also loaded. Electrophoresis was performed in MOPS running buffer for 50 min at 200 V. The proteins were blotted onto Immobilon-E membrane for 1 h at room temperature using Transfer Buffer (ThermoFisher Scientific) containing 10% methanol. The blot was incubated in Tris Buffered Saline pH 7.6 containing 0.1% Tween-20 and 5% Bovine Serum Albumin (blocking buffer) for 1 h at room temperature. The blot was then divided in half. One half was incubated in anti-RFP-IgG-AF647 and the other half in anti-RFP-Fab-AF647, both diluted in blocking buffer to 1 μg/mL. Incubations were carried out for 1 h at room temperature on a rocking platform. Blots were washed 3 x 5 minutes with Tris Buffered Saline / 0.1% Tween-20 and imaged using a ChemiDoc Imaging System (Bio-Rad) using the AF647 setting to detect the signal from the antibodies and excluding RFP signal.

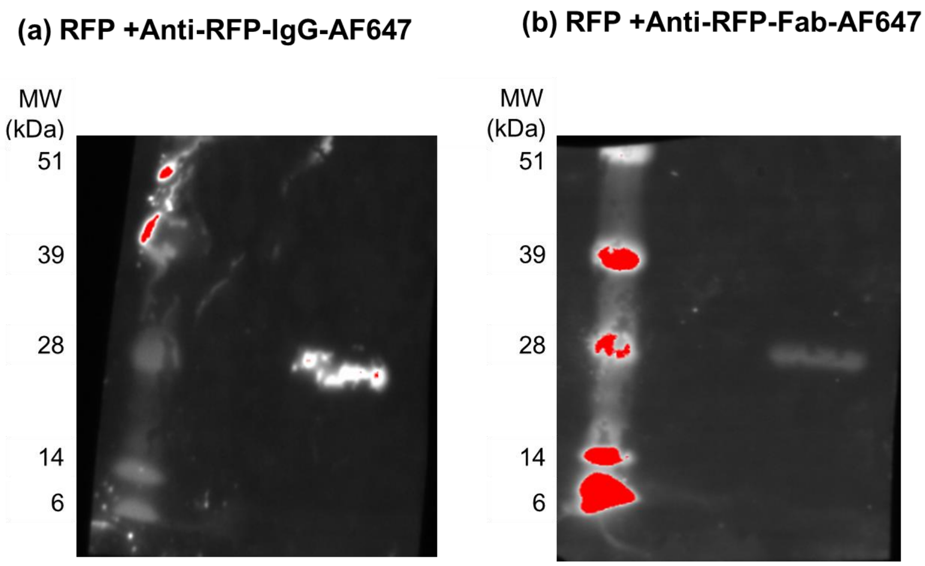

Figure 12a: Immobilized recombinant RFP protein on membrane and incubated with anti-RFP-IgG-AF647 (a) and anti-RFP-Fab-AF647 (b). ChemiDoc Imaging System (Bio-Rad) used to detect AF647 signal; IgG treated membrane exposed for 0.5 seconds; Fab treated membrane exposed for 1.8 seconds. Molecular weight marker shown on the left-hand side. Fab fragment does not retain affinity for recombinant RFP in comparison to IgG.

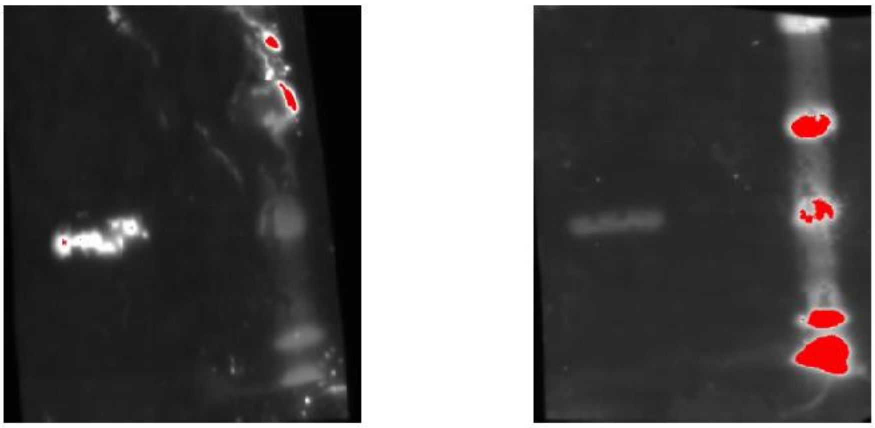

Figure 12b: Unprocessed blot of Figure 12a.

S8. Co-delivery of cargo and co-localization analysis

S8.1. RFP fusion protein transfection of HeLa cells

HeLa cells were transfected with actin-RFP or histone-RFP using a CellLight^{TM} actin-RFP, BacMam 2.0 kit (Thermo Fisher Scientific) according to manufacturer's instructions. Briefly, cells were seeded on greiner 75/25 mm glass bottom cellview cell culture slides (Greiner Bio-One), allowed to adhere for 1 h, treated with 2 μL transfection reagent and left to adhere for a further 24 h. Cells were washed with serum free medium (x3) in preparation for the experiment.

S8.2. IgG / Fab delivery using tri-cTat B

HeLa cells were washed with serum free DMEM supplemented with penicillin / streptomycin. **Tri-cTat B** (1 μM) was added to cells, immediately followed by antibodies (166 nM) / Fab fragment (500 nM) in a final volume of 100 μL. Cells were

maintained at 37 °C in an atmosphere of 5% CO_2 for 30 min, then washed with serum-supplemented DMEM, transferred to the microscopy chamber, and imaged after a further 60 min.

S8.3. Co-localization Analysis

The quantification of co-localization was performed in manually selected regions of interest (ROI) by using the Co-localization Threshold tool in the GDSC plugin for imageJ.[1] The Pearson's and Manders' coefficients were obtained under Costes' automatic threshold and the co-localized pixels are shown as a mask of yellow pixels of constant intensity.[2] The significance of the co-localization parameters (Pearson's and Manders' coefficients) was evaluated with the confined displacement algorithm (CDA), the CDA tool was also provided in the GDSC plugin.[1,3] All results shown present a significant correlation and are co-localized. The magnitude of the Pearson's correlation coefficient is attributed to the local concentration of RFP transfected protein and transduced antibody into cells, which ultimately result in considerable intensity differences between the RFP and the AlexaFluor 647 fluorophores. The Manders' coefficient M2 represents the percentage of antibody that overlaps with β-actin RFP or histone RFP. The values of R and M2 were calculated based on an average of three ROI for each of the different targets.

S8.4. Additional co-localization images

S8.4.1. Actin stress Fibres

The role of actin dynamics in endocytosis in mammalian cells is an active field of research. Actin plays an indirect role in endocytosis by local polymerization during the formation of endocytic carriers, leading to association of endosomes with small actin polymers.[4,5] Experiments were designed to avoid this ambiguity by imaging at the lowest confocal plane in the cell, where actin stress fibres are found. Stress fibres are

contractile actomyosin bundles found in many cultured non-muscle cells and play a central role in cell adhesion and morphogenesis. Their role in the cell is exclusively mechanotransductive[6], and although they can be unmade and subsequently polymerize at endosomes, stress fibres themselves do not participate in endocytosis or endosome trafficking. Imaging at confocal planes, close to the cell culture slide can lead to high background signal. Images below are three different focal planes of the same cell; Image B is shown in the manuscript (Fig. 6b) – two stress fibres are clearly visible at the top and bottom of the cell; Image A was acquired at a lower focal plane and shows more stress fibres, but high background from the culture slide in the red channel; Image C was acquired at a higher focal plane and shows no stress fibres.

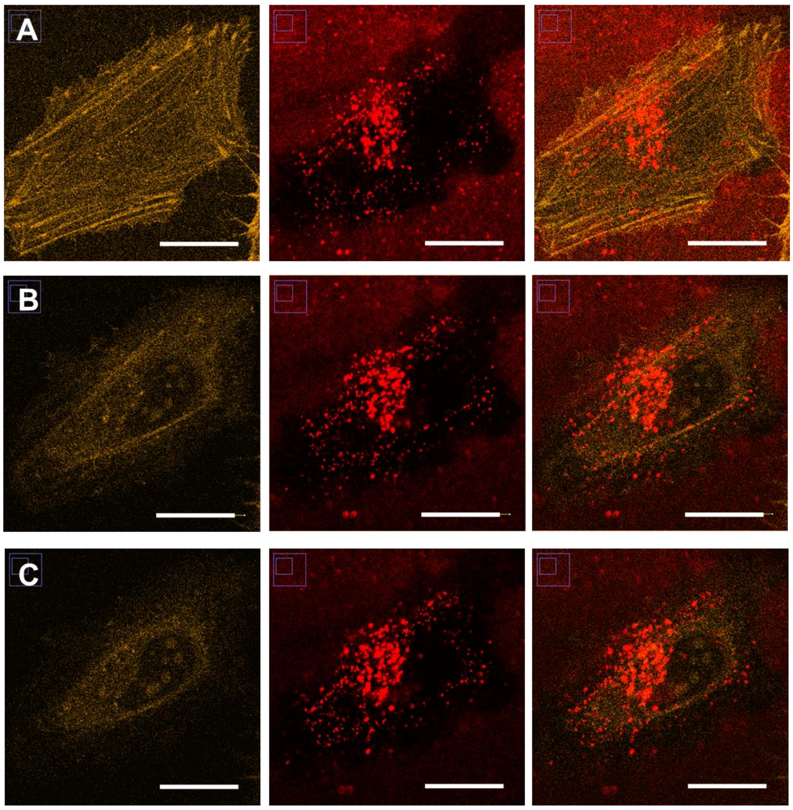

Figure 13: Co-delivery of functional β-actin antibody in live HeLa cells. HeLa cells were transfected with actin-RFP. 90 min post-wash live cell confocal microscopy images of a cell treated with 166 nM anti-β-actin mouse IgG_{2b} AF647 conjugate (β-actin-IgG-AF647) and 1 μM **tri-cTat B** for 30 min. (**A**) Actin stress fibres at lowest confocal plane; (**B**) Some stress fibres at higher focal plane; (**C**) No actin stress fibres at focal plane in the middle of cell. Left = orange

channel (RFP fusion protein); middle = red channel (AF647); right = orange / red overlay; scale bar: 20 μm.

S8.4.2. Co-localization of RFP labeled actin stress fibres with anti-RFP IgG β-actin stress fibres labeled with RFP can be targeted using anti-RFP IgG (mouse, IgG_1).

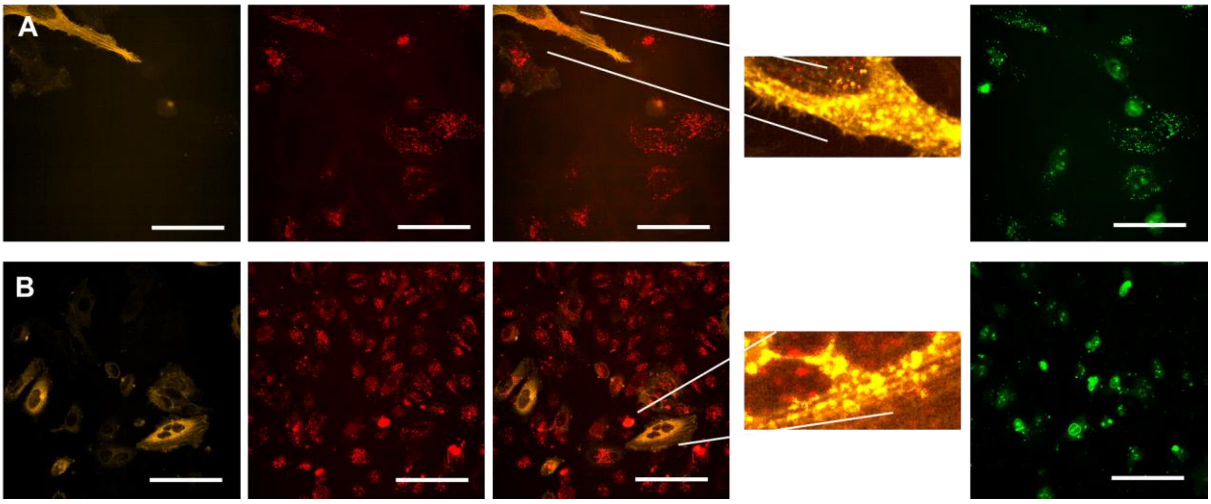

Figure 14: Co-delivery of functional antibodies in live HeLa cells. HeLa cells were transfected with actin-RFP. 90 min post-wash live cell confocal microscopy images of cells treated with 166 nM anti-RFP mouse IgG_1 AF647 conjugate (RFP-IgG-AF647) and 1 μM **tri-cTat B** for 30 min. From the left: orange channel (RFP fusion protein); red channel (AF647); orange / red overlay; Co-localization panel: co-localized pixels are shown as a mask of yellow pixels of constant intensity and all results shown present a significant correlation and are co-localized; green channel (**tri-cTat B**); scale bar: 20 μm.

S8.4.3. Additional images anti-β-actin IgG / actin stress fibre co-localization

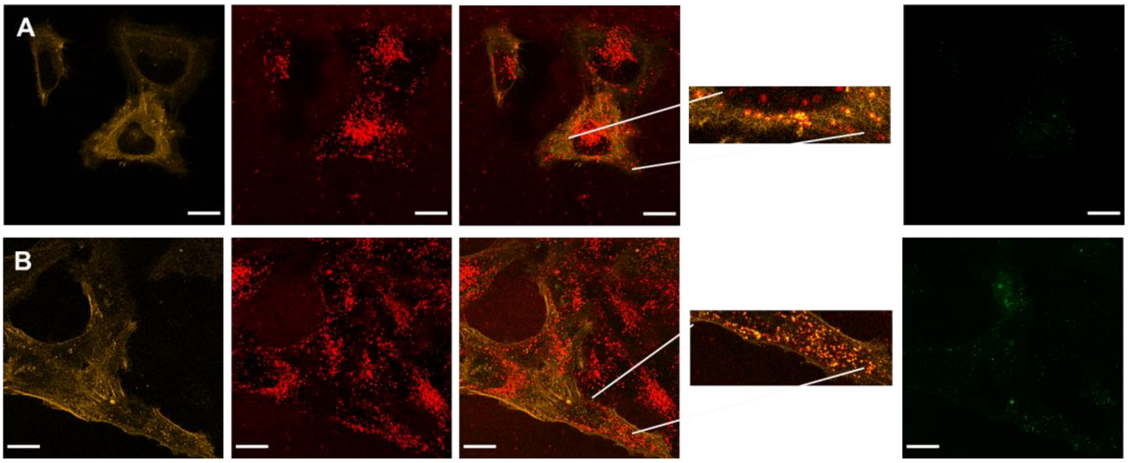

Figure 15: Co-delivery of functional antibodies in live HeLa cells. HeLa cells were transfected with actin-RFP. 90 min post-wash live cell confocal microscopy images of cells treated with 166 nM anti-β-actin mouse IgG_{2b} AF647 conjugate (β-actin-IgG-AF647) and 1 μM **tri-cTat B** for 30 min. From the left: orange channel (RFP fusion protein); red channel (AF647); orange / red overlay; Co-localization panel: co-localized pixels are shown as a mask of yellow pixels of constant intensity and all results shown present a significant correlation and are co-localized; green channel (**tri-cTat B**); scale bar: 20 μm.

S8.4.4. Additional Images anti-RFP IgG / histone-RFP co-localization

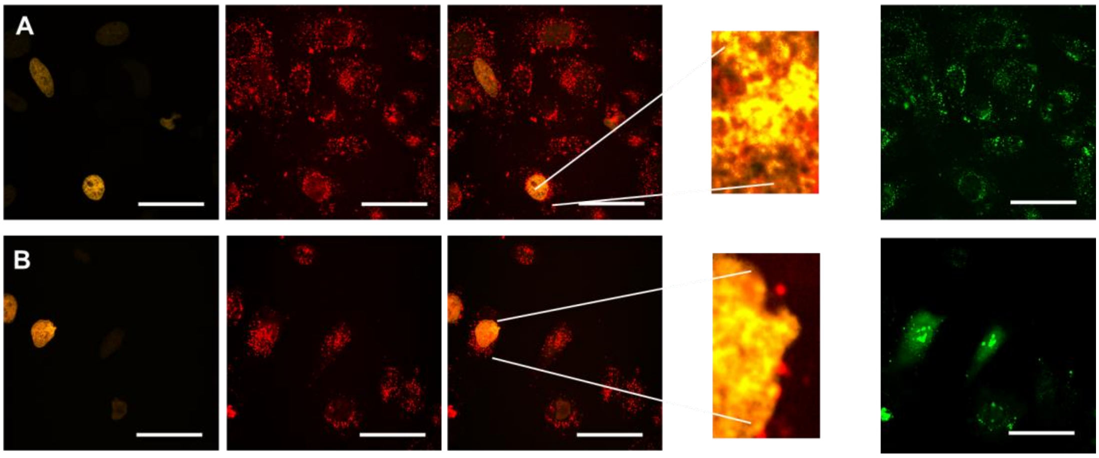

Figure 16: Co-delivery of functional antibodies in live HeLa cells. HeLa cells were transfected with histone-RFP. 90 min post-wash live cell confocal microscopy images of cells treated with 166 nM anti-RFP mouse IgG_1 AF647 conjugate (RFP-IgG-AF647) and 1 μM **tri-cTat B** for 30 min. From the left: orange channel (RFP fusion protein); red channel (AF647); orange / red overlay; Co-localization panel: co-localized pixels are shown as a mask of yellow pixels of constant intensity and all results shown present a significant correlation and are co-localized; green channel (tri-cTat B); scale bar: 20 μm.

S8.4.5. Whole cell co-localization analysis

The Costes approach to thresholding has the advantage of being reproducible and objective as it does not require operator input. However, challenges may be encountered due to variation in the background for a larger region of the image or the whole image so no one background is correct. The difference in intensities of two fluorophores, as is the case of two labeled proteins presenting unequal labeling efficiencies, can also be challenging when determining the threshold through this approach.[7,8] To ensure the most accurate thresholding result from the studied subcellular structures, we selected subsections of the cell and ROIs that show reasonably homogenous background and intensity distributions of the two fluorophores. The analysis of whole cells has also been included below.

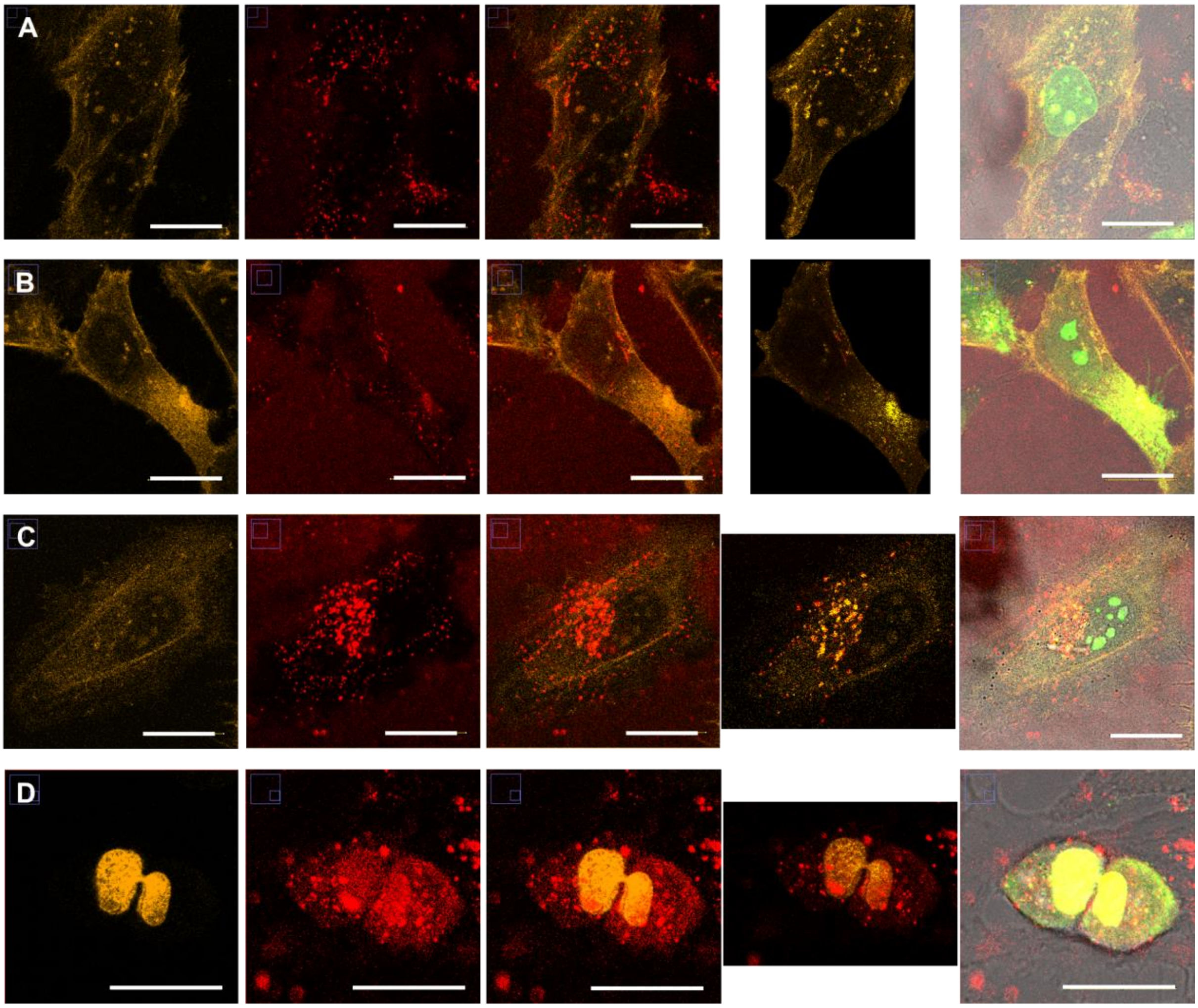

Figure 17: Co-delivery of functional antibodies and antibody fragments in live HeLa cells. HeLa cells were transfected with actin-RFP (**A - C**) or histone-RFP (**D**). 90 min post-wash live cell confocal microscopy images of cells treated with (**A, B**) 500 nM anti-β-actin mouse Fab AF647 conjugate (β-actin-Fab-AF647) and 1 μM tri-cTat B for 30 min; (**C**) 166 nM anti-β-actin mouse IgG2b AF647 conjugate (β-actin-IgG-AF647) and 1 μM tri-cTat B for 30 min; (**D**) 166 nM anti-RFP mouse IgG1 AF647 conjugate (RFP-IgG-AF647) and 1 μM tri-cTat B for 30 min. G = green channel (tri-cTat B); O = orange channel (RFP fusion protein); R = red channel (AF647); Co-localization panel: co-localized pixels are shown as a mask of yellow pixels of constant intensity and all results shown present a significant correlation and are co-localized; BF = brightfield; scale bar: 20 μm.

S8.5. Pearson & Manders' coefficient Summary

Table 1: Summary of Pearson and Manders' coefficients for co-localization images shown in main manuscript and supporting information S8.4.

**Main Manuscript Fig. 6**	**R**	**M1**	**M2**
Fig. 6a – anti-β-actin Fab / β-actin-RFP (top row)	0.133	0.083	0.401
Fig. 6a – anti-β-actin Fab / β-actin-RFP (bottom row)	0.104	0.066	0.346
Fig. 6b – anti-β-actin IgG / β-actin-RFP	0.126	0.044	0.332
Fig. 6c – anti-RFP IgG / histone-RFP	0.268	0.474	0.452
**SI Fig. 14 – anti-RFP IgG / β-actin-RFP**			
Image A (top row)	0.406	0.676	0.600
Image B (bottom row)	0.372	0.312	0.491
**SI Fig. 15 – anti-β-actin IgG / β-actin-RFP**			
Image A (top row)	0.124	0.037	0.442
Image B (bottom row)	0.131	0.067	0.379
**SI Fig. 16 – anti-RFP / histone-RFP**			
Image A (top row)	0.570	0.654	0.678
Image B (bottom row)	0.425	0.956	1.000
**SI Fig. 17 – whole cell analysis**			
Image A – anti-β-actin Fab / β-actin-RFP (6a – top)	0.084	0.044	0.352
Image B – anti-β-actin Fab / β-actin-RFP (6a – bottom)	0.127	0.243	0.106
Image C – anti-β-actin IgG / β-actin-RFP (6b)	0.126	0.044	0.332
Image D – anti-RFP IgG / histone-RFP (6c)	0.237	0.191	0.029
**SI Fig. S4a, S4b, S5a - Rab5a co-localization**			
S4a - tri-Tat A	0.264	0.188	0.471
S4b - tri-Tat B	0.242	0.511	0.519
S5a - tri-cTat B	0.474	0.713	0.676

S9. Quantitative Analysis of mechanism of uptake

To analyze and compare mechanism of cell entry in multiple cells, we selected five cells per treatment group from two independent experiments. Plot profile lines were drawn through the cells (indicated with yellow lines in the images below) so that two membrane regions, cytosolic region, nuclear region, and nucleolar regions were represented at each time point. Green channel plot profiles were generated for each

time point from 1 min to 15 min (60 sec intervals) and ROI corresponding to membrane, cytosol, nucleus and nucleoli designated using the brightfield image. ROI’s were adjusted for each time point to allow for cell movement and small variations in the confocal plane. Membrane ROIs all measure 1 μm in length to ensure consistency. Cytosolic ROI’s exclude signal resulting from endosomes to ensure that these are representative of free peptide in the cytosol. Nuclear ROIs exclude signal resulting from nucleoli. All data is represented as average fluorescence signal in the designated ROI. The images below show green channel / brightfield overlay and green channel plot profile for each cell and time point used for the analysis shown in Fig. 3e-3h of the main manuscript.

(A) tri-Tat A – Cell I

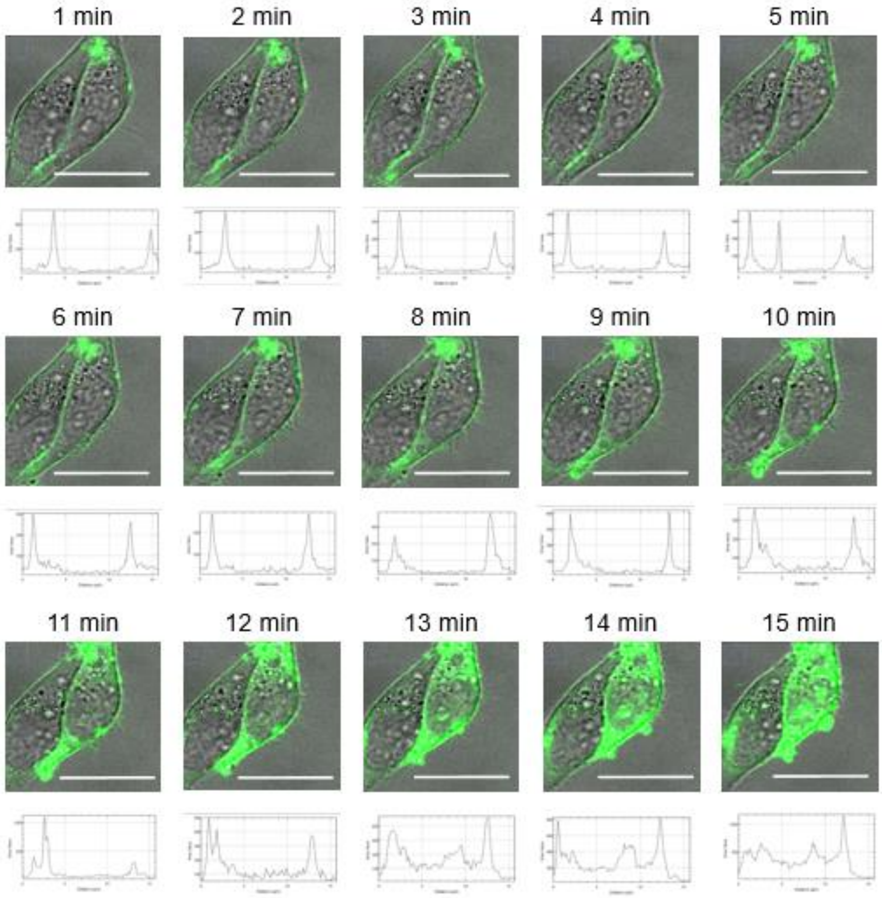

(B) tri-Tat A – Cell II

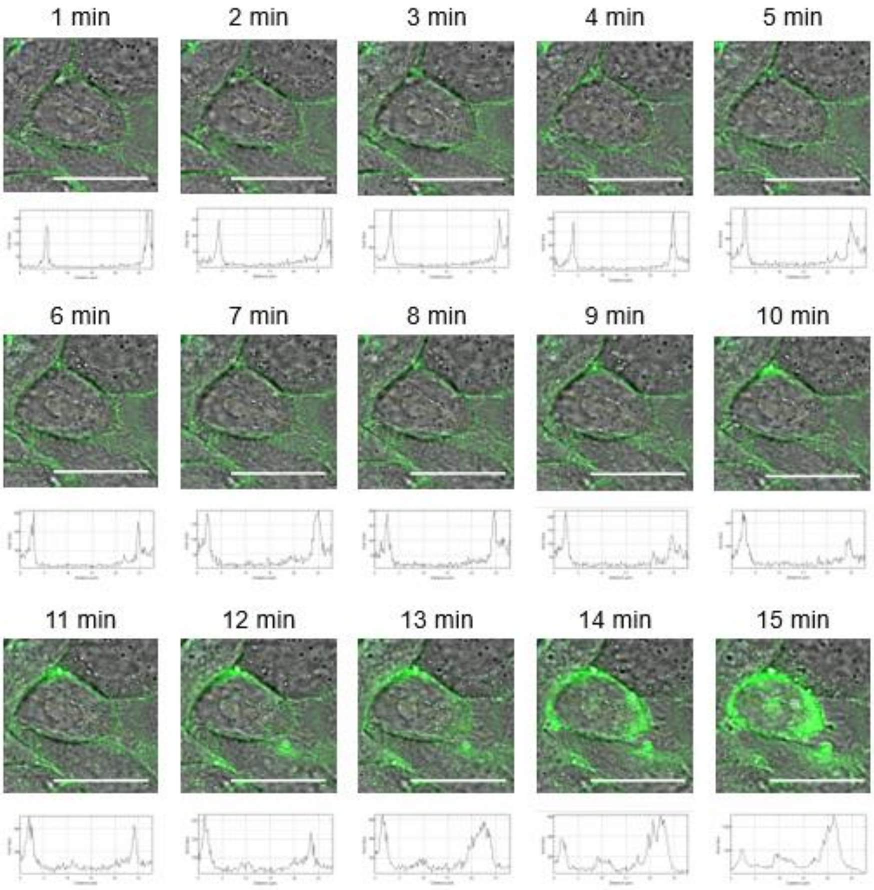

(C) tri-Tat A – Cell III

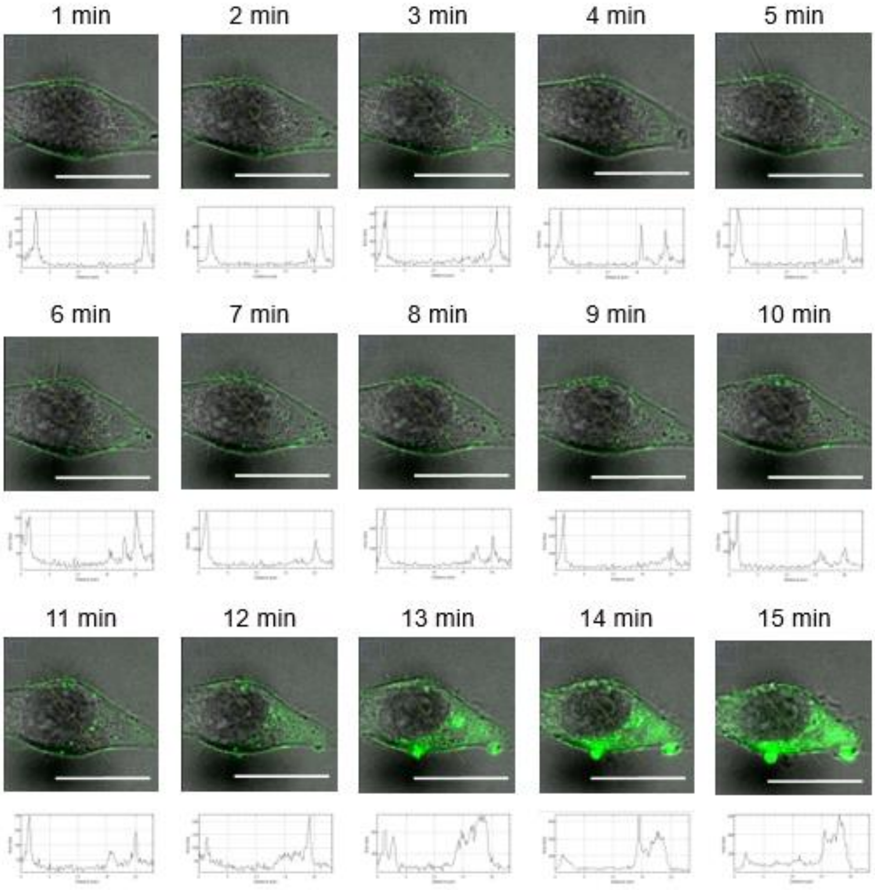

(D) tri-Tat A – Cell IV

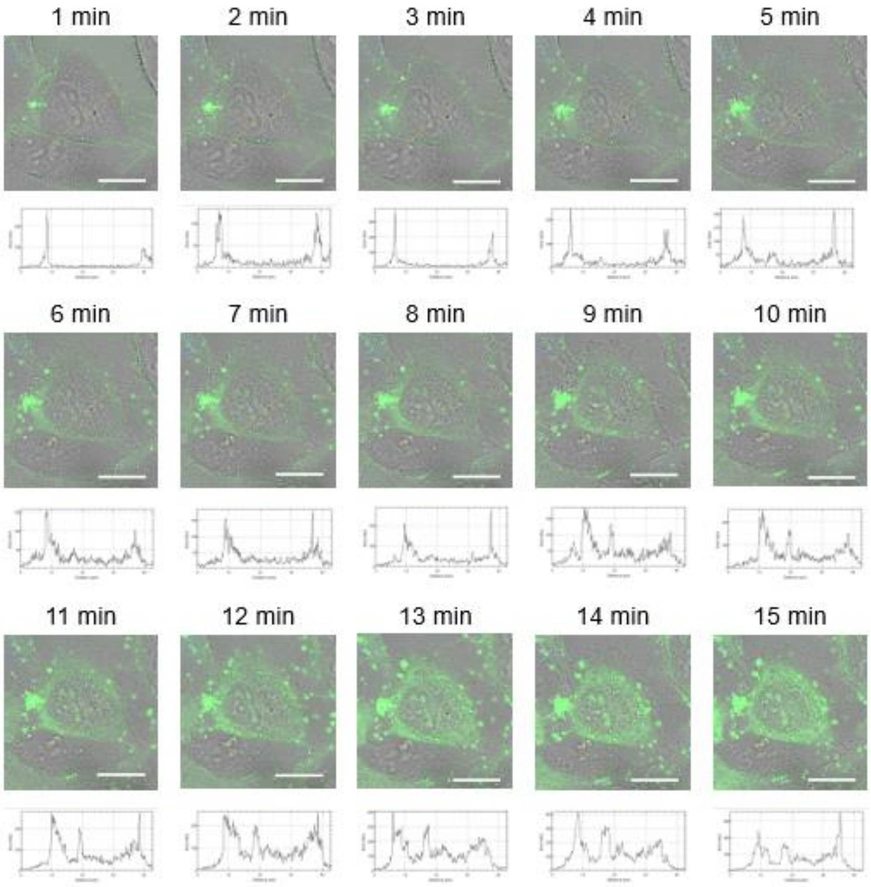

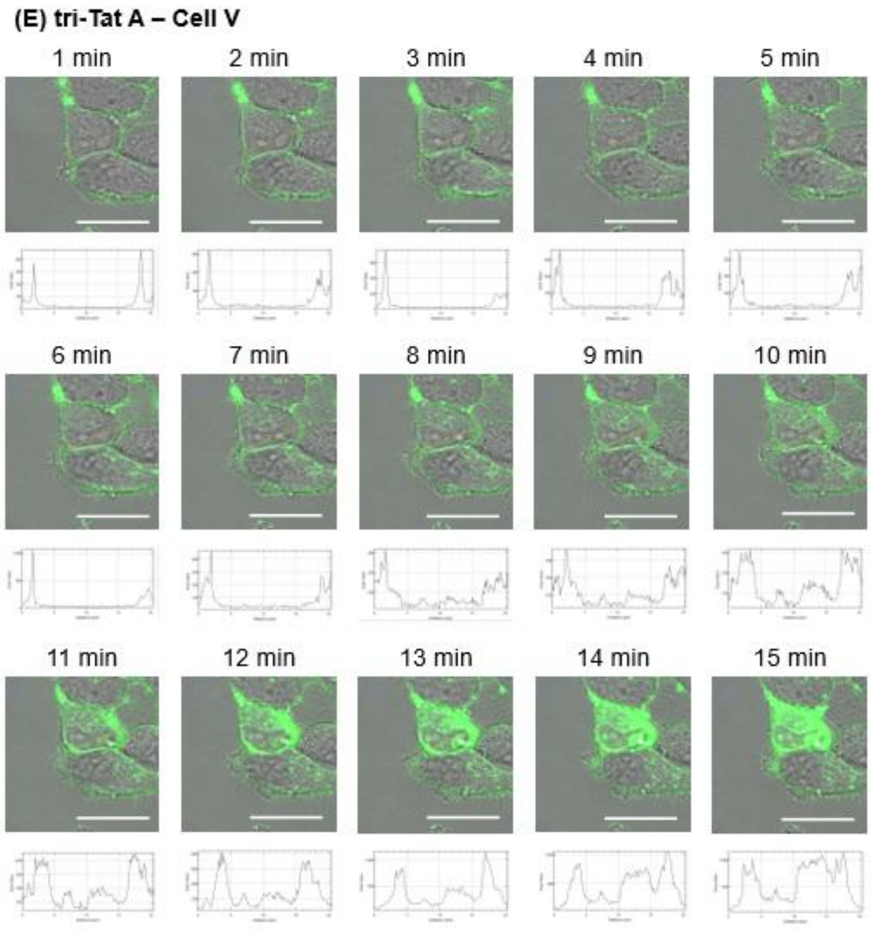
(E) tri-Tat A – Cell V
1 min
2 min
3 min
4 min
5 min
6 min
7 min
8 min
9 min
10 min
11 min
12 min
13 min
14 min
15 min

(F) tri-cTat B – Cell I

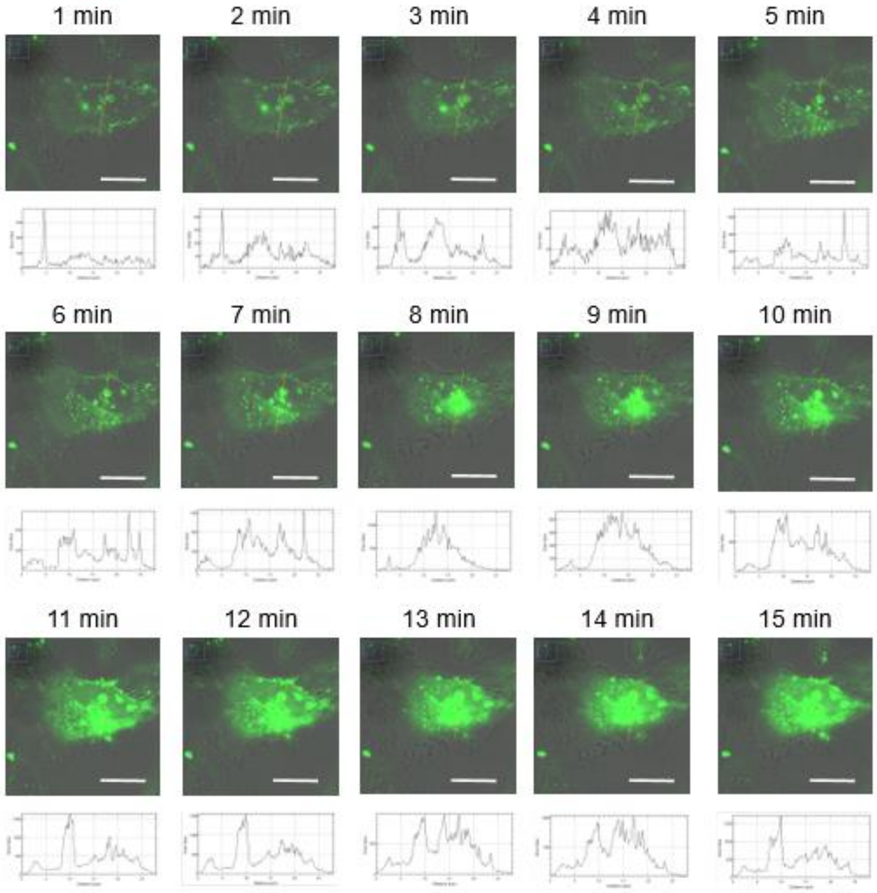

(G) tri-cTat B – Cell II

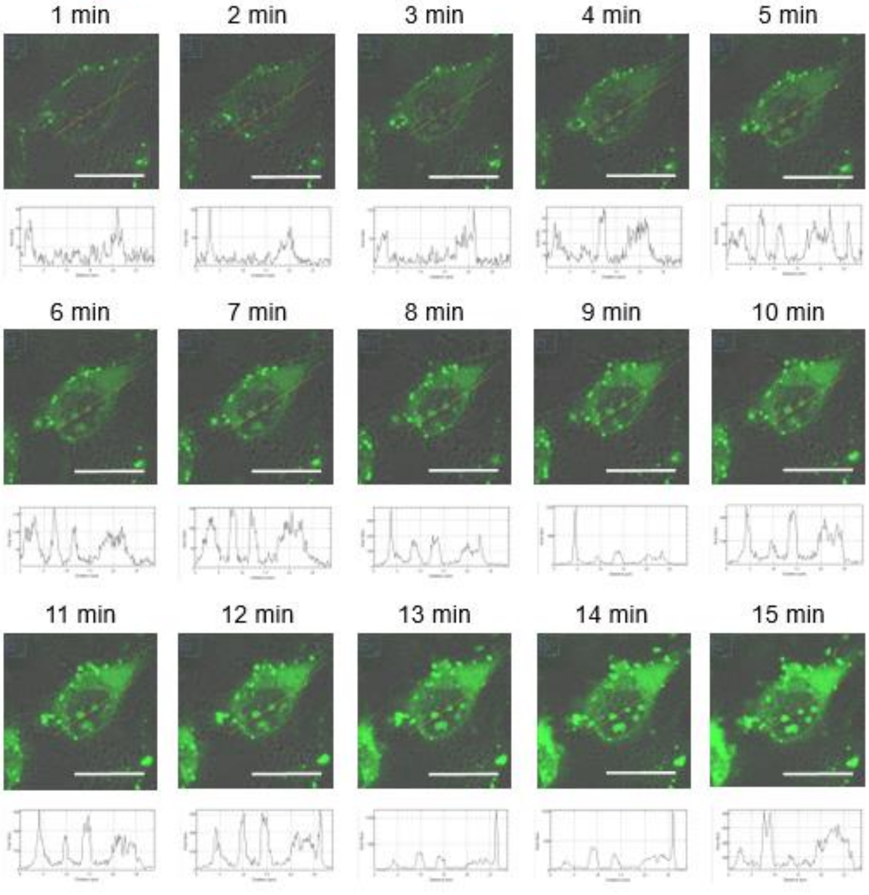

(H) tri-cTat B – Cell III

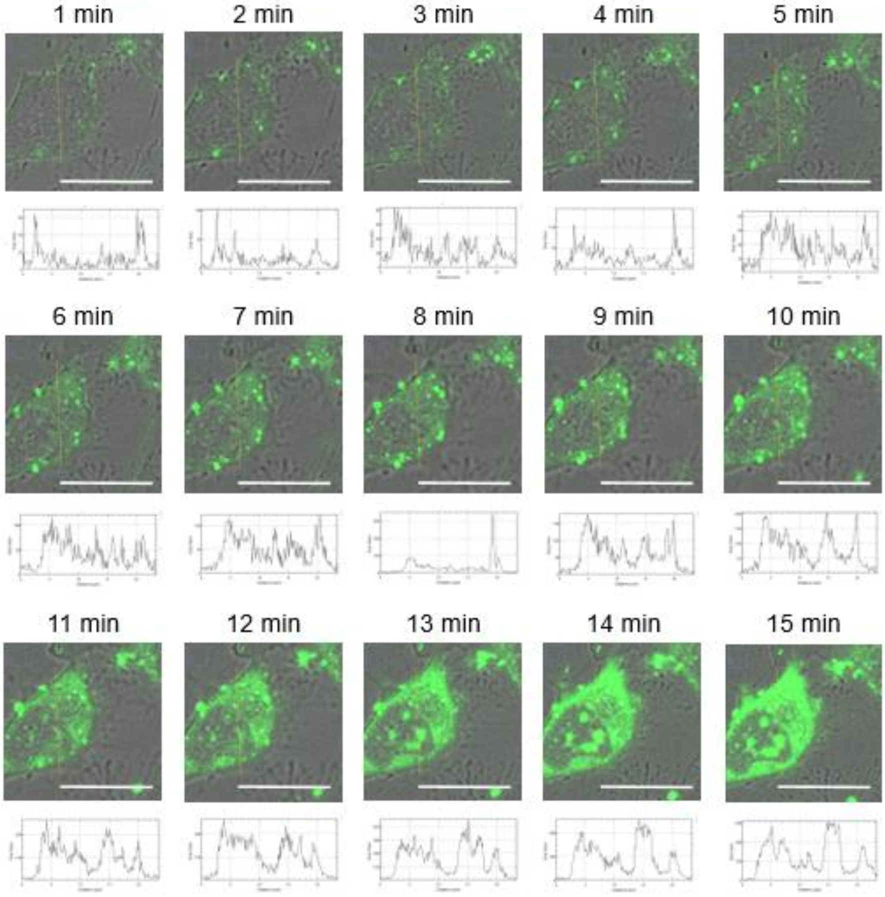

(I) tri-cTat B – Cell IV

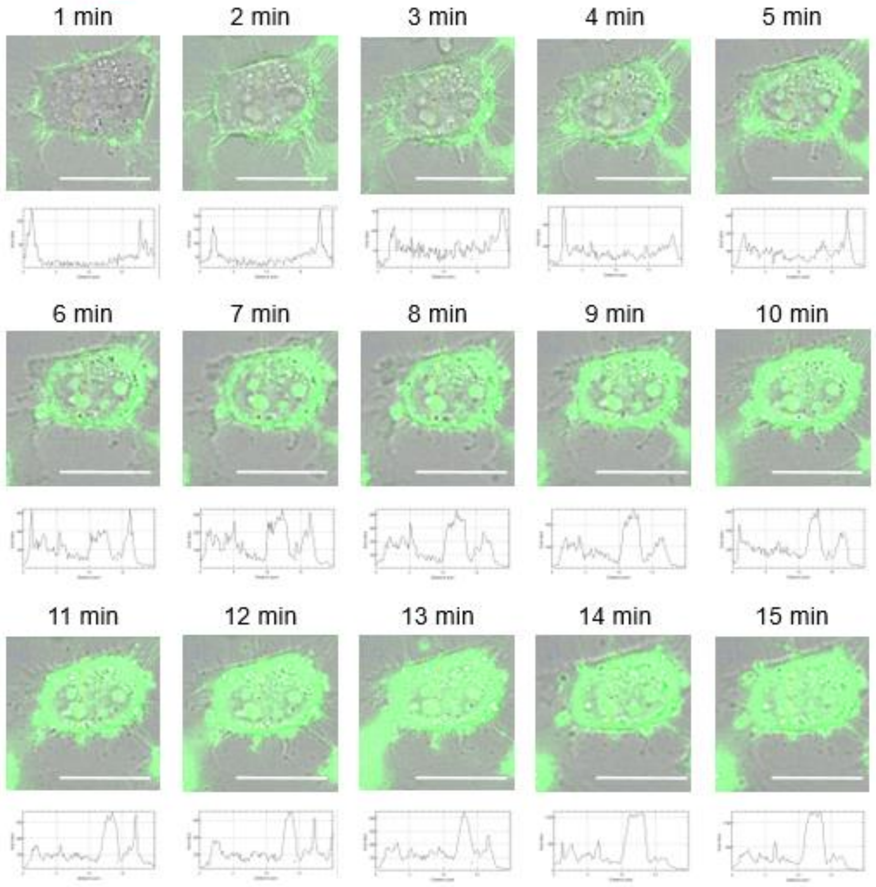

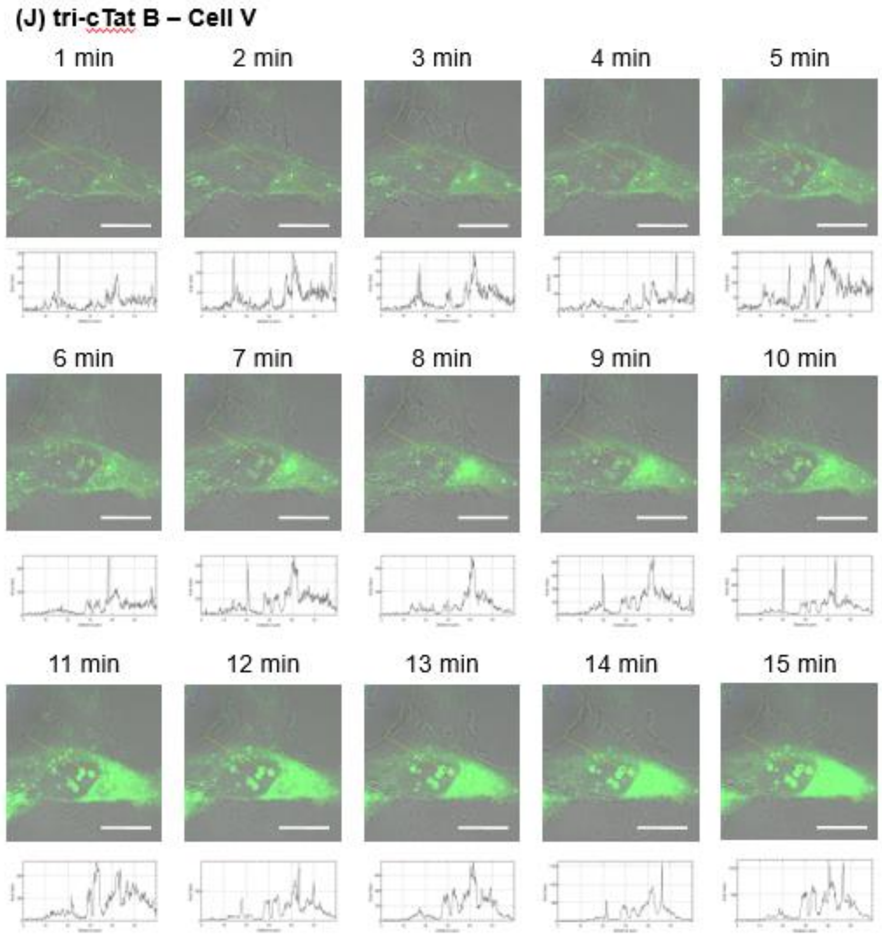

Figure 18 A-J: Green channel / brightfield overlay images of cells used in the quantitative cell uptake analysis. Yellow line indicates position of the plot profile analysis; corresponding green channel plot profile below. Cells **A – E** treated with tri-Tat A; **F – J** treated with tri-cTat B. Intensity of the green channel in images of tri-cTat B treated cells is increased by a factor of 2, for clarity.

Table S2: p-values of t-test analysis comparing average fluorescence intensity of **tri-Tat A** and **tri-cTat B** in the designated ROI for a specific time point. Significant results ($P < 0.05$) highlighted.

time (min)	p values			
	Membrane	Cytosol	Nucleus	Nucleoli
1	0.00162	0.52737	0.51028	0.10595
2	0.00178	0.36096	0.24916	0.09387
3	0.00122	0.20496	0.2499	0.06801
4	0.00073	0.04958	0.17814	0.00082
5	0.00045	0.0257	0.13247	0.02761
6	0.02627	0.02562	0.10873	0.02223
7	0.00483	0.01819	0.08058	0.02317
8	0.00326	0.07094	0.06756	0.08701
9	0.01551	0.13616	0.20937	0.06942
10	0.01368	0.30919	0.27532	0.06123
11	0.03877	0.41715	0.20864	0.07922
12	0.03665	0.95215	0.49143	0.06956
13	0.0075	0.53009	0.65937	0.04738
14	0.01814	0.20519	0.91264	0.17241
15	0.03017	0.07931	0.31832	0.21145

S10. Proximity ligation assay protocol

The Duolink in situ red starter kit (Sigma-Aldrich) was used with mouse/rabbit secondary detection antibodies. The protocol recommended by the manufacturer was followed after the co-delivery processed was completed. HeLa cells were seeded at $20x10^3$ cells in greiner 75/25 mm glass bottom cellview cell culture slides (Greiner Bio-One) and allowed to adhere overnight. The cells were treated with H2B-AF488 (166 nM) and $cTat_3$-alkyne (2 μM) for 1h at 37 ˚C, 5% CO_2 and washed thoroughly (3 x PBS). The cells were then fixed 30 min post washing using 4% paraformaldehyde (15 min incubation time) and permeabilized using triton X-100 (0.1%) for 10 min at room temperature. After washing with PBS, the cells were incubated with Duolink blocking

solution for 1 h at 37 ˚C, 5% CO_2. H2A.Z antibody was incubated at 1:100 dilution in necessary wells for 1 h followed by incubation with secondary antibodies (plus and minus probes) for 1 h at 37 ˚C, 5% CO_2. Ligation was carried out for 30 min and amplification for 3.5 h in the incubator. After the final washes, the nuclei were stained using Hoechst 33342 (1 µg/mL) for 10 min at room temperature.

S11. References

1 Herbert, A. *Available from:* http://www.sussex.ac.uk/gdsc/intranet/microscopy/UserSupport/AnalysisProtocol/imagej/colocalisation, <

2 Costes, S. V. *et al.* Automatic and quantitative measurement of protein-protein colocalization in live cells. *Biophys. J.* **86**, 3993-4003, (2004).

3 Ramirez, O., García, A., Rojas, R., Couve, A. & Härtel, S. Confined displacement algorithm determines true and random colocalization in fluorescence microscopy. *J. Microsc.* **239**, 173-183, (2010).

4 Hinze, C. & Boucrot, E. Local actin polymerization during endocytic carrier formation. *Biochem. Soc. Trans.* **46**, 565-576, (2018).

5 Simonetti, B. & Cullen, P. J. Actin-dependent endosomal receptor recycling. *Curr. Opin. Cell. Biol.* **56**, 22-33, (2019).

6 Tojkander, S., Gateva, G. & Lappalainen, P. Actin stress fibers--assembly, dynamics and biological roles. *J. Cell Sci.* **125**, 1855-1864, (2012).

7 Bolte, S. & Cordelieres, F. P. A guided tour into subcellular colocalization analysis in light microscopy. *J. Microsc.* **224**, 213-232, (2006).

8 Dunn, K. W., Kamocka, M. M. & McDonald, J. H. A practical guide to evaluating colocalization in biological microscopy. *Am. J. Physiol. Cell. Physiol.* **300**, C723-742, (2011).
